# Recent advances in the application of indoles in multicomponent reactions

**DOI:** 10.1039/c7ra13321a

**Published:** 2018-03-28

**Authors:** Ghodsi Mohammadi Ziarani, Razieh Moradi, Tahereh Ahmadi, Negar Lashgari

**Affiliations:** Department of Chemistry, Alzahra University Tehran Iran gmziarani@hotmail.com gmohammadi@alzahra.ac.ir +98 21 88041344 +98 21 88041344

## Abstract

Indoles are some of the most versatile and common nitrogen-based heterocyclic scaffolds and are frequently used in the synthesis of various organic compounds. Indole based compounds are very important among heterocyclic structures due to their biological and pharmaceutical activities. The last decade, in particular, has witnessed considerable activity towards the synthesis of indole derivatives due to the possibilities for the design of polycyclic structures by the incorporation of multiple fused heterocyclic scaffolds in an attempt to achieve promising new heterocycles with chemical and biomedical relevance. In this study, we provide an overview on recent applications of indole in the multicomponent reactions for the synthesis of various heterocyclic compounds during the period of 2012 to 2017.

## Abstract 2

An overview on recent applications of indoles in multicomponent reactions for the synthesis of heterocyclic compounds is provided.

## Graphical abstract

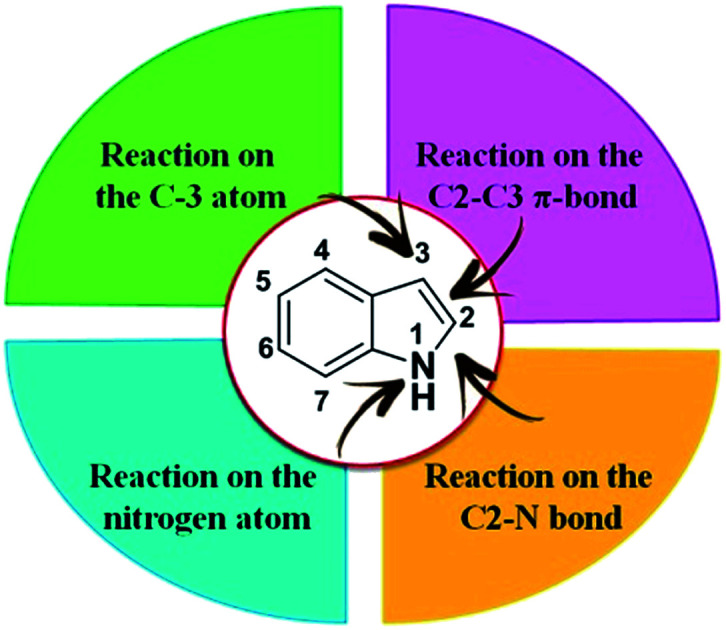

## 1. Introduction

Heterocyclic compounds are important tools in our daily life having an extensive variety of applications such as sanitizers,^1^ pharmaceuticals^2,3^ and antioxidant compounds,^4,5^ corrosion inhibitors,^6,7^ dye stuff,^8^ copolymers,^9,10^ and as building blocks in the synthesis of organic compounds and natural products. Multicomponent reactions (MCRs) have been extensively used for the synthesis of heterocyclic compounds.^11–13^ MCRs represent a great tool in organic synthesis for the construction of variety-oriented series of building blocks with potentially interesting biological activities.^14–17^ The attractiveness of the MCR approach is its easy operation, high selectivity and yield by using minimum synthetic requirements. Indole scaffolds have been known for their value in the development of new compounds of pharmaceutical interest.^18–20^ Up to date, several review articles have been published based on the reactions of indole.^21,22^ Herein, in continuation of our studies towards the synthesis of heterocyclic compounds and multicomponent reactions,^23–32^ and since there is a wide range of reactions that include indole in the preparation of heterocyclic compounds, this review presents the recent applications of indole in the synthesis of diverse heterocyclic compounds during the period from 2012 to 2017. This review first discusses indoles’ C-3 carbon atom reactivity applicable to electrophilic reactions, followed by MCRs in which the N position of indole is reacted as a nucleophile to afford N-substituted indole products. In Section 2.3, indole cycloaddition reactions have been discussed including cycloaddition reactions of the C2–C3 π-bond (Section 2.3.1) and the C–N sigma bond (Section 2.3.2). Finally, in Section 2.4, miscellaneous reactions of indole will be reviewed.

## 2. Multicomponent reactions of indoles

**Scheme 1:**
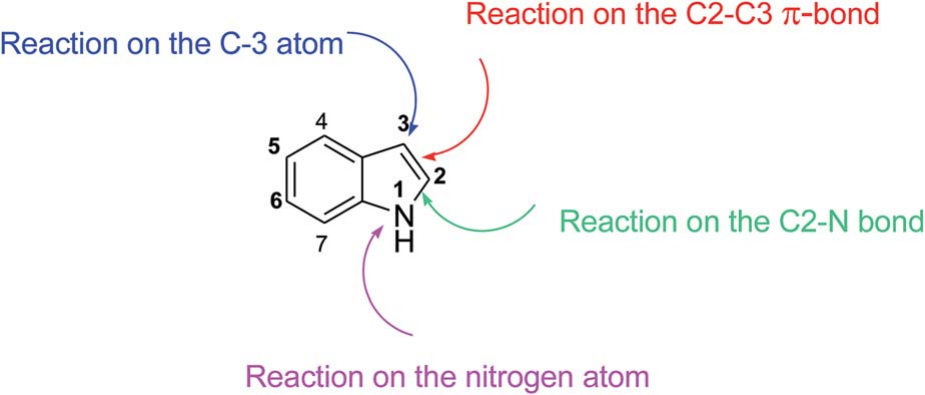


The indole structure is a heterocyclic compound which easily participates in chemical reactions. Its bonding sites are analogous to pyrrole. As shown in Scheme 1, indole is reactive at four different positions including the carbon atom 3, nitrogen atom 1, the C2–C3 π-bond and the C2–N sigma bond. Indole can be protonated with strong acids such as hydrochloric acid, which protonates the C3 position, more easily than the N atom. The cycloaddition reaction is another reaction of indole compounds. The C2–C3 π-bond of indole has a propensity towards cycloaddition reactions but cycloaddition reactions of the C2–N sigma bond are also observed.

### 2.1. The C-3 position reactions of indoles

**Scheme 2:**
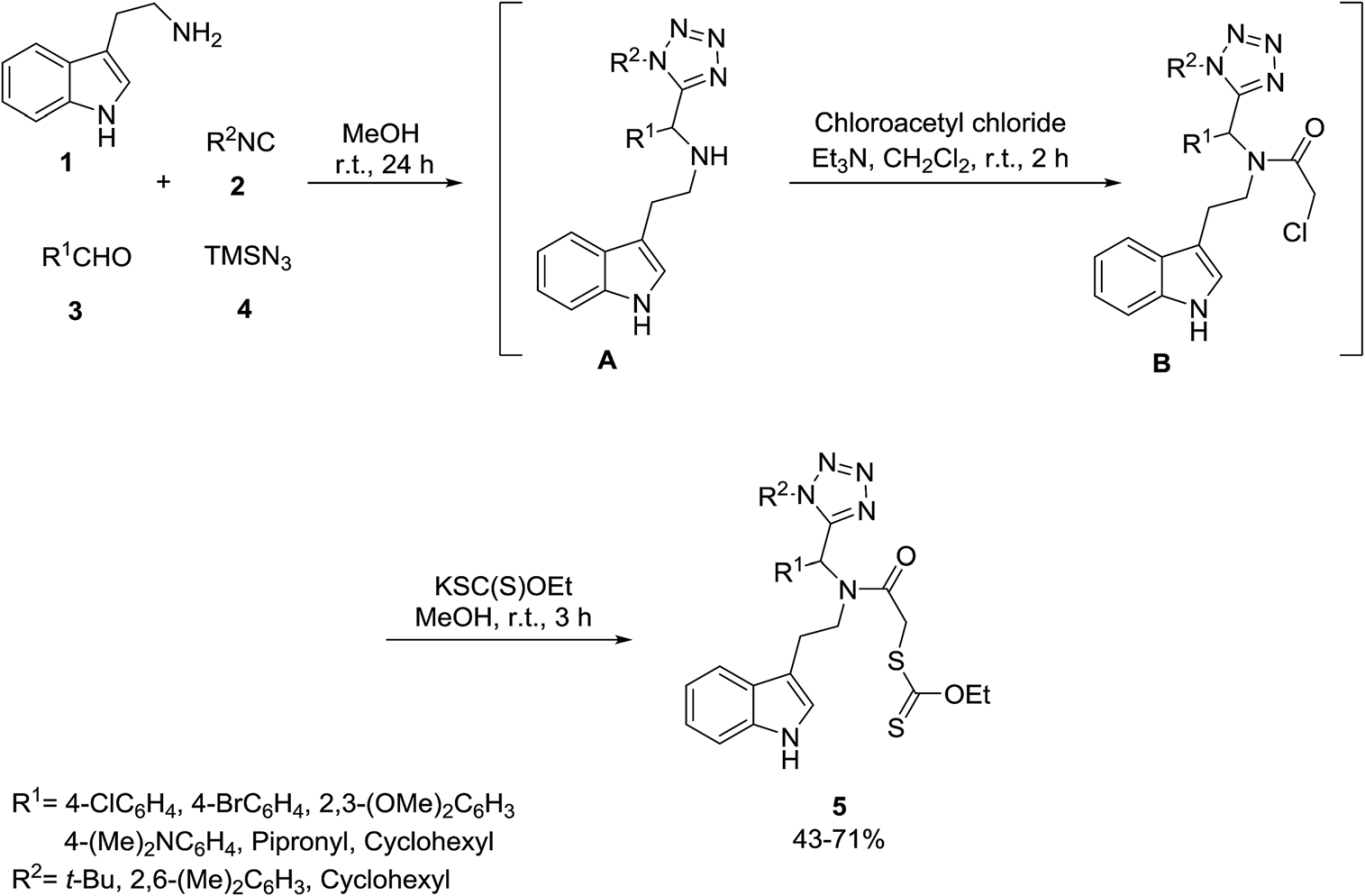


Gámez-Montaño’s group reported the one-pot Ugi-azide^33^ multicomponent reaction of indole 1, isocyanides 2, aldehydes 3 and TMSN_3_4 (Scheme 2). In the first step, intermediate A was obtained, and then *N*-acylation was performed between A and chloroacetyl chloride to give the intermediate B, which underwent an S_N_2 reaction with the potassium ethyl xanthogenate salt to give the final xanthates 5 (Scheme 2).^34^

**Scheme 3:**
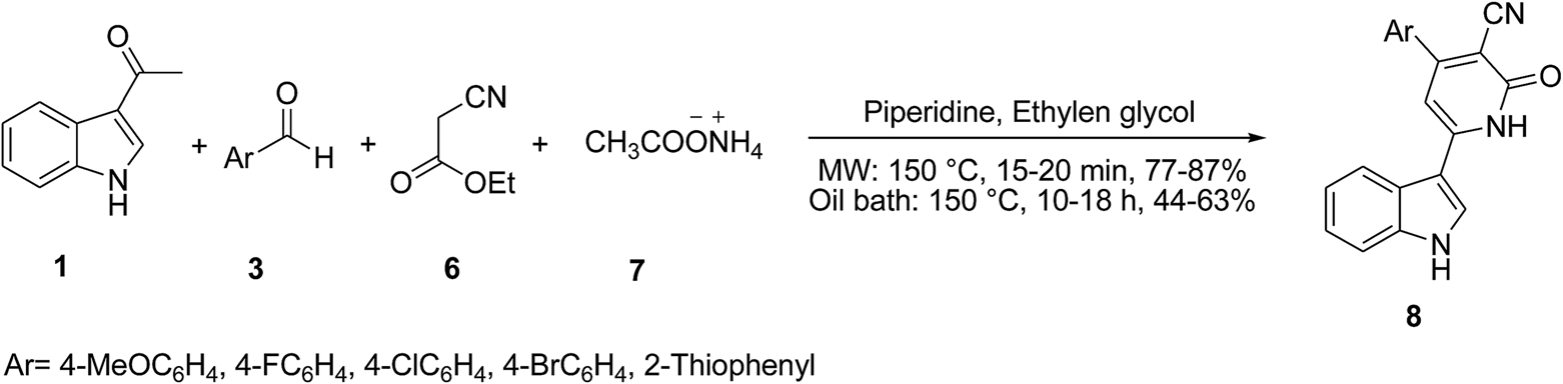


The one-pot multicomponent reaction of 3-acetylindole 1, aromatic aldehydes 3, ethyl cyanoacetate 6, and ammonium acetate 7 in the presence of piperidine as catalyst was established to access several 6-indolylpyridine-3-carbonitrile derivatives 8 (Scheme 3).^35^ The anti-proliferative activities of products were evaluated and showed good results.

**Scheme 4:**
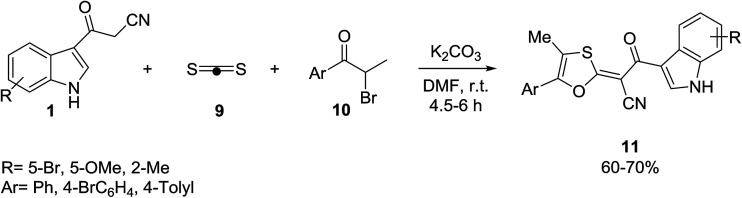


Indole 1, carbon disulfide 9 and substituted α-bromo propiophenones 10 were reacted *via* a three component domino [3 + 2] heterocyclization reaction for the preparation of two-carbon-tethered 1,3-oxathiole–indole pair compounds 11 (Scheme 4).^36^ The results showed that functional groups such as bromide and chloride provide ample opportunity for further functional group manipulations, for example, by modern cross-coupling reactions.

**Scheme 5:**
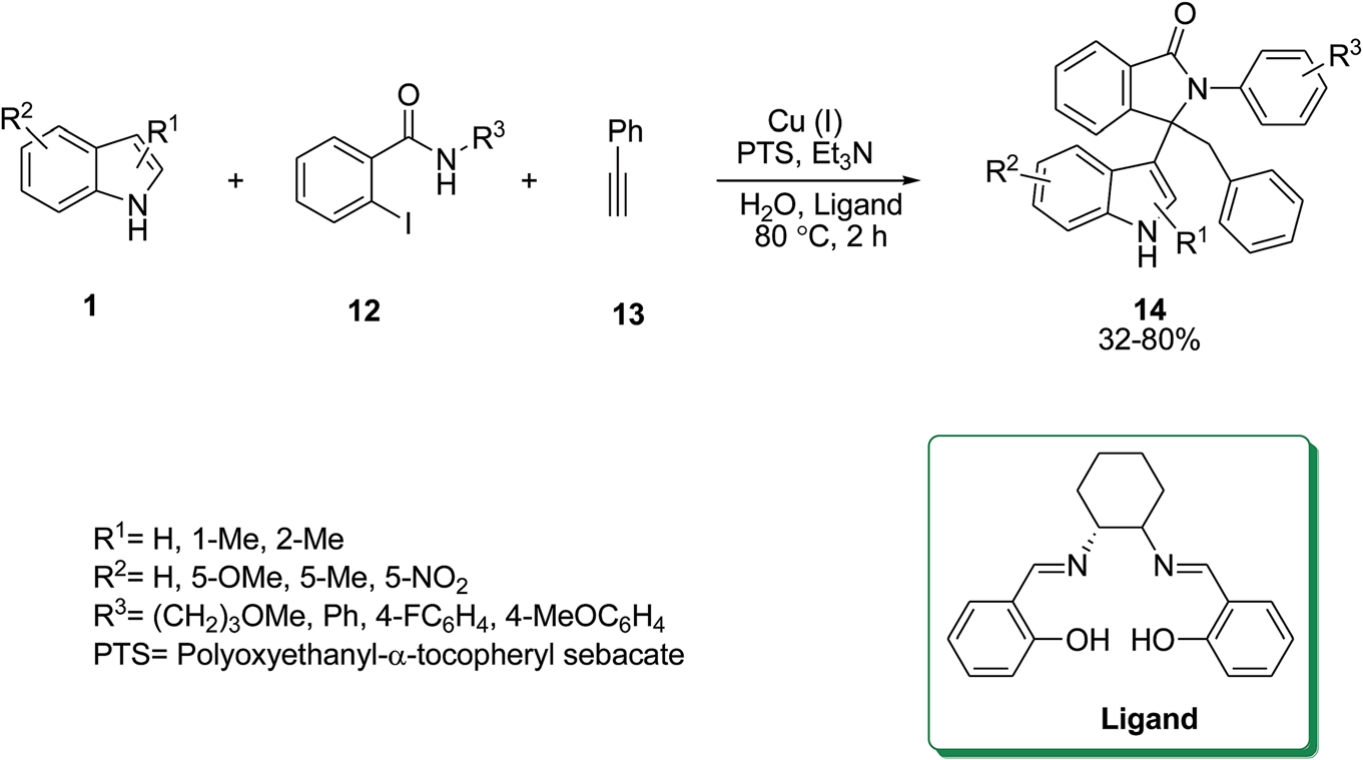


A regioselective Sonogashira^37^ cyclization reaction was carried out in the presence of CuI as catalyst and 2,2′-(1*E*,1′*E*)-(1*R*,2*R*)cyclohexane-1,2-diylbis(azan-1-yl-1-ylidene)bis(methan-1-yl-1-ylidene)diphenol as ligand to obtain benzyl-3-(indol-3-yl)-2-phenyl-2,3-dihydroisoindolinones 14. The reaction involved the one-pot multicomponent reaction of indoles 1, 2-iodo-*N*-phenylbenzamides 12 and terminal alkyne 13 under aerobic conditions followed by a nucleophilic addition (Scheme 5).^38^

**Scheme 6:**
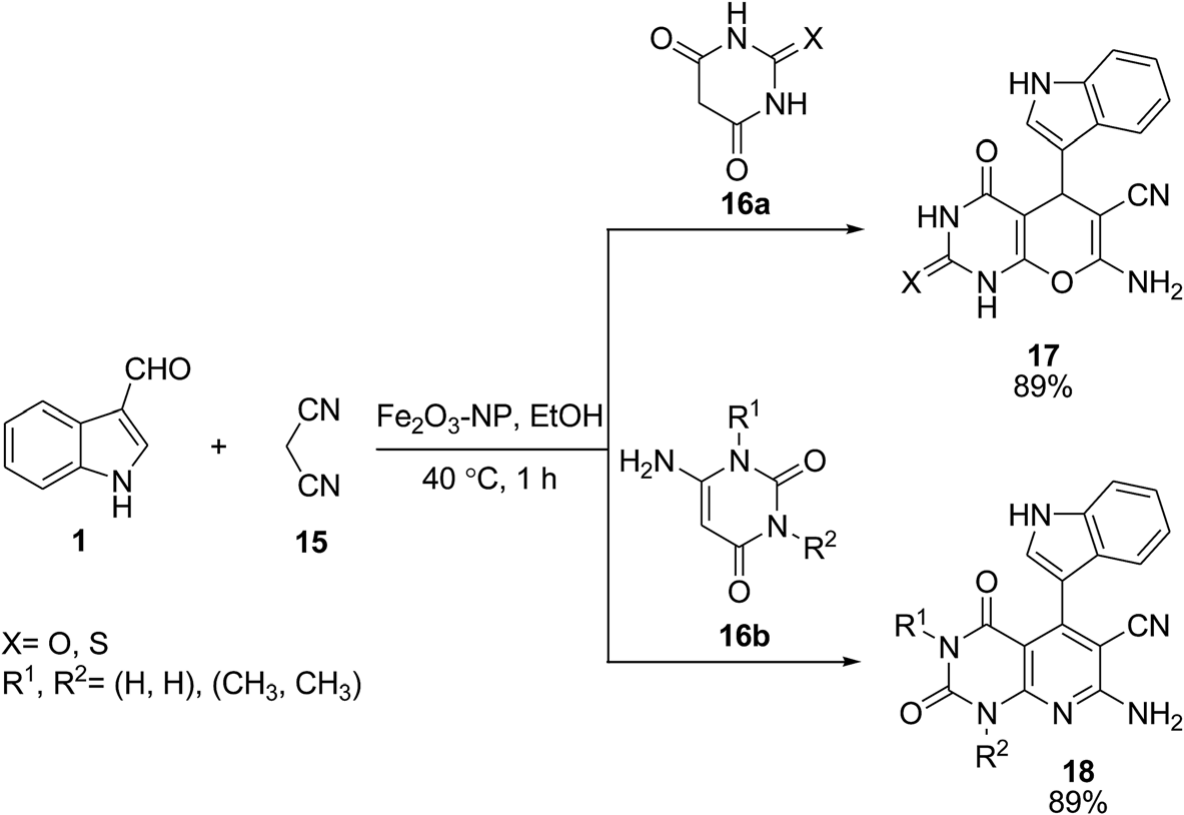


Magnetic nanoparticles (Fe_3_O_4_-NPs) catalyzed the synthesis of pyrano[2,3-*d*]pyrimidines 17 and pyrido[2,3-*d*]pyrimidines 18. The reactions were performed *via* the one-pot three component reaction of indole 1, malononitrile 15 and barbituric acids 16a or 6-amino uracil derivatives 16b in EtOH (Scheme 6).^39^

**Scheme 7:**
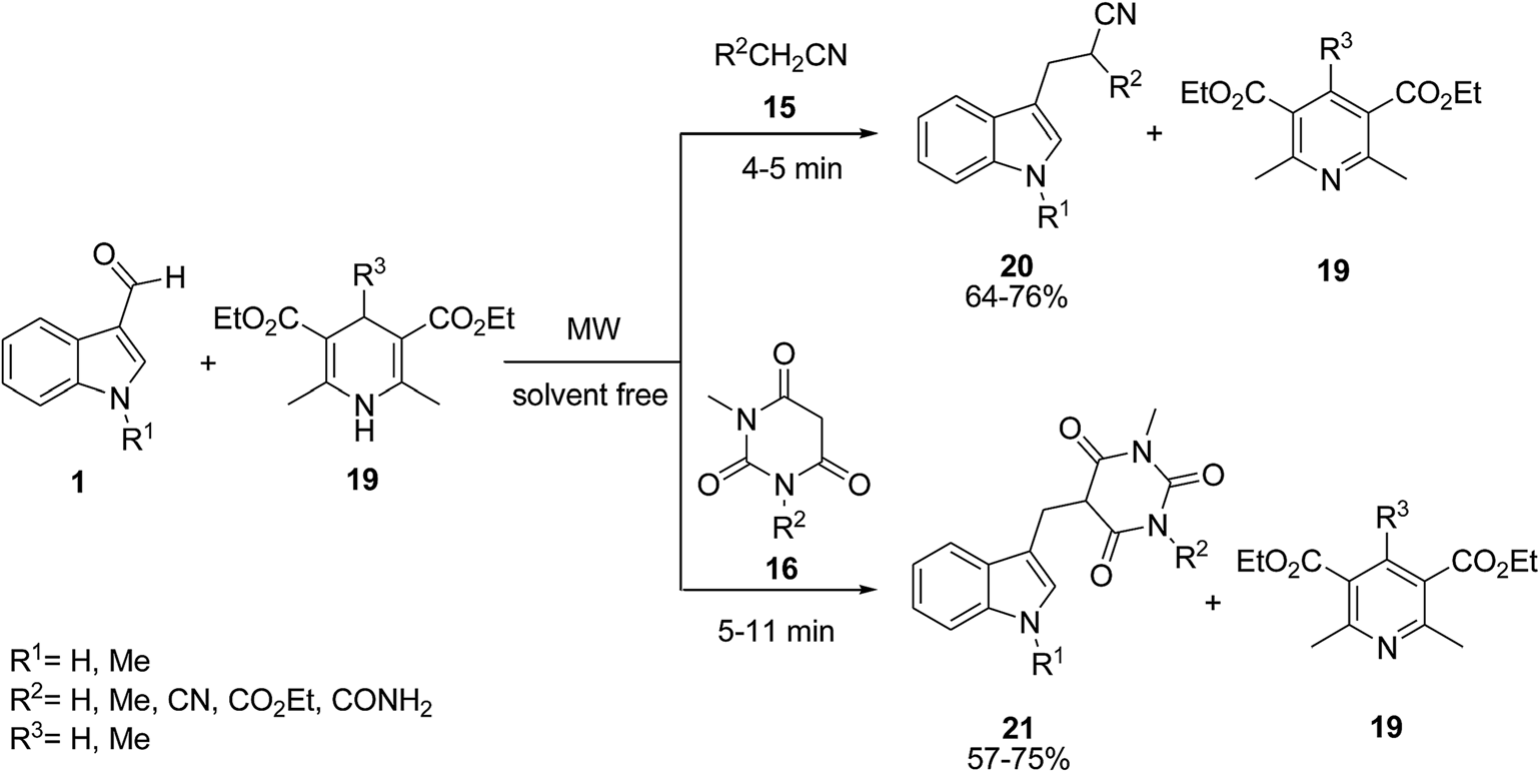


Baruah *et al.* presented the synthesis of 3-alkylated indole derivatives 20 and 21 under a microwave-assisted three-component reaction of indole-3-aldehydes 1, alkyl nitriles 15 or barbituric acids 16 with 1,4-dihydropyridine (DHP) derivative 19 (Scheme 7).^40^ In each case, DHPs are converted to pyridines. In fact, 1,4-dihydropyridine is the reducing agent.

**Scheme 8:**
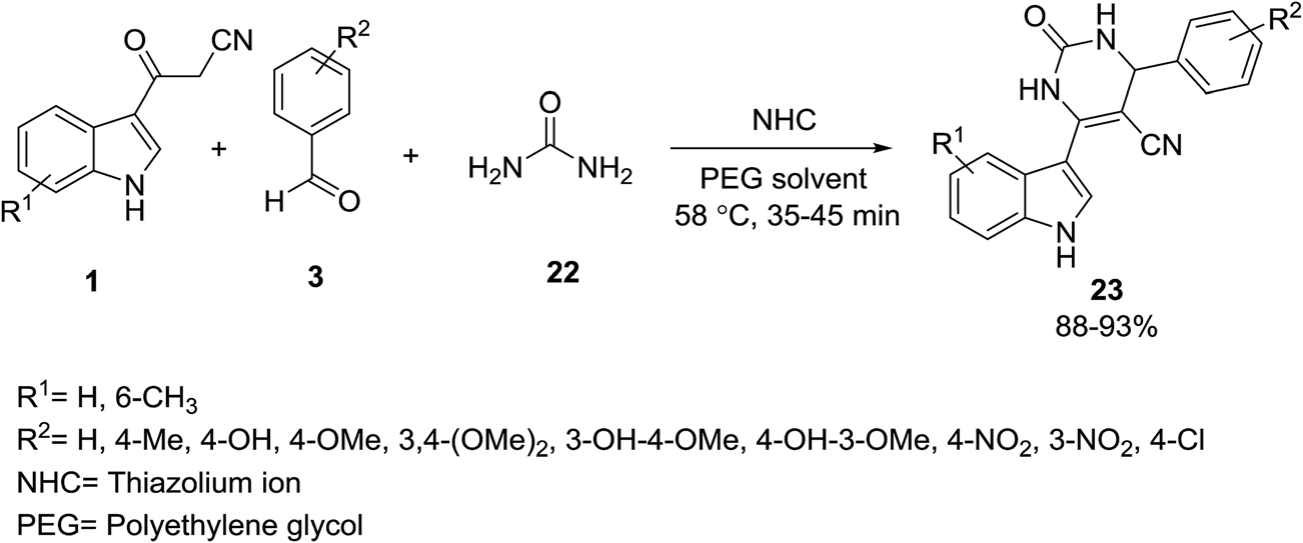


Preparation of a wide variety of new 6-(1*H*-indol-3-yl)-2-oxo-4-aryl-1,2,3,4 tetrahydropyrimidine-5-carbonitriles 23 was accessed by combining 3-(cyanoacetyl)-indoles 1 with an arylaldehyde 3 and urea 22 in the presence of PEG-400 and a catalytic amount of thiazolium anions (NHCs) (Scheme 8).^41^

**Scheme 9:**
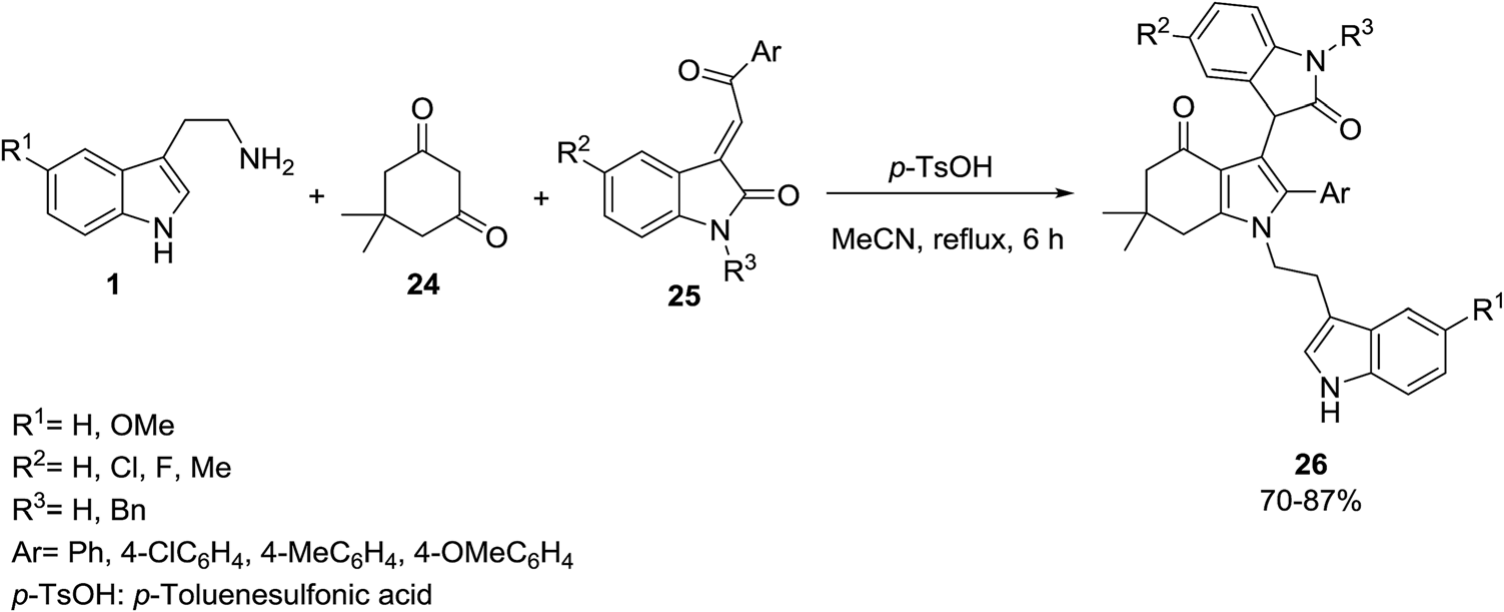


A three-component reaction for the synthesis of functionalized 3-{1-[2-(1*H*-indol-3-yl)ethyl]-4,5,6,7-tetrahydro-1*H*-indol-3-yl}indolin-2-ones 26 has been described by Jiang and Yan. The reaction involves a one-pot condensation of indole 1 with dimedone 24 and 3-phenacylideneoxindoles 25 in refluxing acetonitrile with *p*-toluenesulfonic acid as catalyst (Scheme 9).^42^

**Scheme 10:**
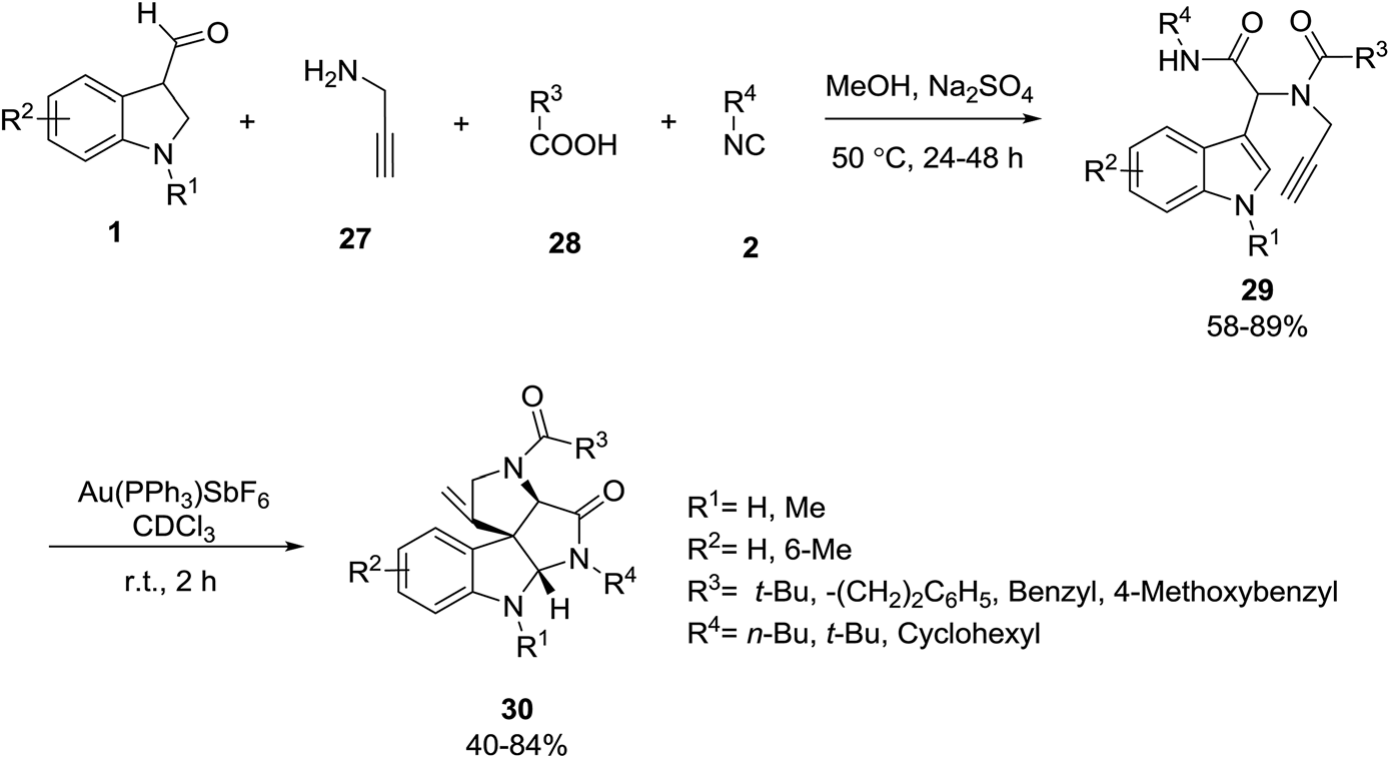


Modha and co-workers provided a novel procedure for the synthesis of diversely substituted spiroindolines 30*via* the post-Ugi gold-catalyzed diastereoselective domino cyclization. In this methodology, the Ugi reaction of indole-3-aldehydes 1, propargylamine 27, acids 28 and isocyanides 2 gave the products 29 in good yields which reacted with Au(PPh_3_)SbF_6_ in chloroform to produce spiroindolines 30 in moderate yields (Scheme 10).^43^ In another study, the same group used other amines instead of propargylamine for the synthesis of analogues of these products.^44^

**Scheme 11:**
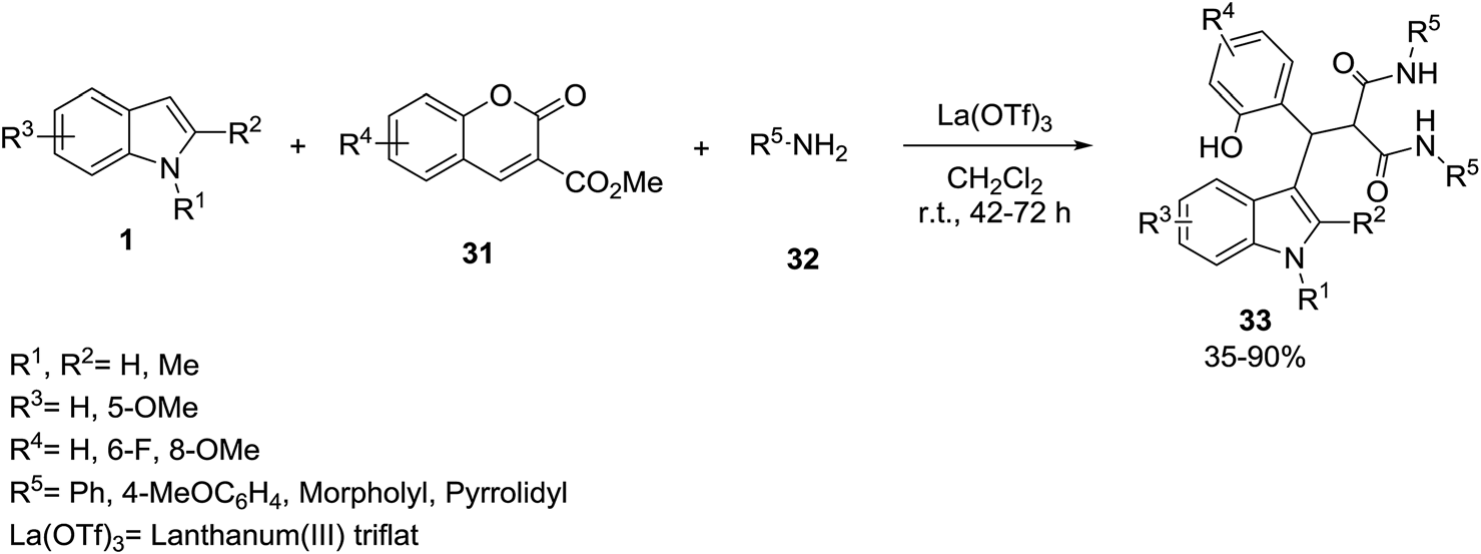


The indolylmalonamides 33 have been prepared *via* the three-component reaction of indole derivatives 1, chromene-3-carboxylates 31 and amines 32 in the presence of La(OTf)_3_ as a Lewis acid catalyst (Scheme 11).^45^ Indolylmalonamide products 33 showed notable fluorescence activities when they are exposed to long wave UV light (366 nm).

**Scheme 12:**
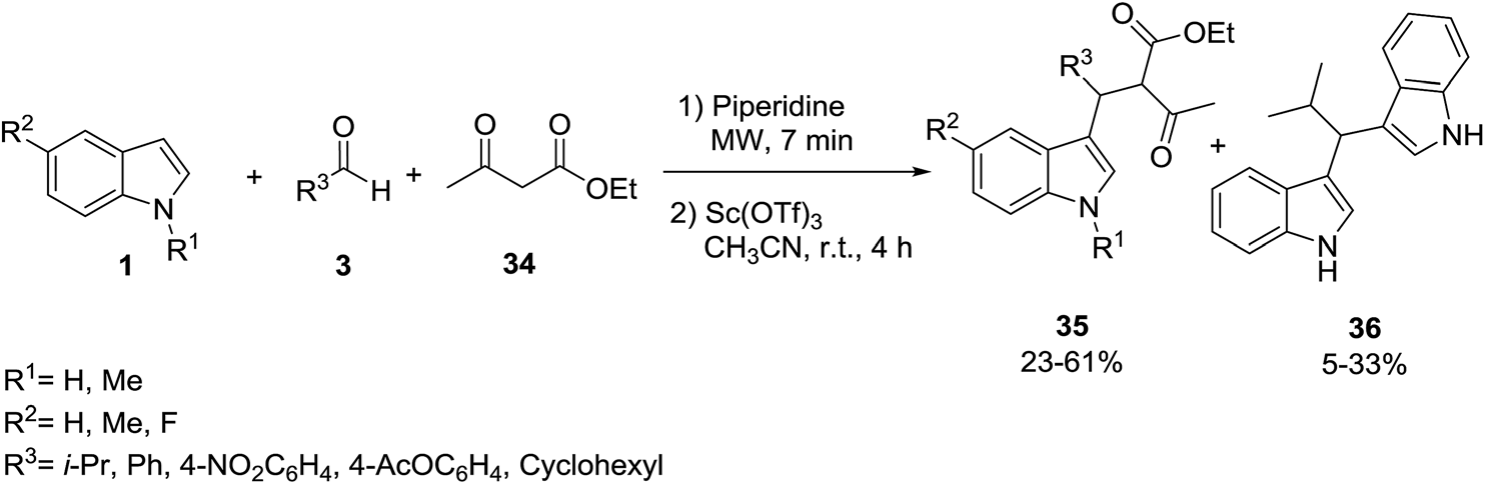


**Scheme 13:**
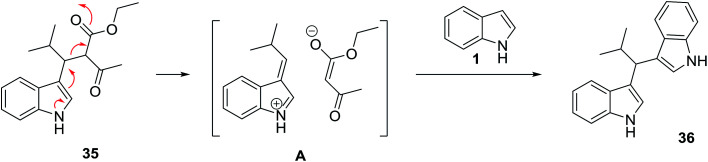


Polyfunctionalized indole derivatives 35 and 36 were generated from the Yonemitsu-type^46^ trimolecular condensation of indoles 1 with aldehydes 3 and 1,3-dicarbonyl compounds 34, such as malonates and acetoacetates using Lewis acid catalysts under microwave irradiation. The formation of bis-indolic derivative 36 can be easily rationalized in the one pot reaction, where a double addition of indole to the aldehyde is assumed. As already suggested by Gao and Wu,^47^ the adduct 35 is probably converted into a reactive indolenine derivative A^48^ by the loss of an active methylene fragment, which reacts with another molecule of indole (Schemes 12 and 13).^49^ Macroporous copper oxide (mpCuO) was also used as catalyst in this reaction and the same products were isolated in good yields.^50^ Docking studies against enoyl acyl carrier protein reductase predicted that the compounds bind at the active site with high binding affinity values. In another study, Li *et al.* used l-proline as catalyst in this reaction.^51^ The results are summarized in Table 1.

**Table 1:** Comparison of different conditions for the synthesis of products 35 and 36

Entry	Solvent	Catalyst	Temperature	Time	Yield (%)
1	CH_3_CN	Sc(OTf)_3_	r.t.	4 h	58–79 (ref. 49)
2	H_2_O	mpCuO	r.t.	15–31 min	78–95 (ref. 50)
3	EtOH	l-Proline	80 °C	0.25–4 h	70–99 (ref. 51)

**Scheme 14:**
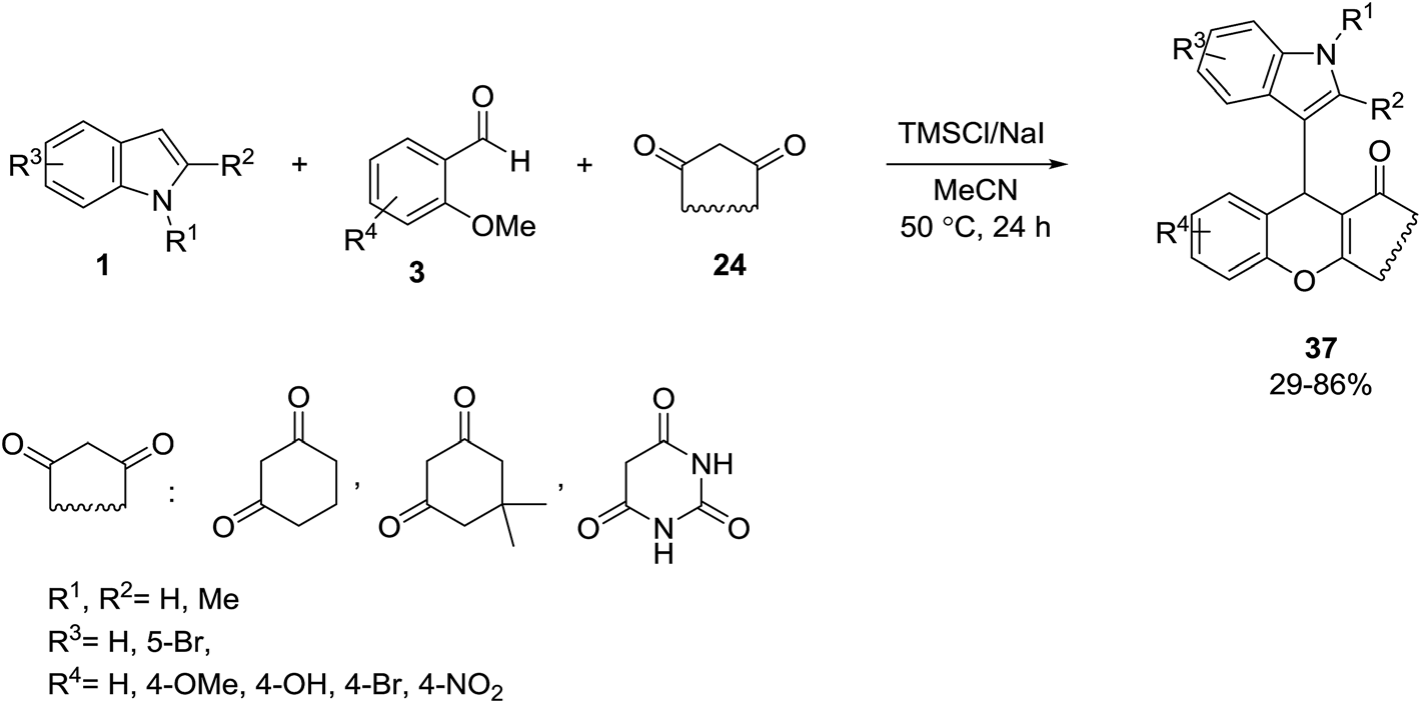


Khalafi-Nezhad *et al.* developed the use of trimethylsilyl iodide (TMSI) as a multifunctional agent in the one-pot synthesis of 9-(1*H*-indol-3-yl)xanthen-4-(9*H*)-ones 37 from the reaction of indoles 1, 2-methoxybenzaldehydes 3 (as *O*-methyl protected salicylaldehydes) and β-dicarbonyl compounds 24 (Scheme 14).^52^

**Scheme 15:**
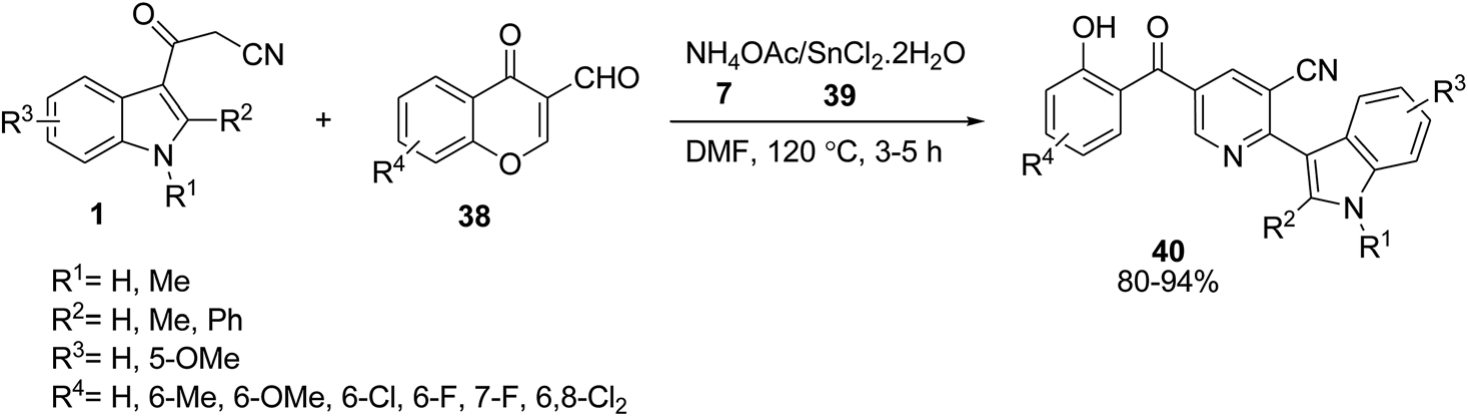


The functionalized indole-3-yl pyridines 40 were prepared *via* an efficient one-pot condensation of cyanoacetylindoles 1, 3-formylchromones 38 and ammonium acetate 7 under stannous chloride 39 mediation in DMF (Scheme 15).^53^

**Scheme 16:**
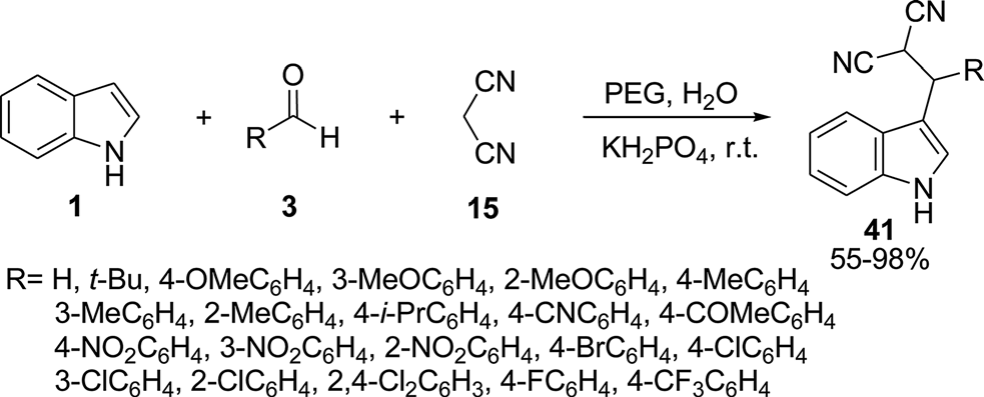


Wan and co-workers used polyethylene glycol (PEG-200) in a three-component reaction of indoles 1, aldehydes 3, and malononitrile 15 to afford 3-indole derivatives 41 in good to excellent yields (Scheme 16).^54^l-Proline,^55^ tetrabutylammonium fluoride (TBAF),^56^ Zn-salphen,^57^ Cu(OAc)_2_ (ref. 58) and Cu(iii)^59^ were also used as catalysts in this reaction and the results are shown in Table 2.

**Table 2:** Comparison of different conditions for the synthesis of products 41

Entry	Solvent	Catalyst	Temperature (°C)	Time (h)	Yield (%)
1	H_2_O	PEG-200	r.t.	3	55–98 (ref. 54)
2	EtOH	l-Proline	r.t.	30–72	58–98 (ref. 55)
3	—	TBAF·3H_2_O	60	2	55–97 (ref. 56)
4	DCM	Zn-salphen, DIPEA	r.t.	6	13–60 (ref. 57)
5	PEG-400	Cu(OAc)_2_	70	15–40	48–98 (ref. 58)
6	H_2_O	Cu(iii)	30	12–24	70–96 (ref. 59)

**Scheme 17:**
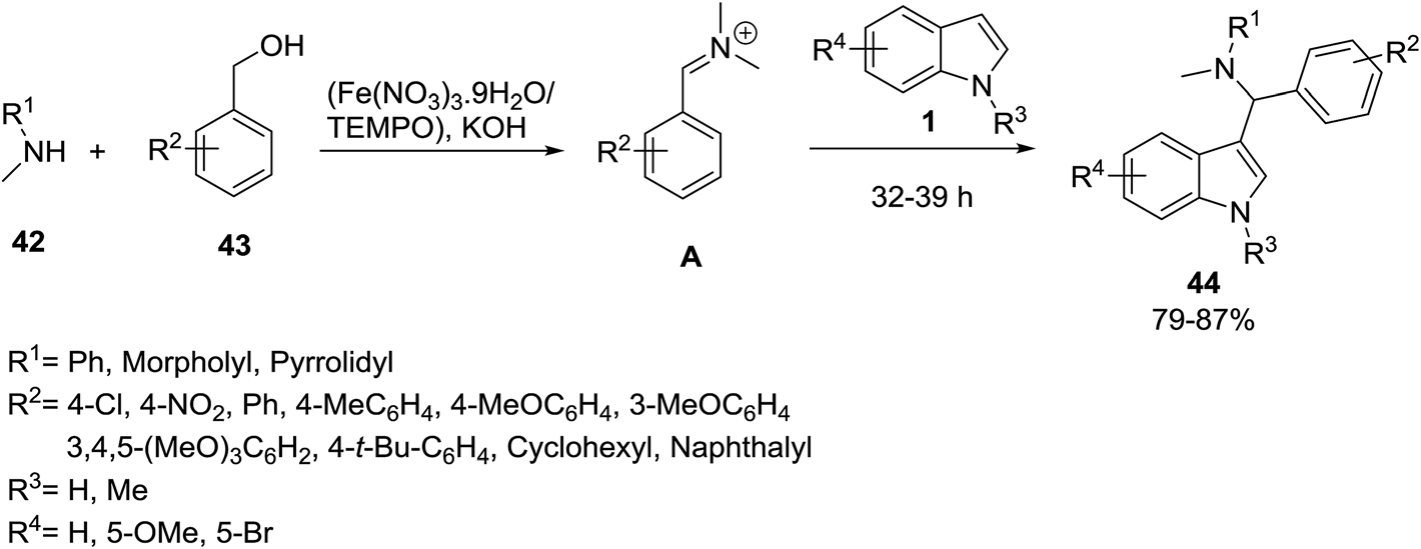


Singh’s group described a highly efficient methodology for the synthesis of 3-amino-alkylated indoles 44*via* the one-pot three-component Mannich^60^ type reaction of amines 42, alcohols 43 and indoles 1. Treatment of amines 42 and alcohols 43 with KOH in toluene in the presence of (Fe(NO_3_)_3_·9H_2_O/TEMPO)^61^ as catalyst, yielded iminium ion A. Then, the iminium ion A was reacted with indoles 1 to obtain novel 3-substituted indoles 44 (Scheme 17).^62^ In another study, ferric hydrogen sulfate (FHS)^63^ was applied as catalyst and the same products were prepared in 87–98%. It was observed that electron-withdrawing groups on the aldehyde reacted rapidly and were better reagents in this reaction. Furthermore, *N*-methylaniline showed better reactivity in comparison with *N*-ethylaniline due to the low steric effects. *N*-Alkylanilines were used in excess to avoid the formation of bis(indolyl)alkanes.^64^ The reaction was also catalyzed by l-proline,^65^ Amberlite, IRA-400 Cl resin^66^ and polyaniline-fluoroboric acid-dodecyl hydrogen sulfate salt (PANI-HBF_4_).^67^ Catalyst-free conditions in MeOH have also been reported in 72 h by 28–99% yields.^68^ A comparison of different catalysts and experimental setups is given in Table 3.

**Table 3:** Comparison of different conditions for the synthesis of product 44

Entry	Solvent	Catalyst	Temperature (°C)	Time (h)	Yield (%)
1	Toluene	(Fe(NO_3_)_3_·9H_2_O/TEMPO)	r.t.	32–39	79–87 (ref. 62)
2	—	FHS	45	1–4	87–98 (ref. 63)
3	—	l-Proline	r.t.	5–18	68–89 (ref. 65)
4	MeOH	IRA-400 Cl resin	r.t.	1.5–3	70–85 (ref. 66)
5	H_2_O	PANI-HBF_4_	r.t.	30–50 min	88–97 (ref. 67)
6	MeOH	—	30	72	28–99 (ref. 68)

**Scheme 18:**
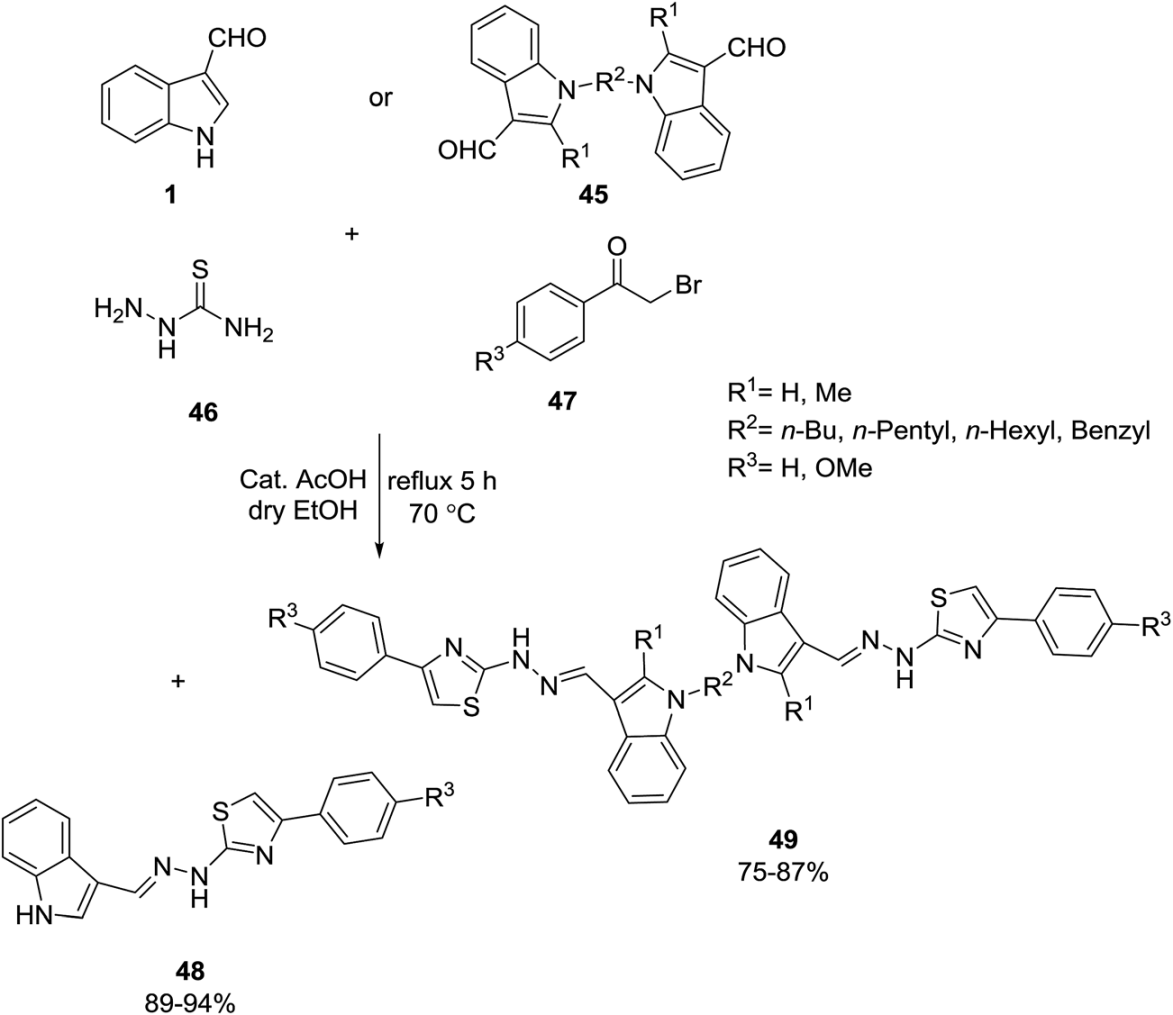


Mahmoodi and co-workers developed the one-pot cyclocondensation of mono- or bis(indole-3-carbaldehyde) 1 or 45,^69,70^ thiosemicarbazide 46, and phenacyl bromides 47 in the presence of a catalytic amount of AcOH for the preparation of the novel mono- and bis(indol-3-yl)hydrazineyl thiazole derivatives 48 and 49 (Scheme 18).^71^ The products were evaluated for *in vitro* antibacterial activity against Gram-positive and Gram-negative bacteria. Some of the products have good antibacterial activity. The product 48 with OCH_3_ as a donating group exhibited high activity against Gram-positive bacteria.

**Scheme 19:**
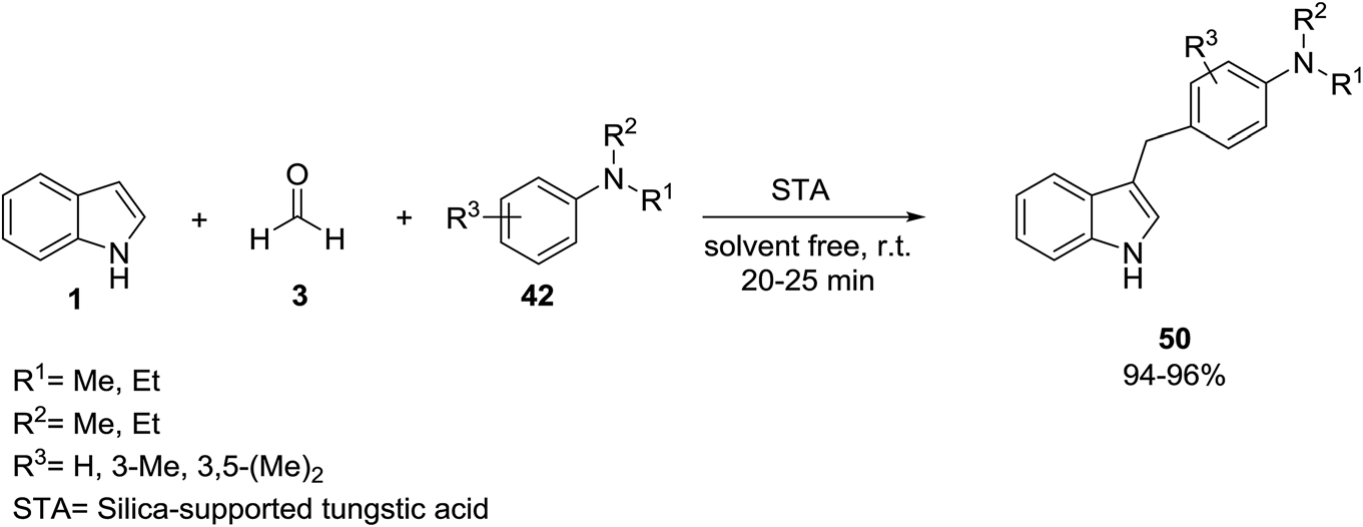


Shinde and Jeong developed the reaction between indole 1 and formaldehyde 3 with tertiary aromatic amines 42 in the presence of silica-supported tungstic acid (STA) as a heterogeneous acid catalyst under solvent-free conditions. The protocol was performed *via* the three component Mannich type Friedel–Crafts addition for the preparation of *N*,*N*-dialkyl amino arylated indole derivatives 50 (Scheme 19).^72^ Application of sodium dodecyl sulfate (SDS) as surfactant in this reaction was also reported by Kumar *et al.* resulting in 78–94% yields of products.^73^

**Scheme 20:**
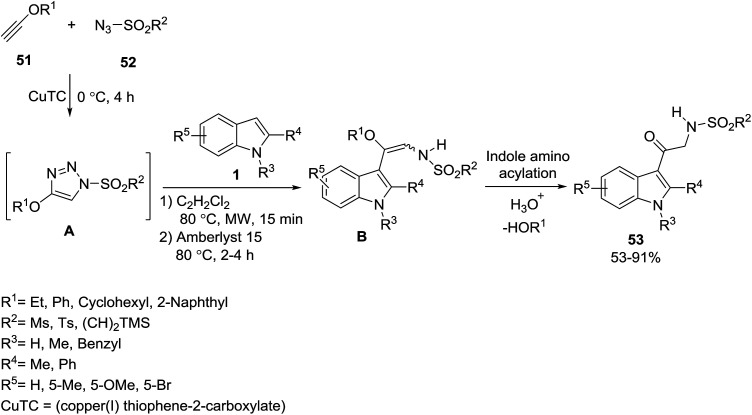


Mild aminoacylation of indoles 1 through a multicomponent process with ynol ethers 51 and sulfonyl azides 52 was established by Alford and Davies for the synthesis of oxo-tryptamines 53 (Scheme 20).^74^ First, 4-alkoxy *N*-sulfonyltriazoles A^75^ were generated from ynol ethers 51 and sulfonyl azides 52, and treated with indoles 1. Then, the obtained enol ethers B were converted to the amino ketones 53 for the α-aminoacylation of enols.^76^

**Scheme 21:**
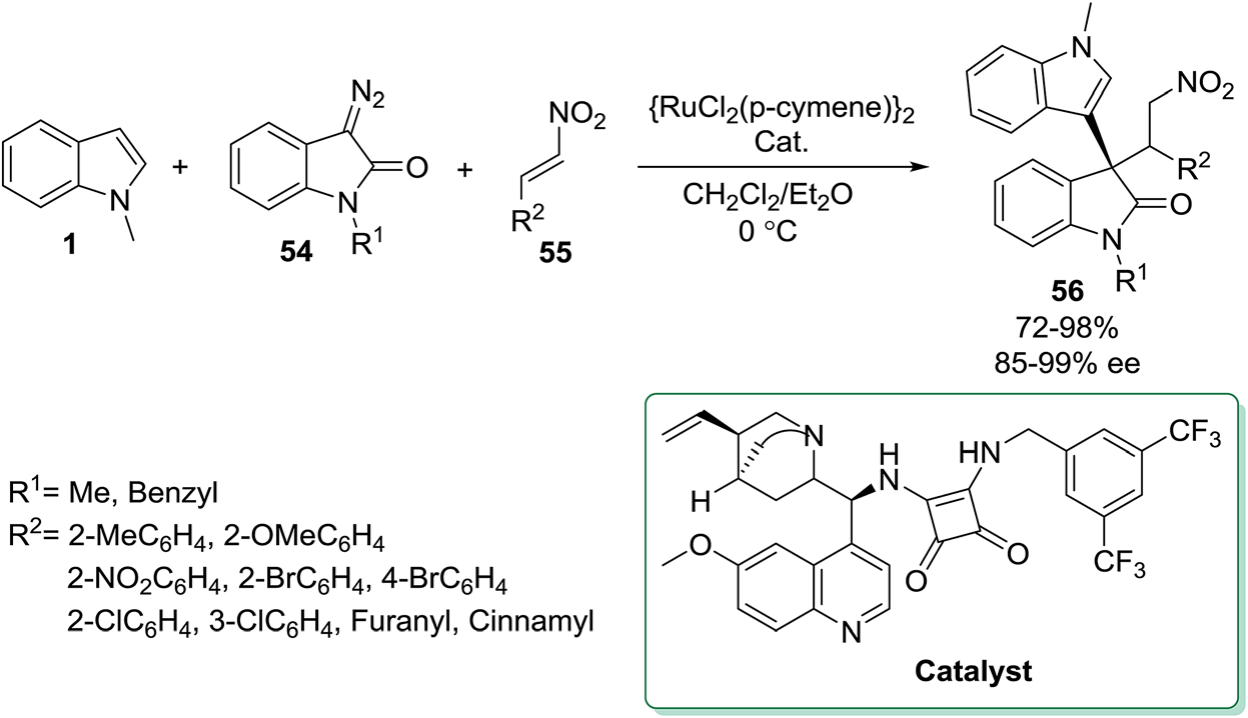


*N*-Methyl indole 1 was reacted with diazooxindole 54 and nitrostyrene 55 in the presence of [Ru] and squaramide as catalysts *via* an asymmetric Michael addition^77^ for the synthesis of 3,3′-bis(indole) derivatives 56 (Scheme 21).^78^

**Scheme 22:**
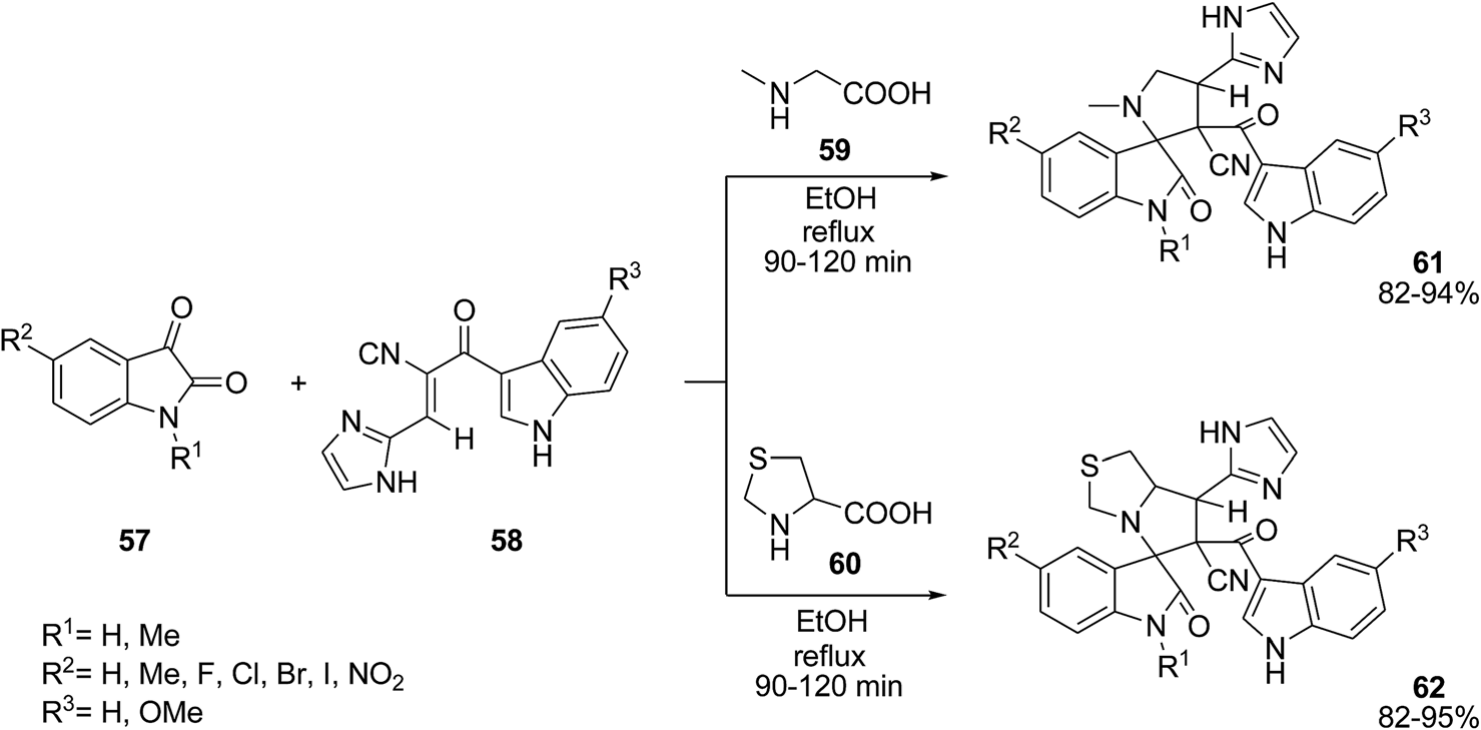


Novel spirooxindole-pyrrolidine compounds 61 and 62 were obtained through 1,3-dipolar cycloaddition of azomethine ylides generated from isatin 57 and sarcosine 59 or thioproline 60 with the dipolarophile 3-(1*H*-imidazol-2-yl)-2-(1*H*-indole-3-carbonyl)acrylonitrile 58 (Scheme 22).^79^ Anticancer activity studies were carried out for the synthesized compounds against A549 lung adenocarcinoma cancer cell line.^80^ Several of the products showed very high activity against the cancer cell line. Reddy and co-workers also studied this reaction in MeOH as solvent under reflux conditions in 2–3 h giving 80–93% yields. The antimicrobial activity of all products were evaluated against several bacteria and fungi, and showed good activity.^81^

**Scheme 23:**
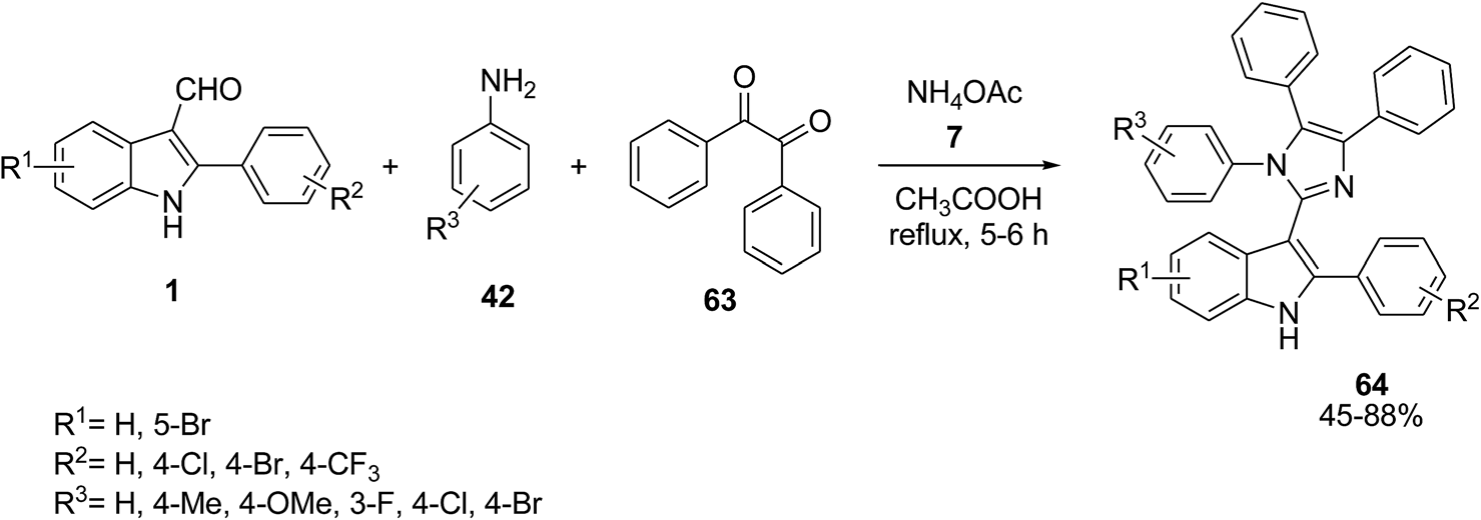


A one-pot four-component condensation strategy was employed by Naureen’s group for the discovery of indole-based tetra-arylimidazoles 64. This method involves the reaction of 2-arylindole-3-carbaldehydes 1, substituted anilines 42, benzil 63 and ammonium acetate 7 in acetic acid (Scheme 23).^82^ The anti-urease activity of the synthesized compounds was evaluated and showed good results.

**Scheme 24:**
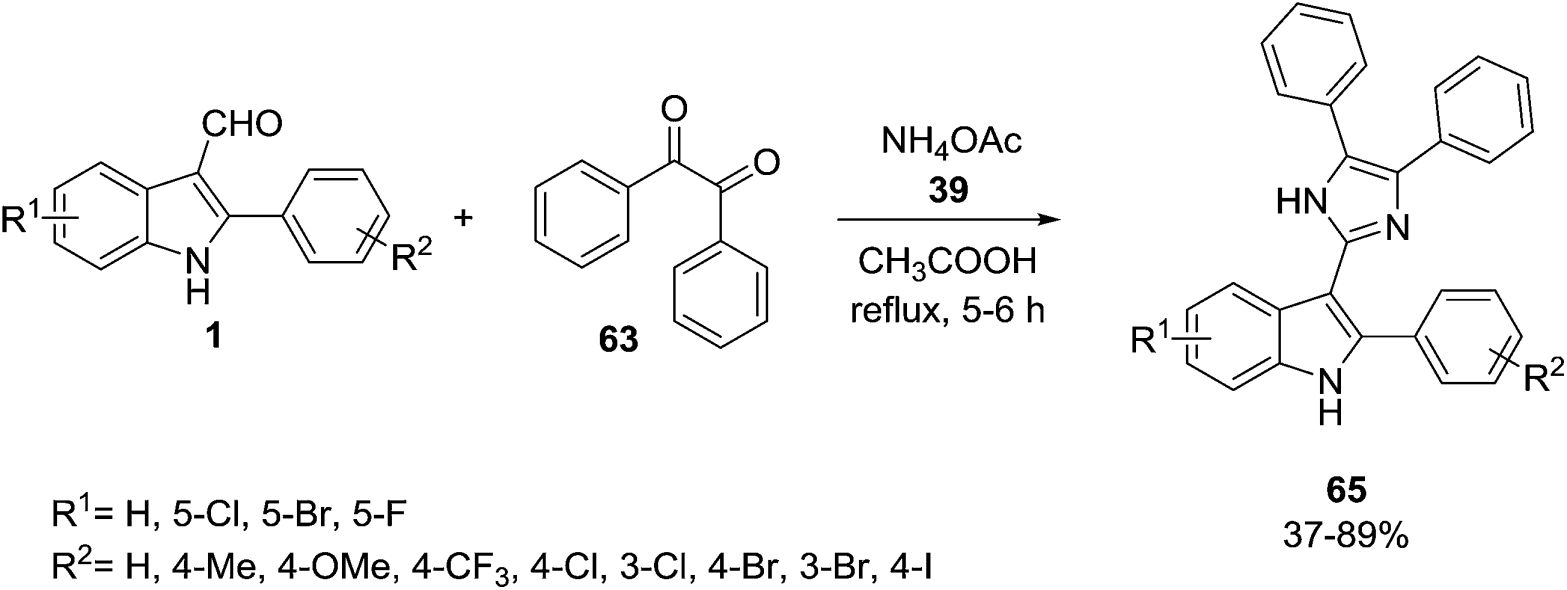


The same authors synthesized several new trisubstituted imidazoles, 3-(4,5-diaryl-1*H*-imidazol-2-yl)-2-phenyl-1*H*-indoles 65, *via* the condensation of substituted indole aldehydes 1, benzil 63 and ammonium acetate 7 in refluxing acetic acid (Scheme 24).^83^ The products were evaluated for their α-glucosidase inhibition and showed significant α-glucosidase inhibitory activity.

**Scheme 25:**
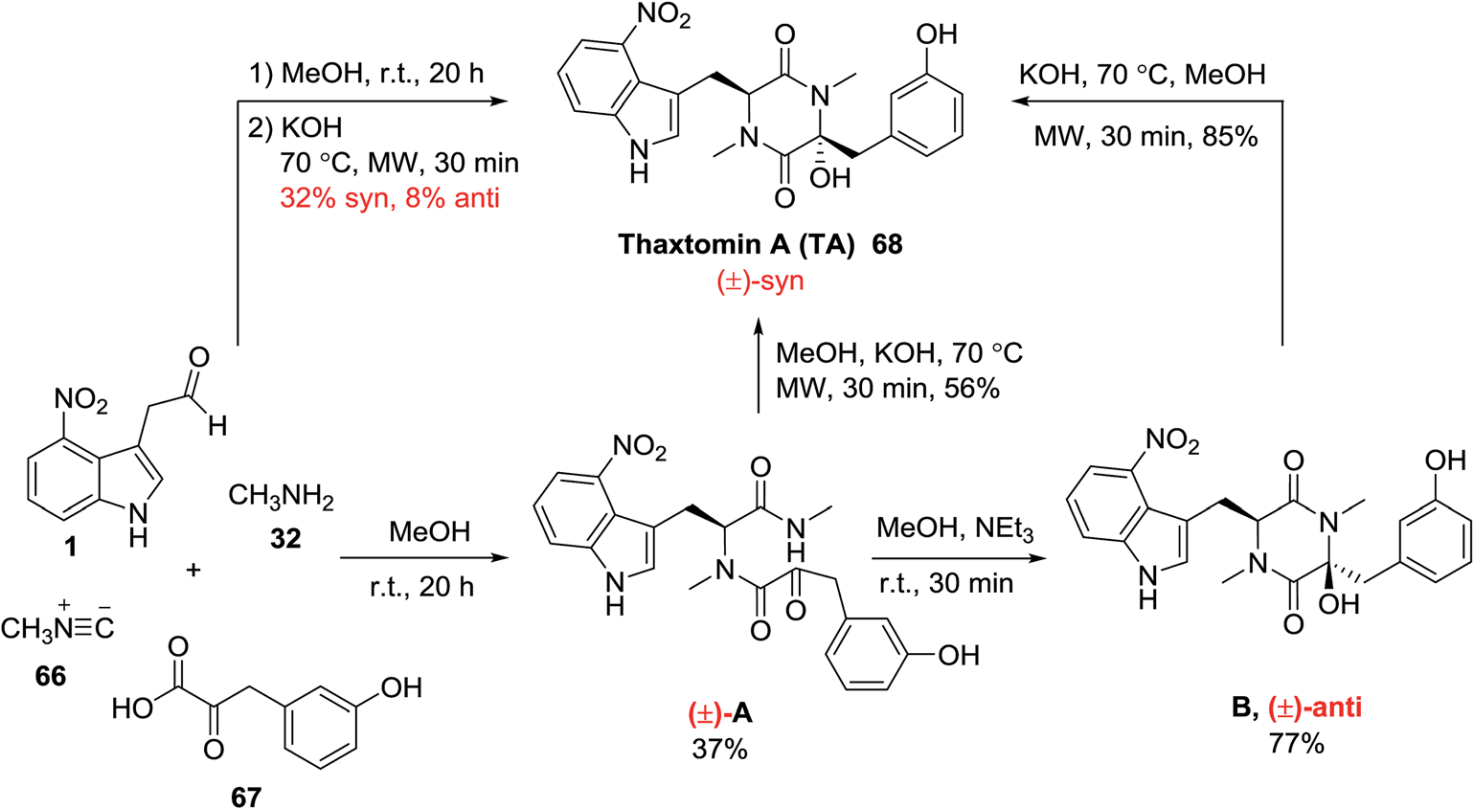


Andreana and his group utilized the one-pot reaction of 4-nitroindolylacetaldehyde 1, methylamine 32, methyl isocyanide 66 and 3-hydroxyphenylpyruvic acid 67 for the synthesis of (±)-thaxtomin A (TA) 68 as a herbicidal natural product. First, the prerequisite dipeptide A was isolated which through a base-mediated keto-amide cyclization reaction afforded two diastereomeric compounds B. Then, compound B was treated with KOH under microwave irradiation to provide the intended product 68 (Scheme 25).^84^ This natural product was synthesized previously by Zhang *et al.* and has been demonstrated to possess activity against the tobacco mosaic virus.^85^

**Scheme 26:**
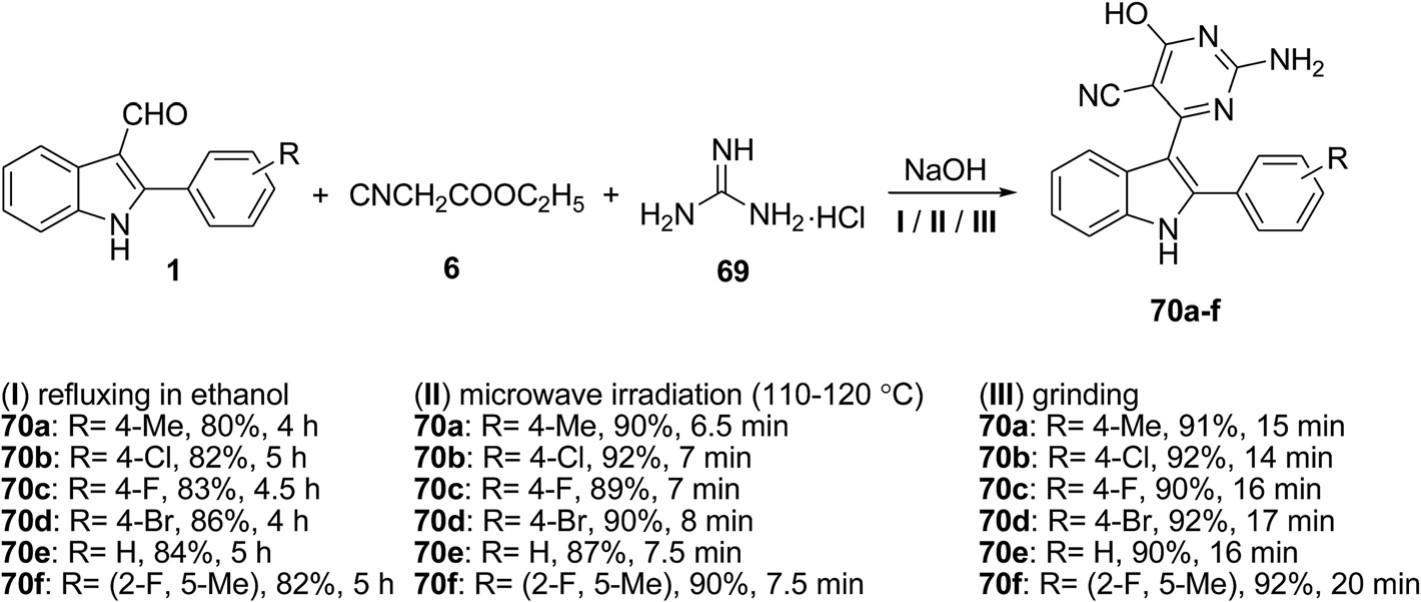


A one-pot three-component reaction of indole-3-aldehyde derivatives 1, ethyl cyanoacetate 6, and guanidine hydrochloride 69 under three different conditions, including microwave irradiation, grindstone technology and reflux, was developed to afford 2-amino-5-cyano-4-[(2-aryl)-1*H*-indol-3-yl]-6-hydroxypyrimidines 70 (Scheme 26)^86^ The products 70 were evaluated for their antimicrobial activity against nine pathogenic bacteria and showed mild to moderate activity.

**Scheme 27:**
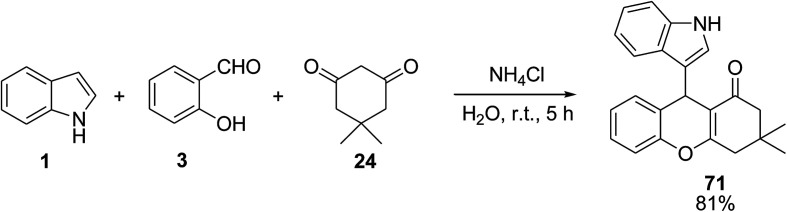


Bhattacharjee *et al.* employed ammonium chloride (NH_4_Cl) as catalyst for the preparation of 9-(1*H*-indol-3-yl)-3,3-dimethyl-2,3,4,9-tetrahydro-1*H*-xanthen-1-one 71*via* the one-pot three-component reaction of indole 1 with salicylaldehyde 3 and dimedone 24 (Scheme 27).^87^ In another study, Ganguly *et al.* developed this reaction in the presence of l-proline as catalyst in H_2_O for 2 h in 86–96% yield.^88^

**Scheme 28:**
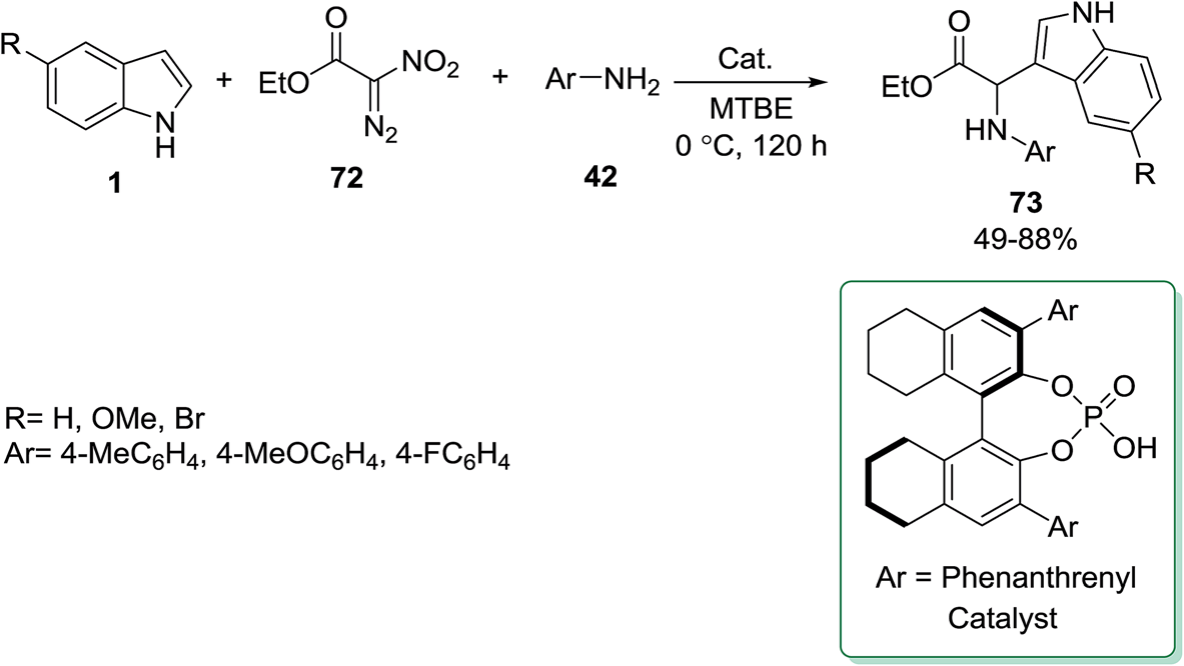


So and Mattson reported on the synthesis of glycine products 73 in the presence of chiral BINOL-based phosphoric-acid as catalyst. The process involves the multicomponent coupling reactions of indole derivatives 1, nitrodiazoester 72 and anilines 42 in methyl *t*-butyl ether (MTBE) as solvent (Scheme 28).^89^

**Scheme 29:**
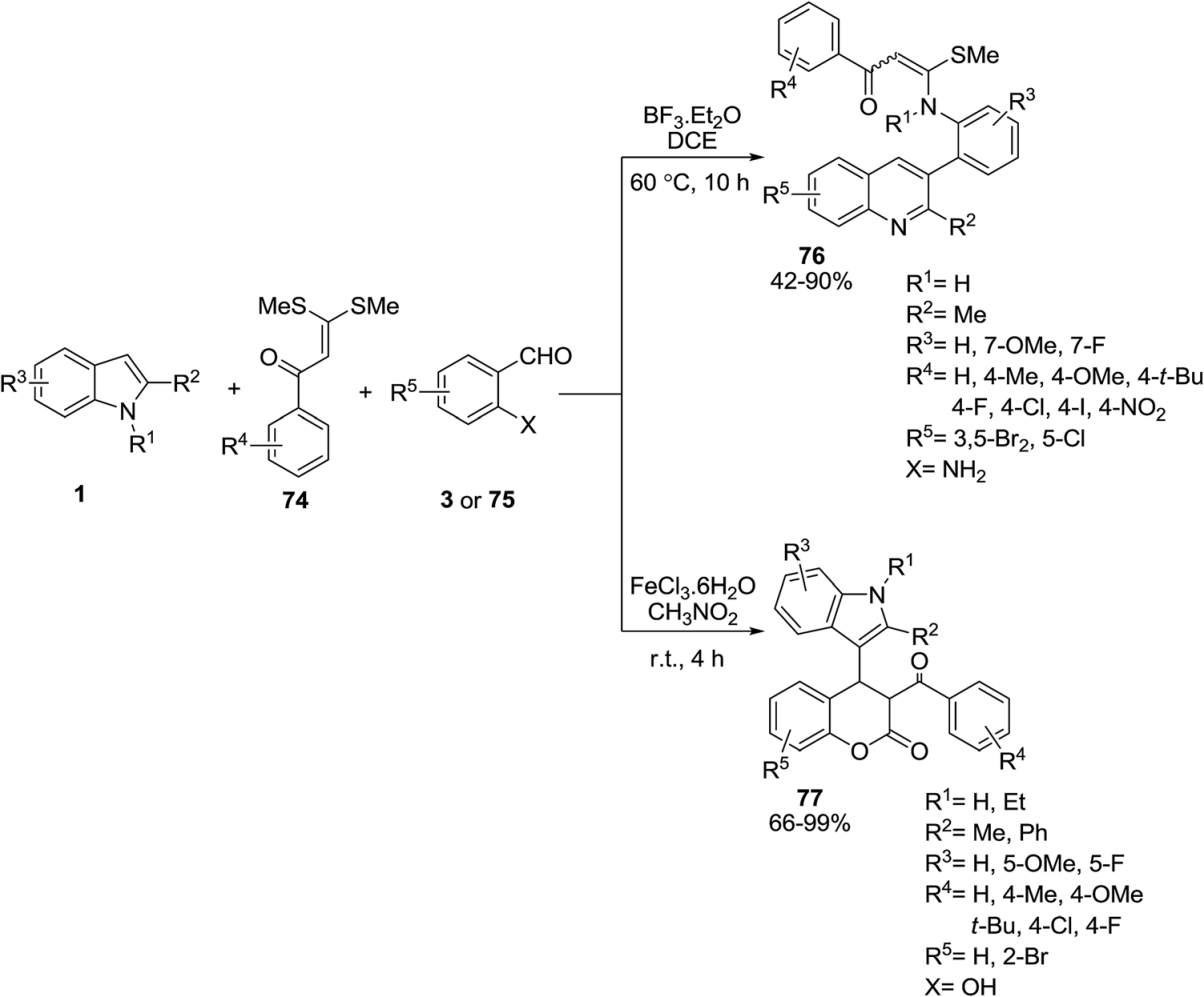


**Scheme 30:**
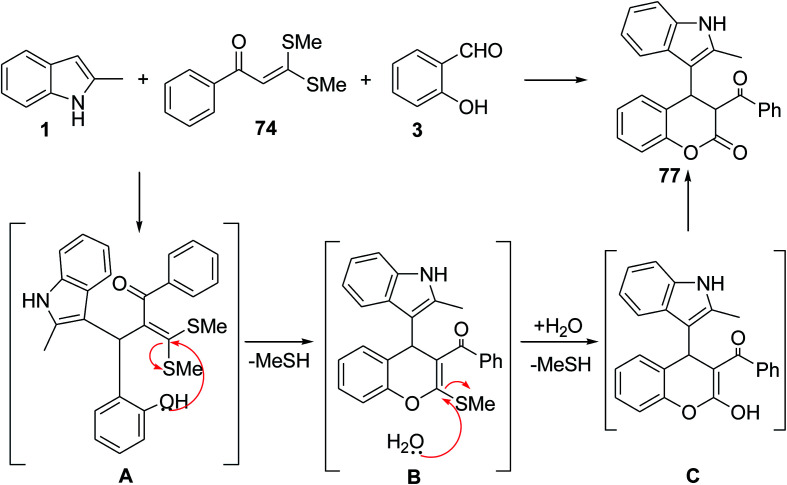


**Scheme 31:**
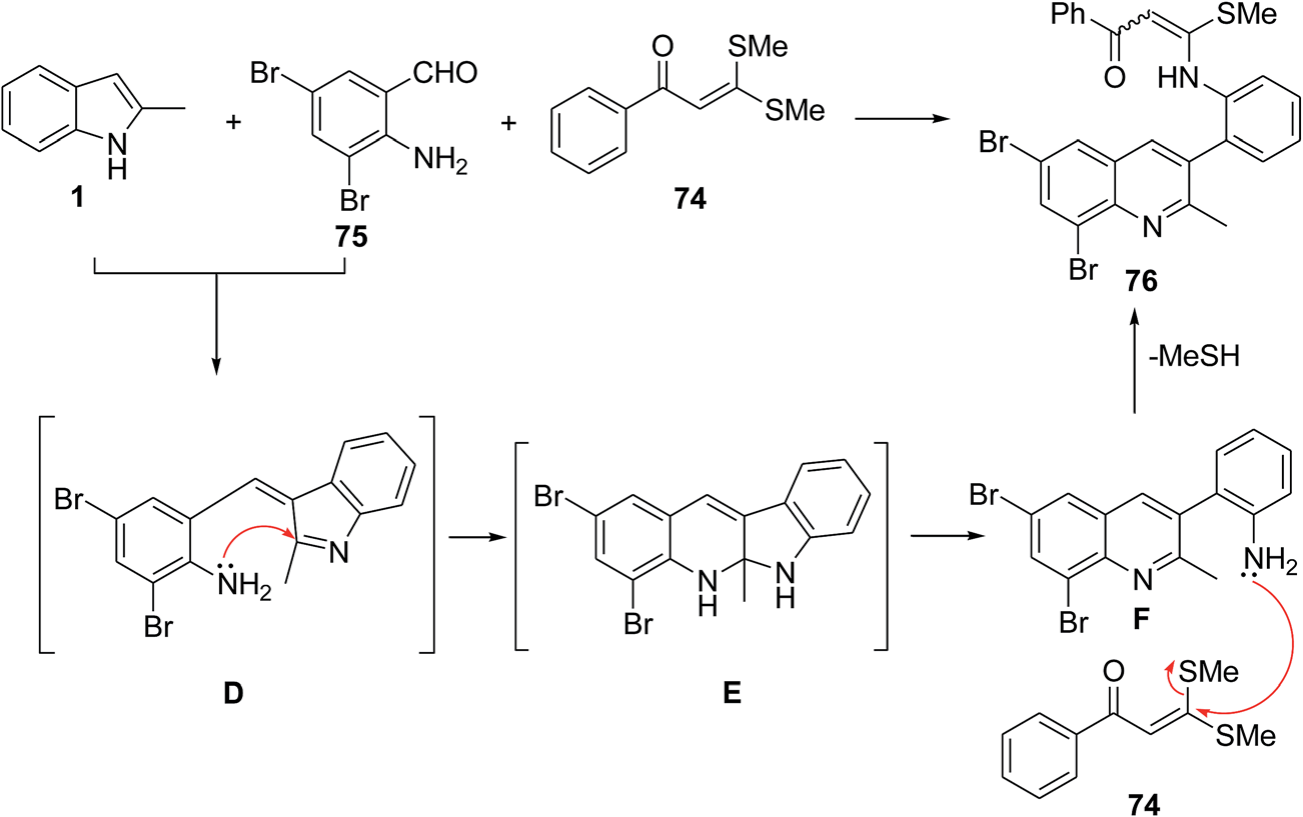


The three-component reaction of indoles 1, α-oxoketene dithioacetals 74, and aldehyde 3 or 75 was investigated for the synthesis of dihydrocoumarins 76 and quinolines 77 (Scheme 29).^90^ The reaction mechanism was presented in Scheme 30. The electrophilic reaction of aldehyde 3 with two nucleophiles 1 and 74 resulted in the formation of intermediate A which was converted into a chromene-type intermediate B through an intramolecular substitution.^91^ Finally, hydrolysis of intermediate B formed 77. The reaction mechanism of aldehyde 75 is shown in Scheme 31. First, the condensation of 75 and indoles 1 produced the intermediate D. The NH_2_ group of 75 attacked the C2 position of the indole ring, and formed the intermediate E. Cleavage of an endocyclic C–N bond allowed the formation of a quinoline derivative F.^91^ Then, compound 74 reacted with NH_2_ group of quinoline F and final product 76 was obtained.

**Scheme 32:**
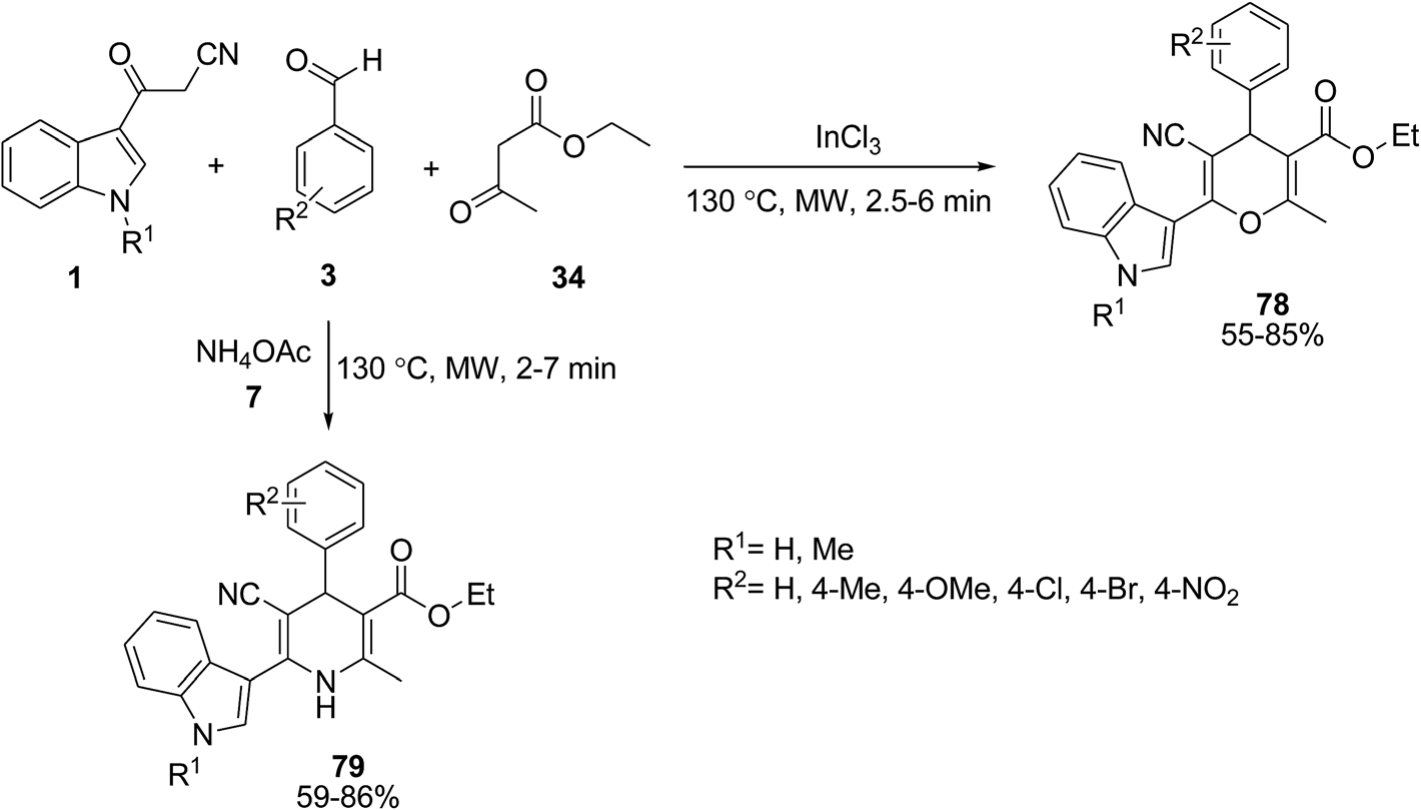


Borah *et al.* investigated the one-pot multicomponent reaction of 3-(cyanoacetyl)-indoles 1, aromatic aldehydes 3 and ethyl acetoacetate 34 in the presence of InCl_3_ under microwave irradiation to produce the functionalized 3-(pyranyl)-indole derivatives 78. When ammonium acetate 7 was used as the source of ammonia in this reaction, the one-pot four-component reaction was carried out and 3-(dihydropyridinyl)-indole derivatives 79 were obtained (Scheme 32).^92^ The results show that electron donating groups (EDG) in the aldehyde increase the product yield, whereas electron withdrawing groups (EWG) decrease the yield of products.

**Scheme 33:**
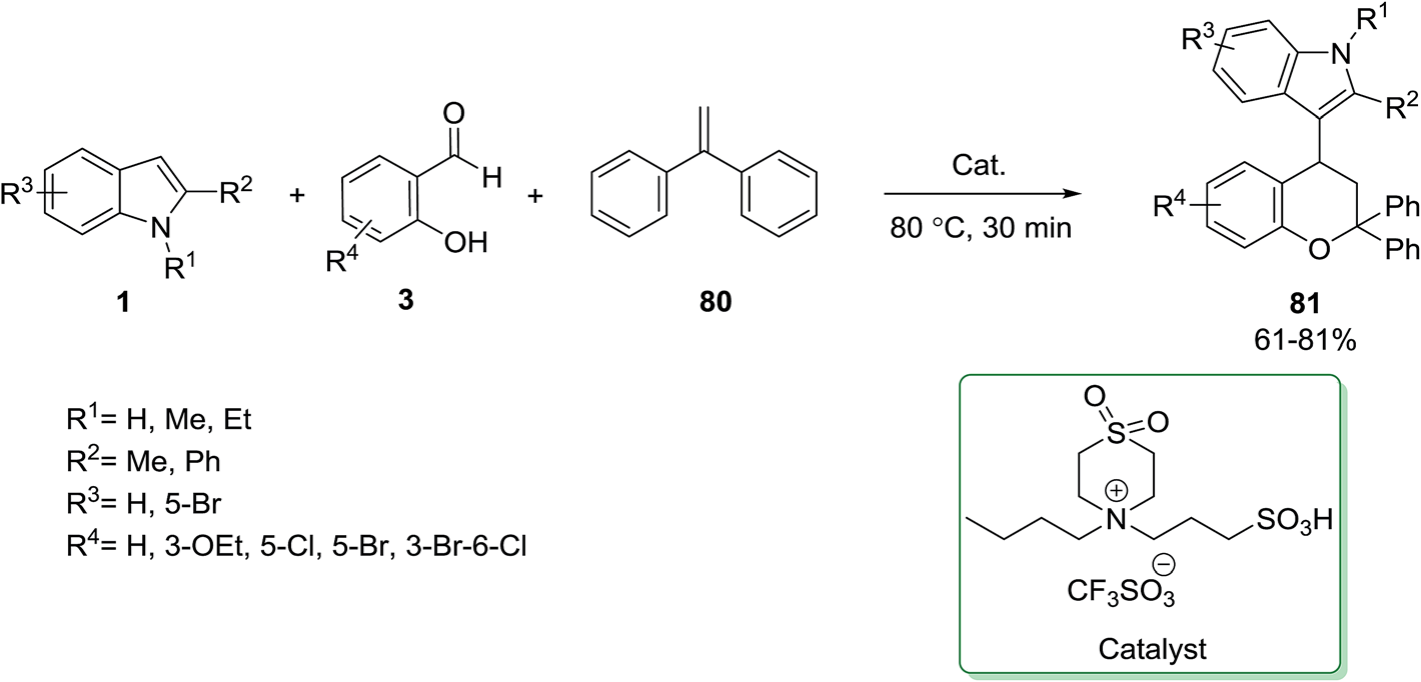


A sulfone-containing Brønsted acid ionic liquid was used in a one-pot reaction of indole 1, salicylaldehydes 3 and 1,1-diphenylethylene 80 for the synthesis of substituted chromane derivatives 81 (Scheme 33).^93^

**Scheme 34:**
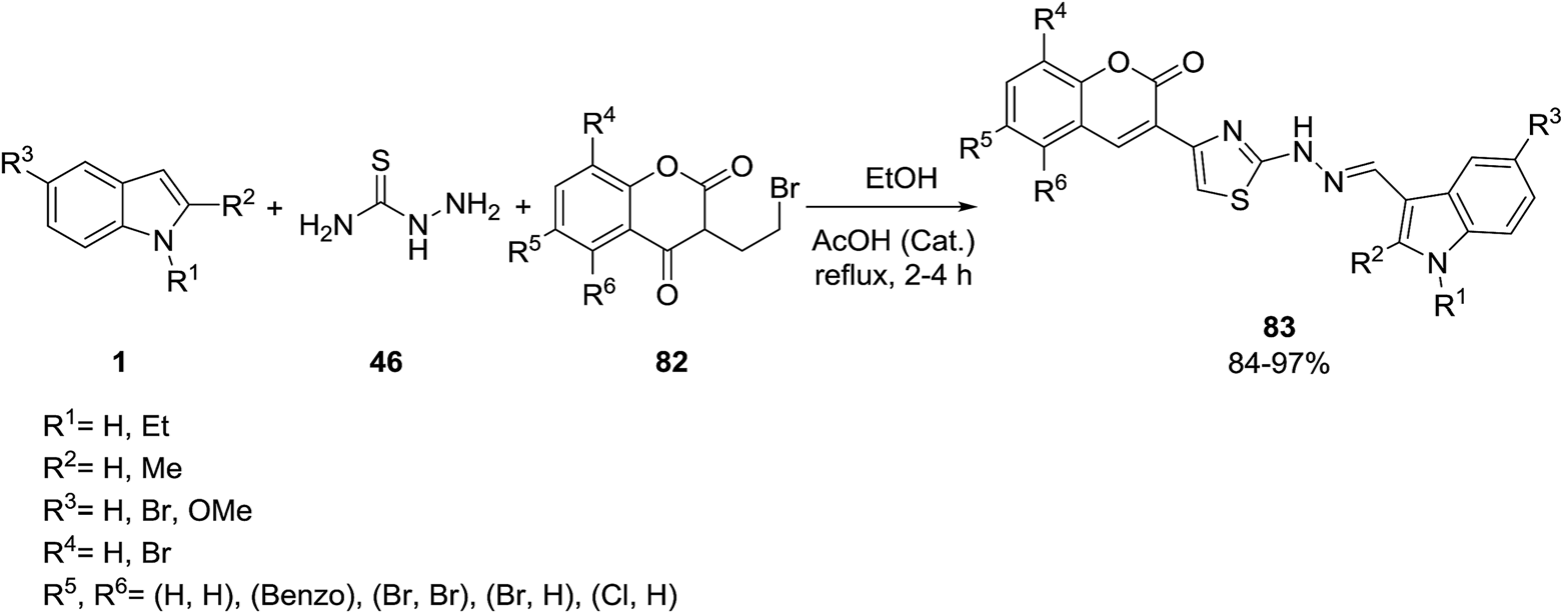


A series of indole incorporated thiazolylcoumarins 83 were synthesized from the reaction of indole derivatives 1, thiosemicarbazide 46 and 3-(2-bromoacetyl)-2*H*-chromen-2-ones^94^82 using a catalytic amount of acetic acid (Scheme 34).^95^ The antibacterial, anticancer and DNA cleavage activities of products were evaluated. The results showed that all products have a good activity towards the screened bacterial strains.

**Scheme 35:**
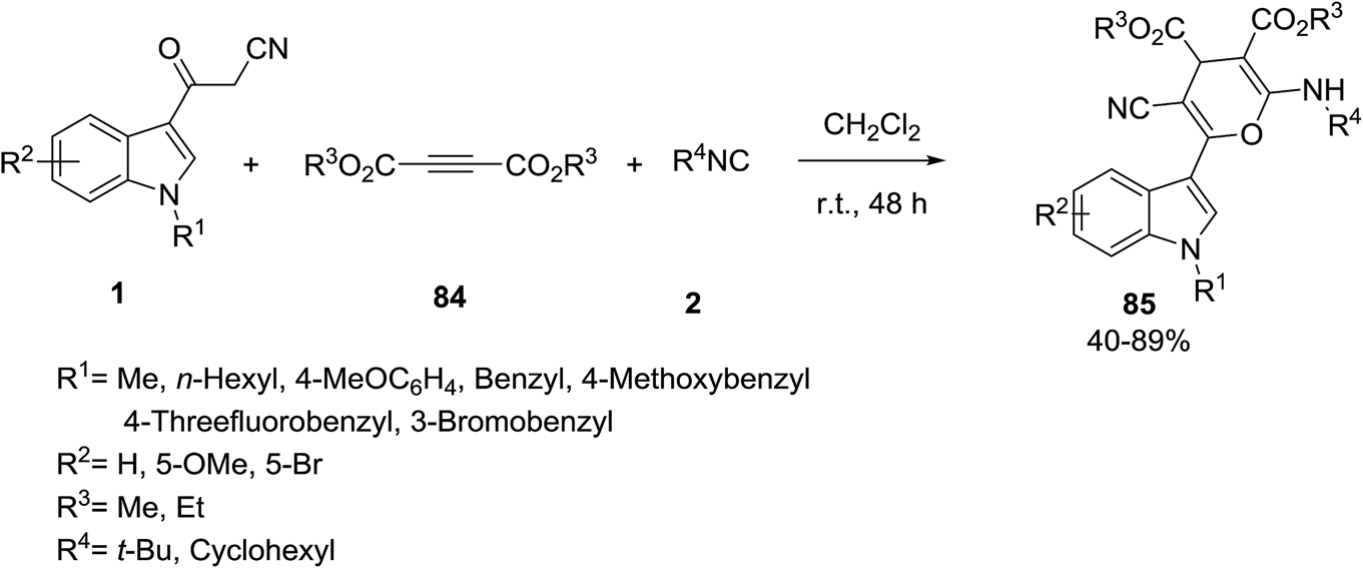


Song *et al.* established the [3 + 3] cyclization of 3-(cyanoacetyl)-indoles 1 with dialkyl acetylenedicarboxylates (DMAD) 84 and isocyanides 2 for the preparation of 4-*H*-pyran derivatives 85 containing an indole scaffold (Scheme 35).^96^

**Scheme 36:**
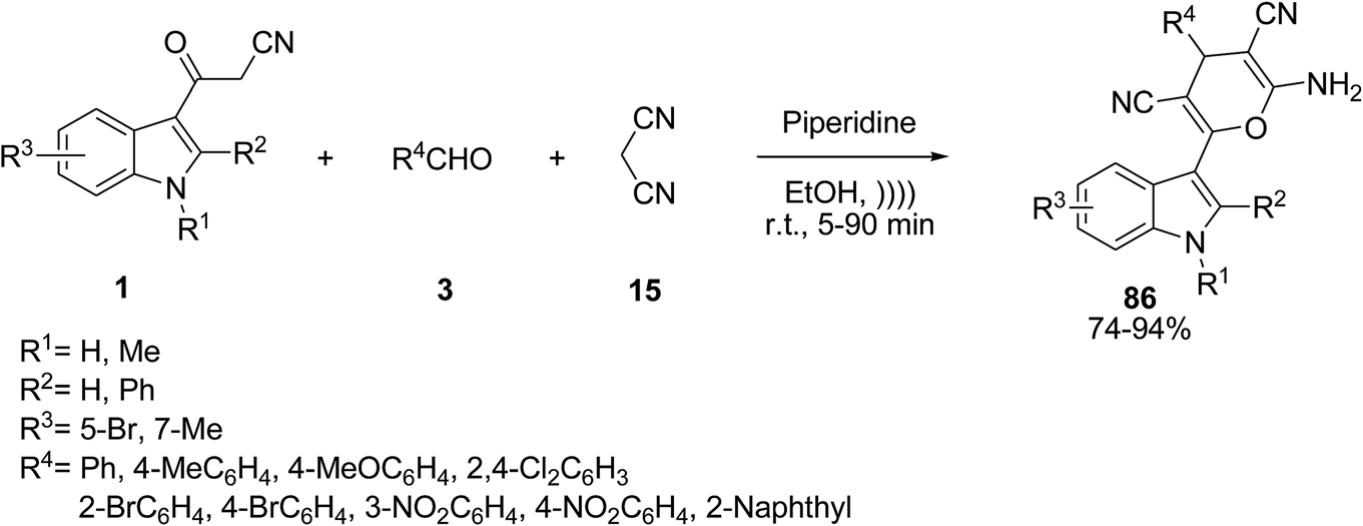


Efficient synthesis of indol-3-yl substituted pyran derivatives 86 was investigated by Ji and co-workers *via* the one-pot multicomponent reaction of 3-cyanoacetyl indoles 1, aldehydes 3 and malononitrile 15 in the present of piperidine as catalyst under ultrasonic irradiation (Scheme 36).^97^ Thiamine hydrochloride (vitamin B_1_) and cetyltrimethylammonium bromide (CTAB) were also utilized as catalysts in this reaction for 25 min giving 92–94% yield.^98^

**Scheme 37:**
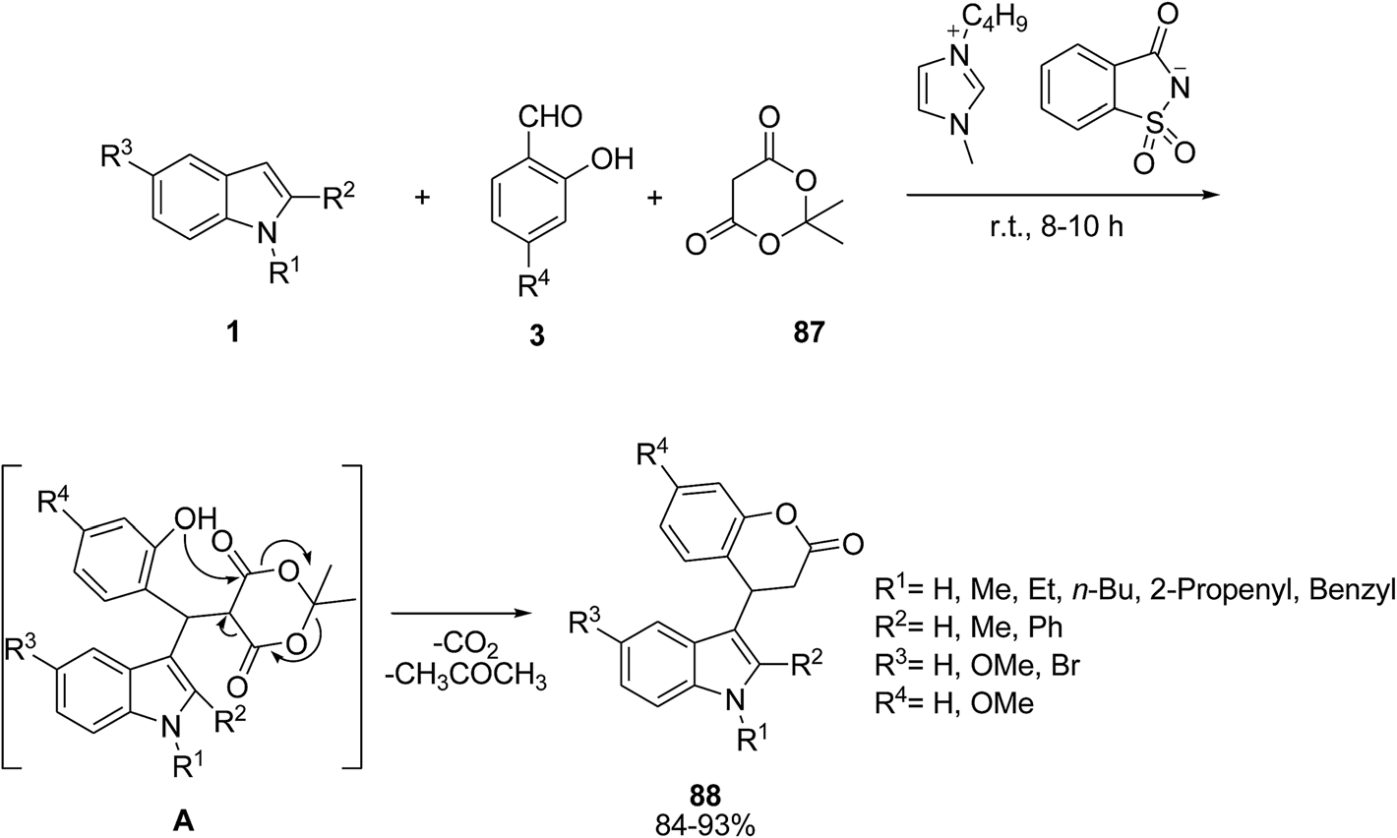


A Knoevenagel^99^ coupling of salicylaldehyde 3 and Meldrum’s acid 87 followed by a Michael type reaction with indole 1 in the presence of a (saccharin)-based functional ionic liquid (imidazolium saccharinate) was reported by Kumar *et al.* This multicomponent reaction was performed *via* lactonization decarboxylative elimination to functionalize the C-3 of indoles with dihydrocoumarin to yield indole-3-dihydro-coumarins 88 (Scheme 37).^100^

**Scheme 38:**
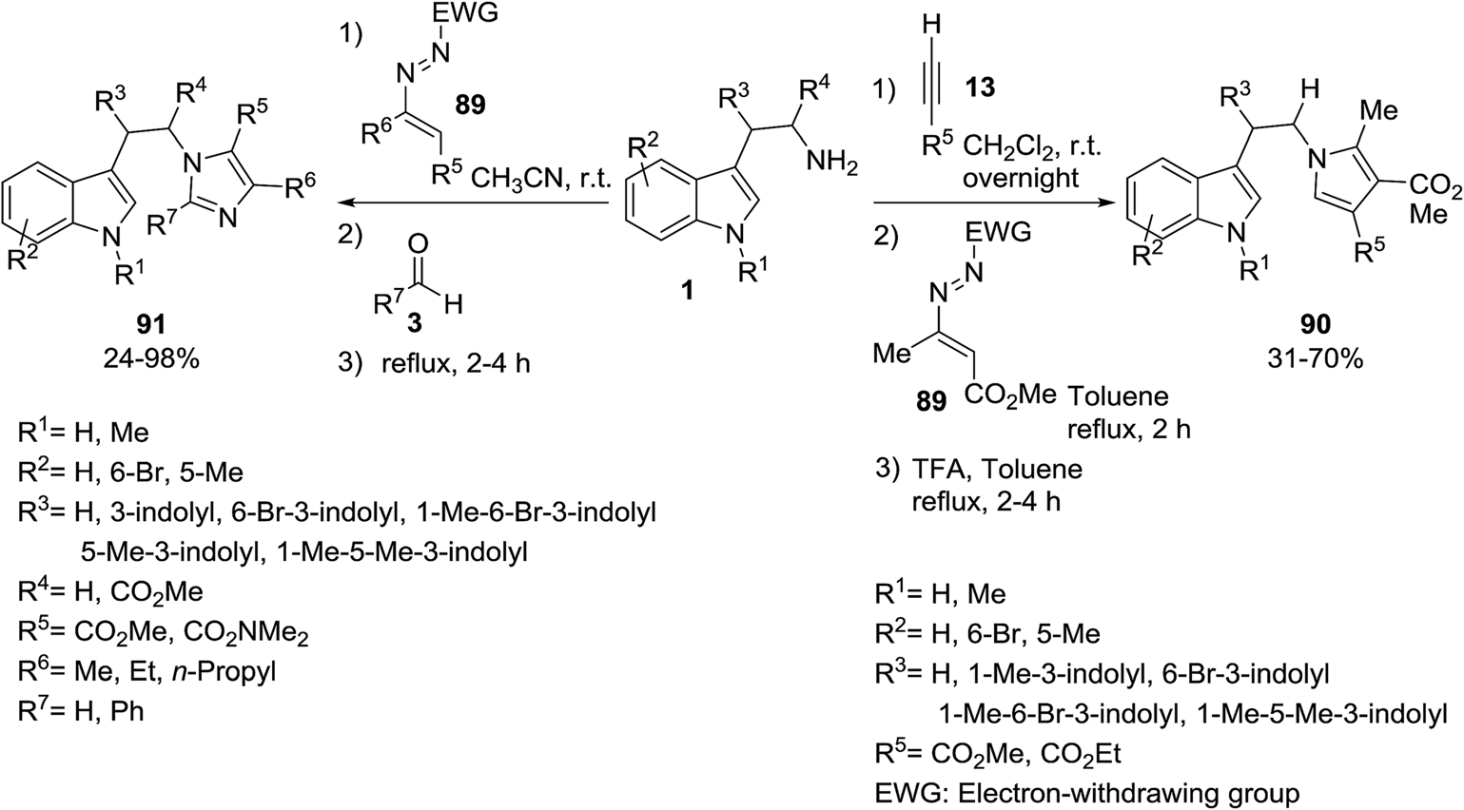


Biheterocycles containing indole and azole skeletons 90 and 91 were prepared from the multicomponent reaction of indoles 1, 1,2-diaza-1,3-dienes 89 and aldehydes 3 or alkynes 13 (Scheme 38).^101^ The synthesized compounds were screened for their *in vitro* biological studies. The results showed that some of them have anticancer activity against MCF7 and Caco-2 human tumor cell lines.

**Scheme 39:**
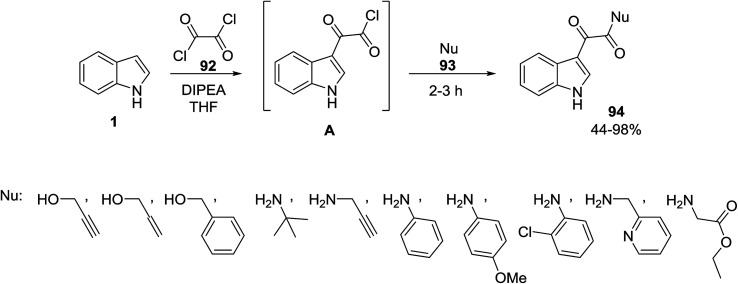


**Scheme 40:**
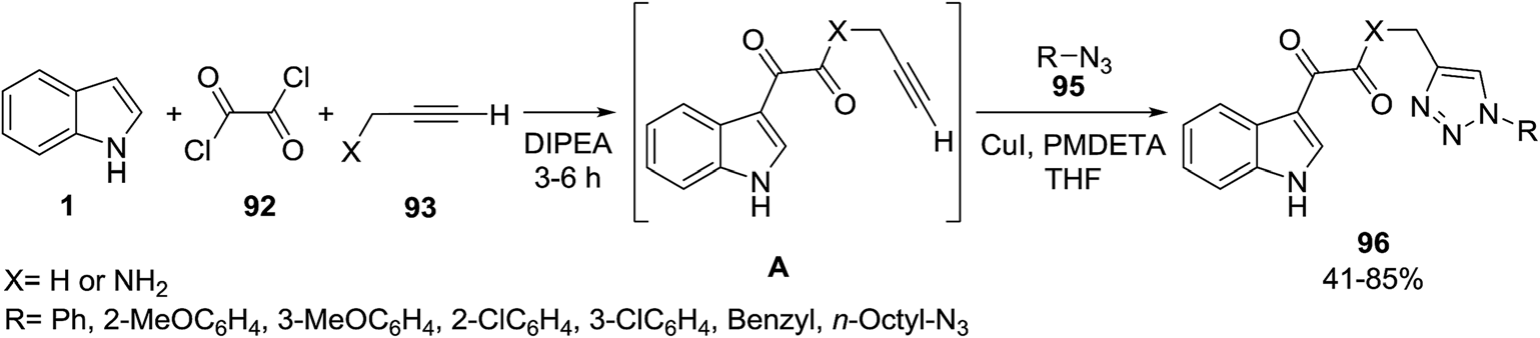


Stefani and co-workers utilized a mild approach for the synthesis of indole-3-glyoxyl derivatives 94 and indole-3-glyoxyl-1,2,3-triazoles 96. For this purpose, the reaction of indole 1, oxalyl chloride 92 and various nucleophiles 93 was carried out in *N*,*N*-diisopropylethylamine (DIPEA) to afford indole-3-glyoxyl derivatives 94 (Scheme 39). On the other hand, indole-3-glyoxyl-1,2,3-triazoles 96 were obtained from the one-pot multicomponent reaction of these reactants and organic azides 95*via* click^102^ chemistry (Scheme 40).^103^

**Scheme 41:**
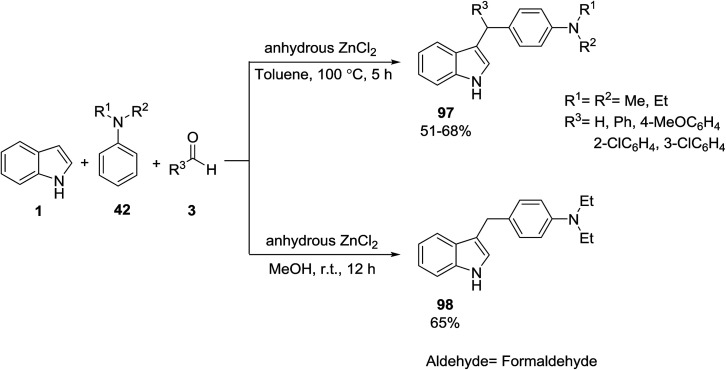


**Scheme 42:**
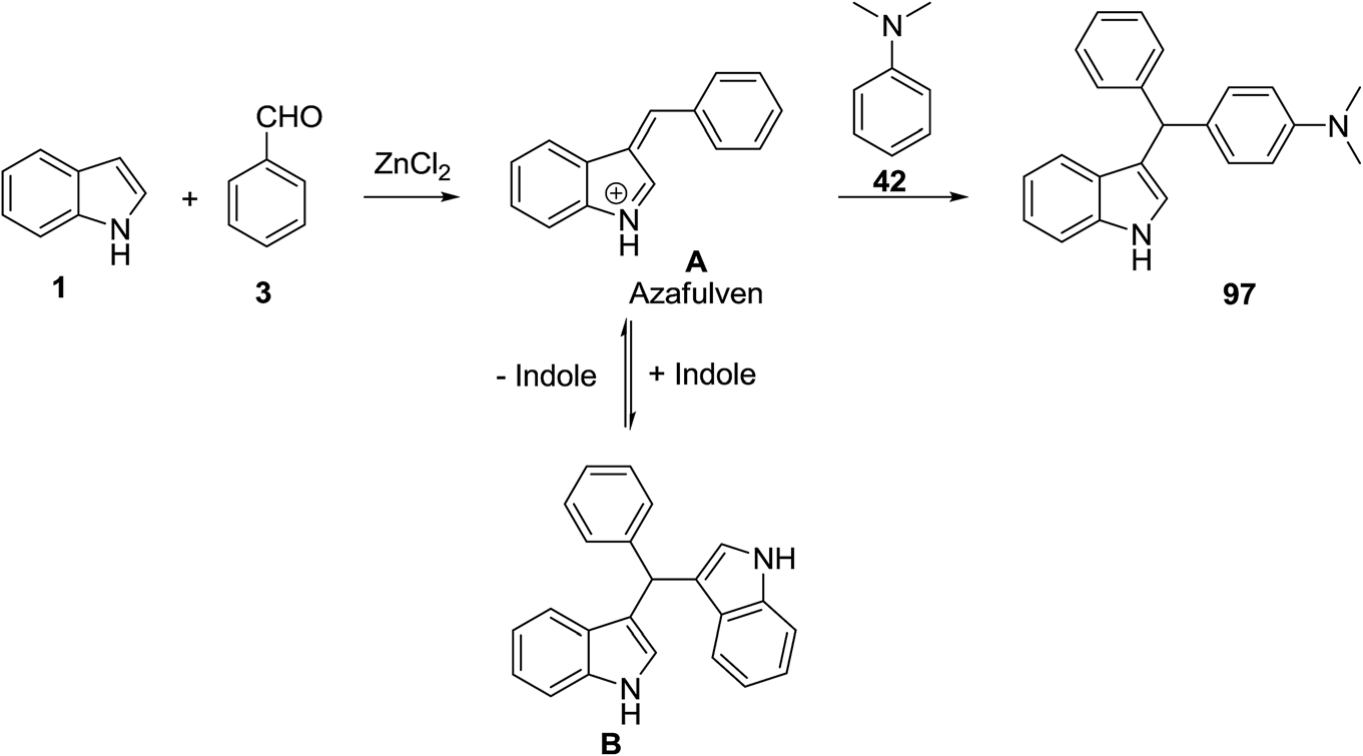


Indole 1 was reacted with anilines 42 and aldehydes 3*via* an anhydrous ZnCl_2_ catalyzed one-pot three-component reaction to afford diarylmethyl indoles 97 in toluene and 3-arylmethyl indoles 98 in MeOH. The reaction of indole 1 with benzaldehyde 3 in the presence of catalyst formed the azafulven A which reacted with another indole 1 to generate the kinetically stable bis(indolyl)methane B. In the presence of anilines, bis(indolyl)methane converted to the target products 97 (Schemes 41 and 42).^104^

**Scheme 43:**
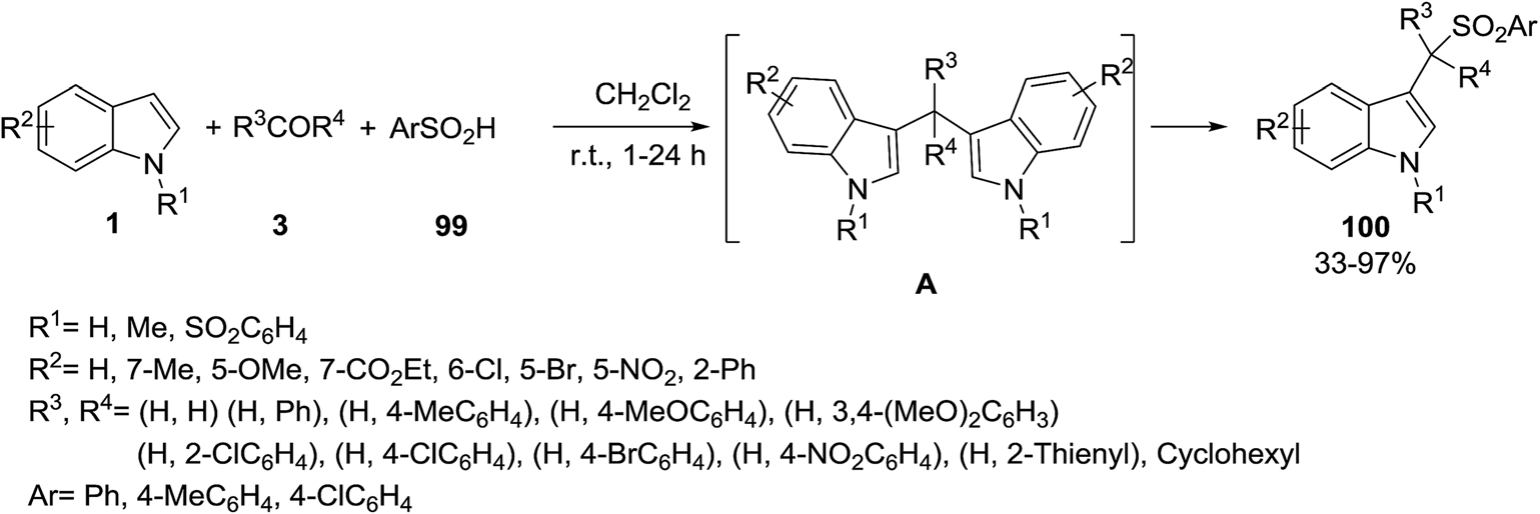


A series of biologically important 3-(1-arylsulfonylalkyl)indoles 100 were prepared by Huang *et al.* This process was carried out using a catalyst-free three-component reaction of indoles 1, carbonyl compounds 3, and arenesulfinic acids 99 at room temperature (Scheme 43).^105^ Bis(indolyl)methanes A were found as the key intermediates in this reaction.

**Scheme 44:**
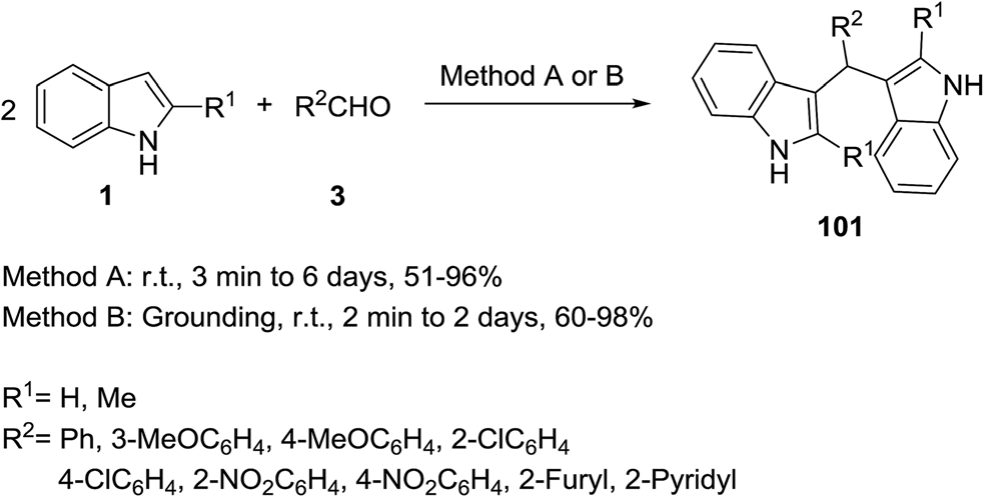


The synthesis of bis(indolyl)methanes 101 was reported by Dhumaskar and Tilve *via* the reaction of two molecules of indole 1 with aldehydes 3 under solvent-free conditions without any catalyst (Scheme 44).^106^ Two different methods were employed for this reaction. In method A, the mixture of aldehyde and indole was kept at ambient temperature in a test tube while in method B, the mixture was ground using a mortar and pestle. Method B resulted in the formation of products in shorter reaction times than method A. According to the results, anisaldehyde with an electron donating group at the *para* position and heptaldehyde failed to react completely. Mohammadi Ziarani and co-workers developed this reaction using the sulfonic acid functionalized silica (SiO_2_-Pr-SO_3_H) as catalyst and obtained the same products in short reaction times and good yields (5–20 min and 87–96% yields).^107^

**Scheme 45:**
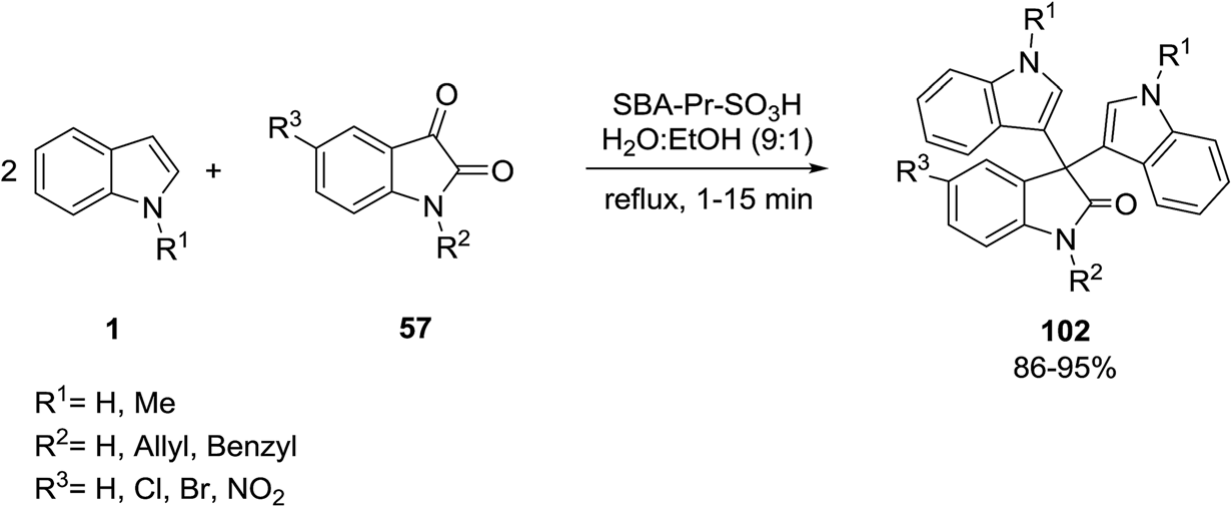


Mohammadi Ziarani and coworkers synthesized a novel class of symmetrical 3,3-di(indolyl)indolin-2-ones 102 from the reaction of indoles 1 with isatins 57 in the presence of SBA-Pr-SO_3_H as a solid acid catalyst under mild reaction conditions (Scheme 45).^108^ The antimicrobial activities of the products were tested and the results demonstrated that the MIC value of one of the products (R^1^ = R^3^ = H, R^2^ = Bn) against *B. subtilis* was equal to that of chloramphenicol.

**Scheme 46:**
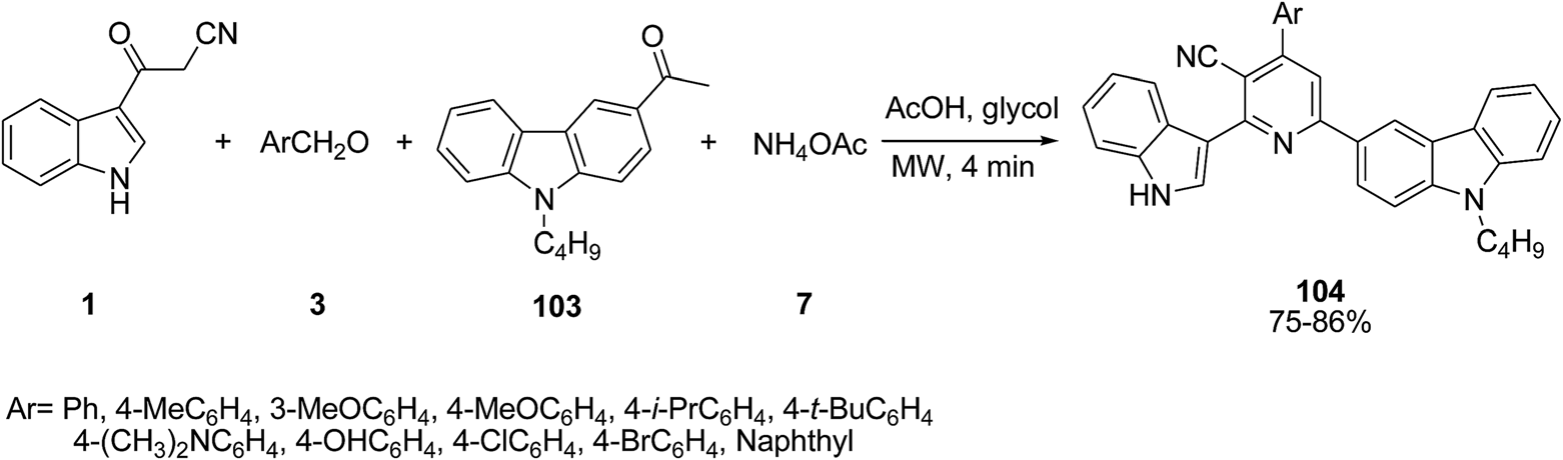


Geng’s group developed the one-pot four component reaction of 3-(cyanoacetyl)indoles 1, aromatic aldehydes 3, 1-(9-butylcarbazol-3-yl)ethanone 103 and ammonium acetate 7 for the preparation of several 3-cyano-2-(1*H*-indol-3-yl)-6-(9-butylcarbazol-3-yl)pyridine derivatives 104. The reaction was performed using AcOH and ethane-1,2-diol (glycol) under microwave irradiation (Scheme 46).^109^

**Scheme 47:**
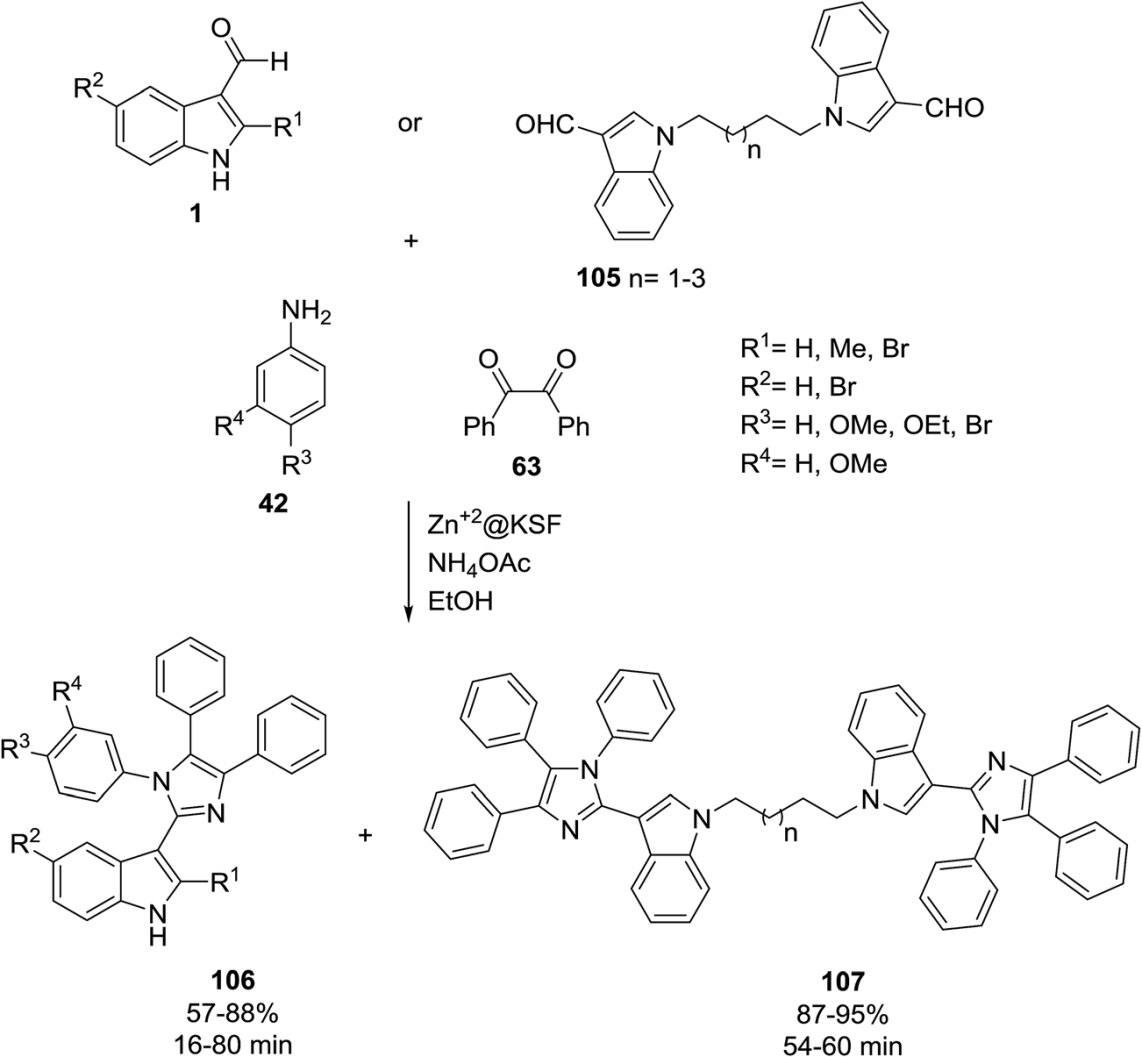


Zn^2+^ supported on montmorillonite KSF (Zn^2+^@KSF) as an efficient heterogeneous catalyst promoted the preparation of mono and bis-indolylimidazole derivatives 106 and 107. This reaction was carried out from the condensation of indole-3-carbaldehyde derivatives 1 or bis-aldehydes 105,^110^ aniline derivatives 42 and benzil 63 (Scheme 47).^111^ The antibacterial activity of the synthesized compounds was examined and some of them exhibited promising activities.

**Scheme 48:**
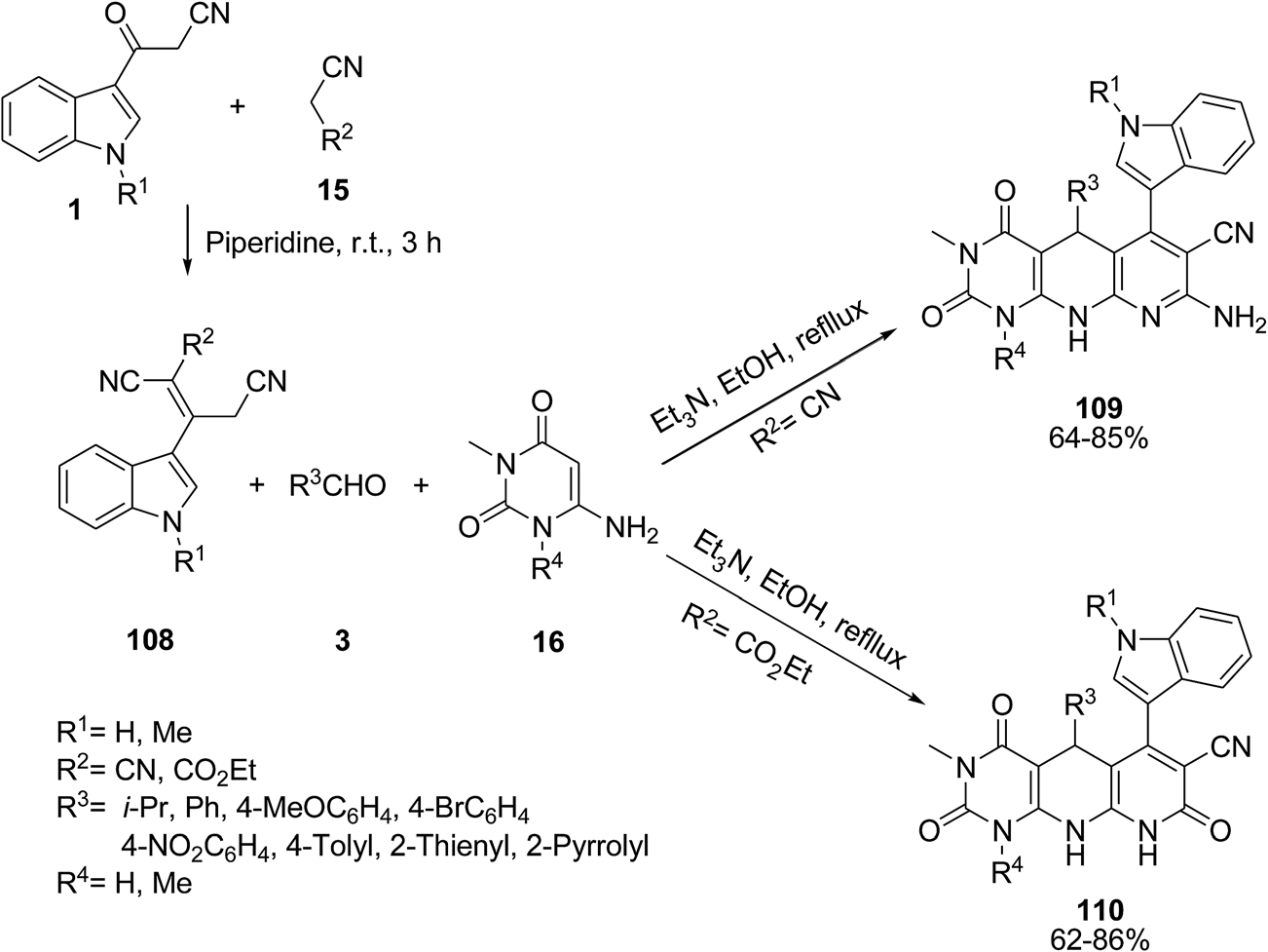


Naidu *et al.* explored the one-pot three-component reaction of 2-cyano-3-(1*H*-indol-3-yl)-pent-2-enedinitrile or ethyl-2,4-dicyano-3-(1*H*-indol-3-yl)but-2-enoate derivative 108, aryl aldehydes 3 and 6-aminouracil derivatives 16 (obtained from the reaction of 3-(cyanoacetyl)-indoles 1 and nitrile 14) for the synthesis of some hexahydropyrimido[4,5-*b*]-1,8-naphthyridine derivatives 109 and 110. It was found that when the indole derivative 108 (R^1^ = H; R^2^ = CO_2_Et) was treated with benzaldehyde (4a) and uracil 16 (R^4^ = Me) the ester group participated in the cyclization process instead of the nitrile group, and the trioxo-nitrile compound 110 was obtained (Scheme 48).^112^

**Scheme 49:**
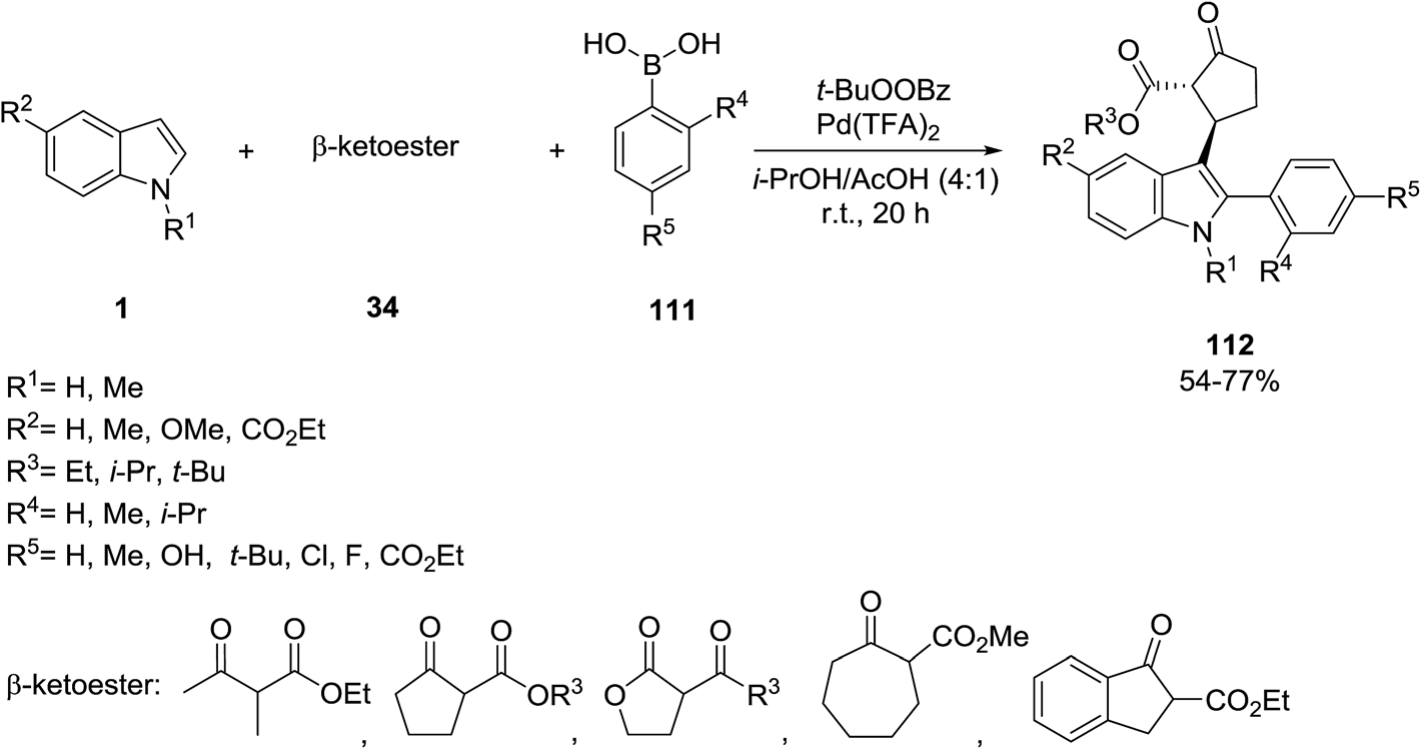


The three-component coupling reaction of indoles 1, β-ketoesters 34 and arylboronicacids 111*via* the regioselective palladium-catalyzed oxidative reaction was studied for the preparation of indole-based heterocycles 112 (Scheme 49).^113^ Indole coupled more rapidly than β-ketoester with the arylboronic acid, and this rate variation was important under the three-component reaction conditions, possibly because the coupling with β-ketoester involves an unstable enone intermediate.

**Scheme 50:**
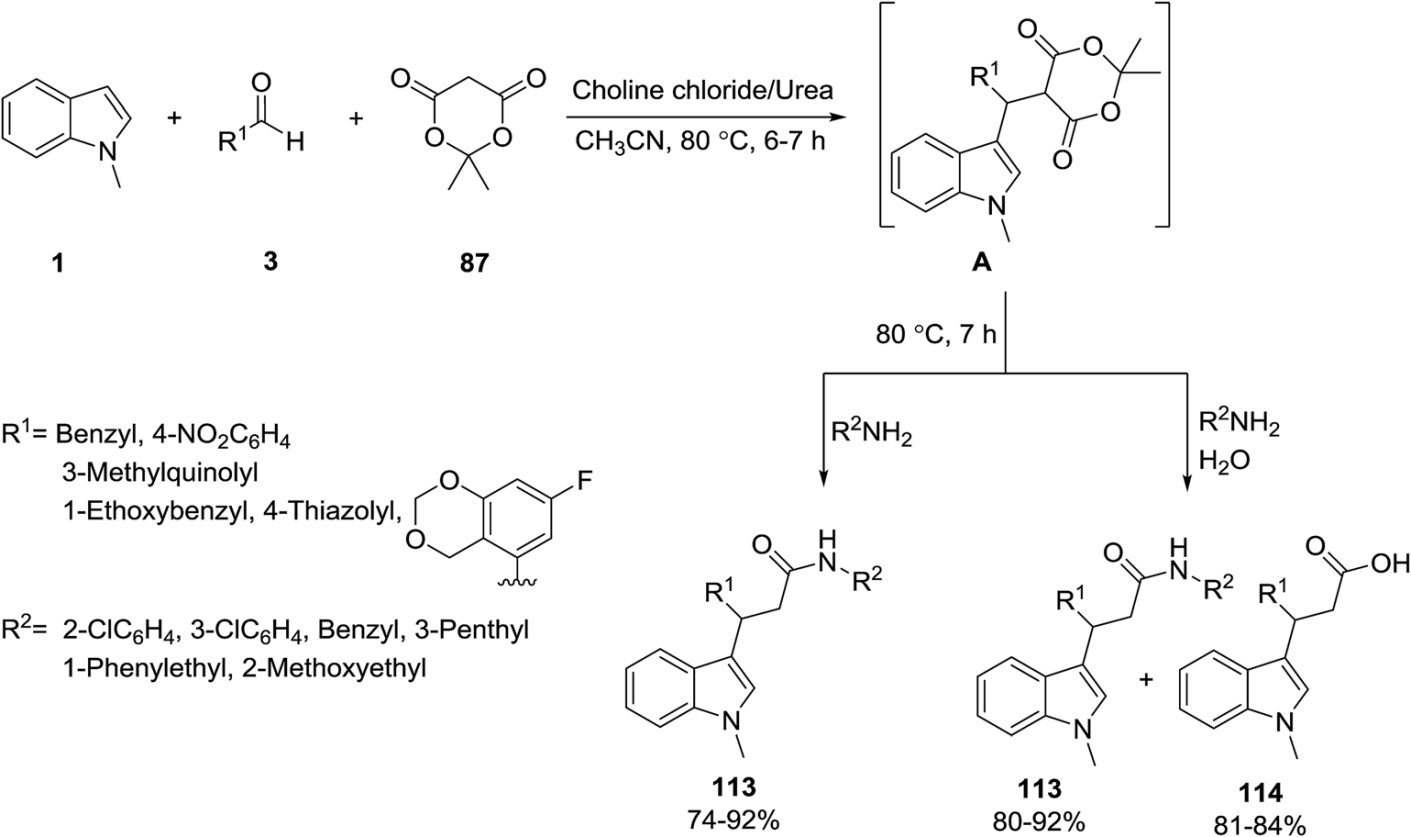


Siddalingamurthy *et al.* described the synthesis of indole-3-propanamide derivatives 113 and 114*via* the three-component reaction of *N*-methyl indole 1, aromatic aldehydes 3 and Meldrum’s acid 87 in the presence of choline chloride/urea ionic liquid as catalyst. At first, Meldrum’s adduct A was generated, which then reacted with various amines, and the indole-3-propanamide derivatives 113 were formed. On the other hand, when Meldrum’s adduct was treated with H_2_O, corresponding acid products 114 were obtained (Scheme 50).^114^

**Scheme 51:**
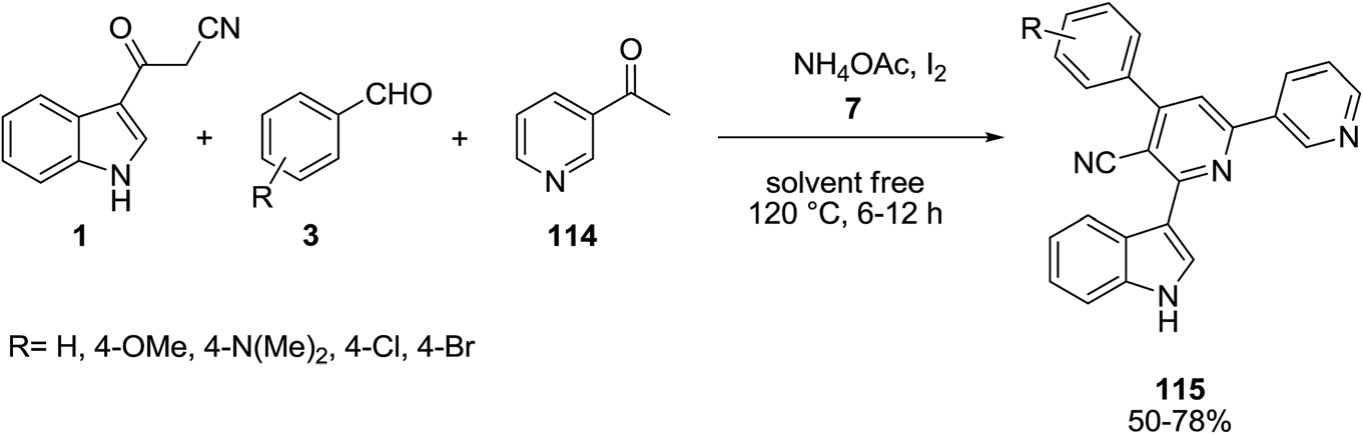


**Scheme 52:**
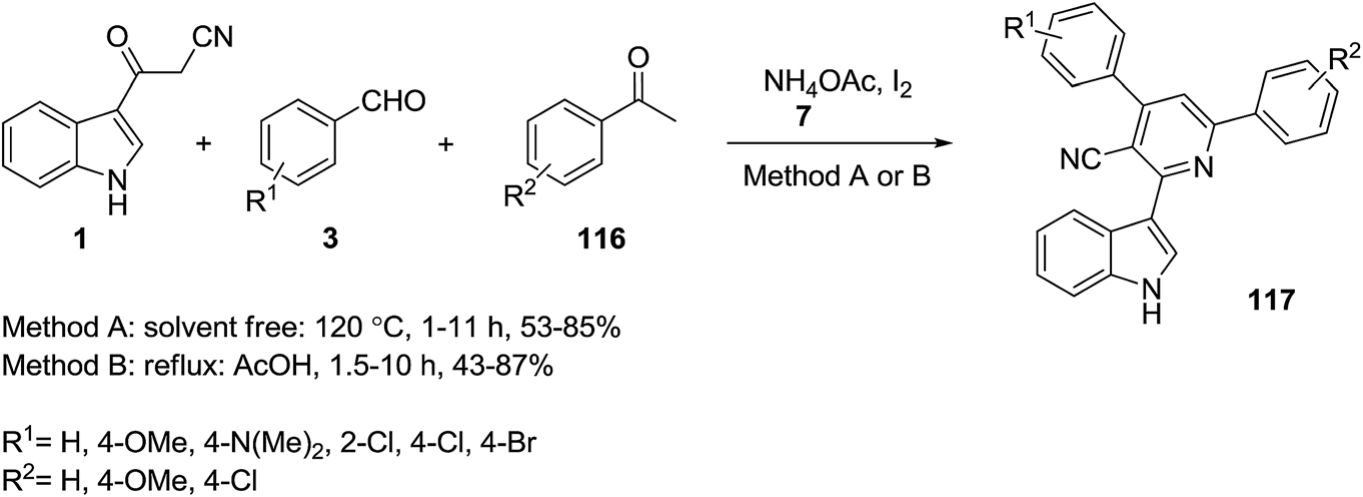


A one-pot four-component reaction of 3-(cyanoacetyl)indole 1, benzaldehyde derivatives 3, 3-acetylpyridine 114, and NH_4_OAc 7 was explored for the preparation of 2-(indol-3-yl)pyridine derivatives 115 (Scheme 51). In the same paper, 4-aryl-2-(1*H*-indol-3-yl)-6-arylnicotinonitriles 117 were also obtained based on this process, from the reaction of 3-(cyanoacetyl)indole 1, aromatic aldehydes 3, aromatic ketones 116, and NH_4_OAc 7 under two different conditions (Scheme 52).^115^

**Scheme 53:**
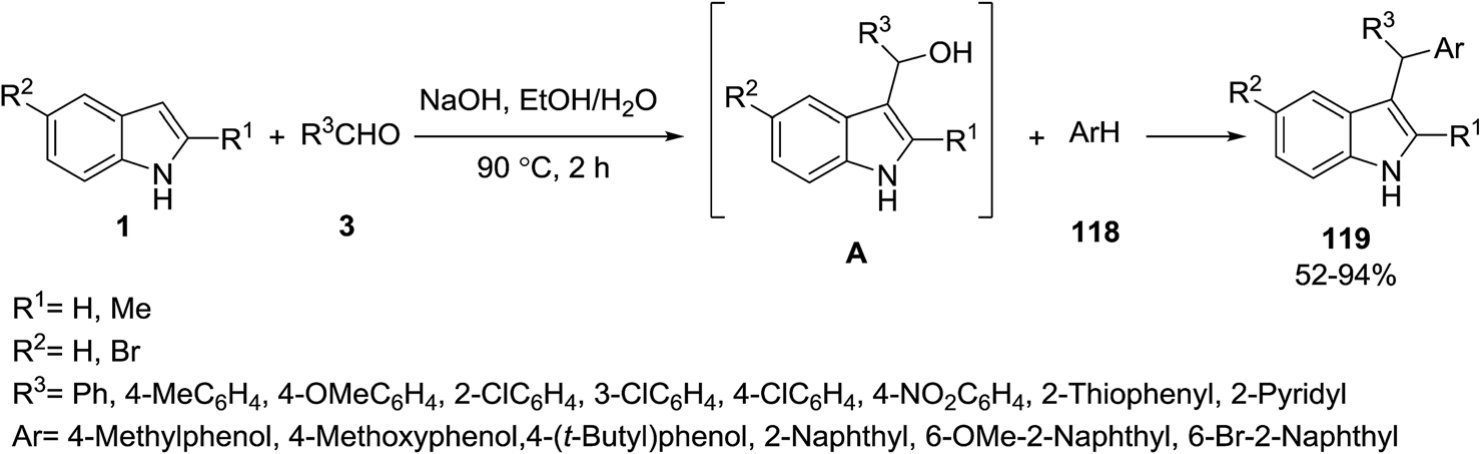


Deb *et al.* reported a base-promoted three-component one-pot approach for the synthesis of 3-(α,α-diarylmethyl) indoles 119*via* arylation of *in situ* generated 3-indolylalcohols A in an EtOH–H_2_O solvent system. Substituted indoles 1, benzaldehydes 3 and electron-rich aromatics 118 were used as starting materials in this reaction (Scheme 53).^116^

### 2.2. The N position reactions of indoles

**Scheme 54:**
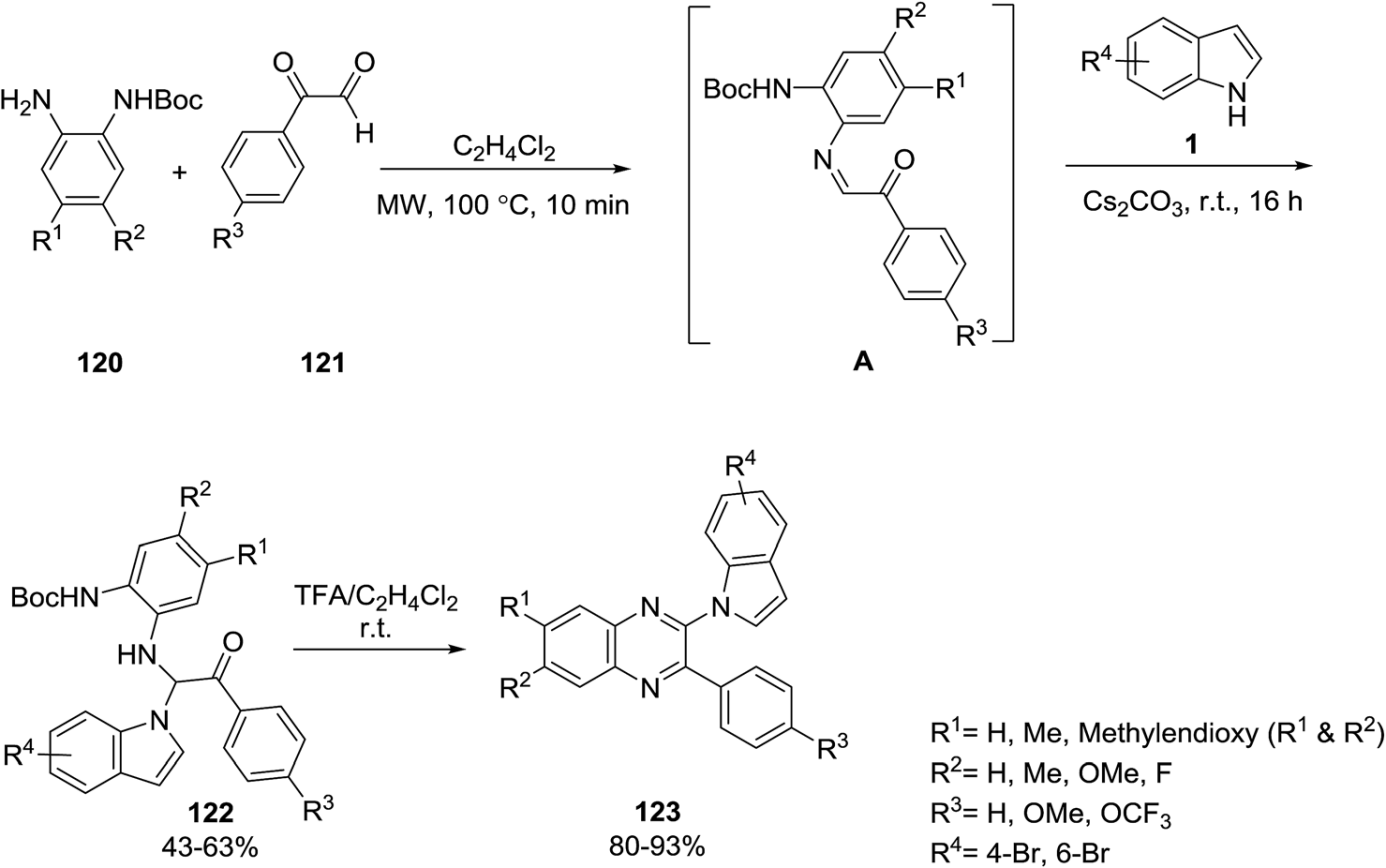


Martinez-Ariza *et al.* reported a two-step one-pot procedure for the synthesis of *N*-1-quinoxaline-indoles 123 from the reaction of indoles 1, amines 120 and glyoxaldehydes 121. In the first step, α-iminoketones A were formed from the reaction of amines 120 and glyoxaldehydes 121, which were then treated with indole 1 for the preparation of compounds 122. The deprotection-cyclization of 122 was performed for the synthesis of target products 123 (Scheme 54).^117^

**Scheme 55:**
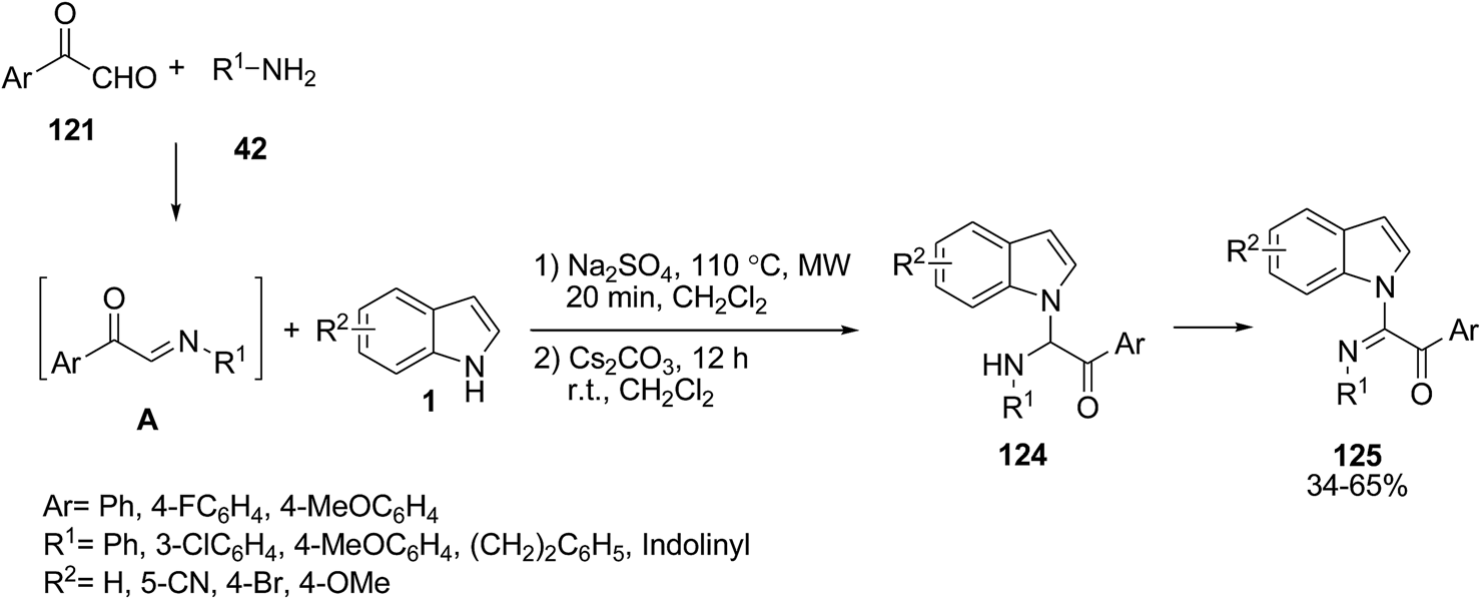


Hulme and co-workers reported a one-pot two-step multicomponent reaction of indole 1, arylglyoxaldehyes 121 and amines 42 for the synthesis of α-oxo-acetamidines 125 under microwave conditions. The main reaction step was the N-1 addition of indole to α-iminoketones A (obtained from the reaction of arylglyoxaldehyes 121 and amines 42), followed by an air- or oxygen-mediated oxidation (Scheme 55).^118^

### 2.3. The cycloaddition reactions of indoles

#### 2.3.1. The cycloaddition reactions of indoles at the C2–C3 π-bond

**Scheme 56:**
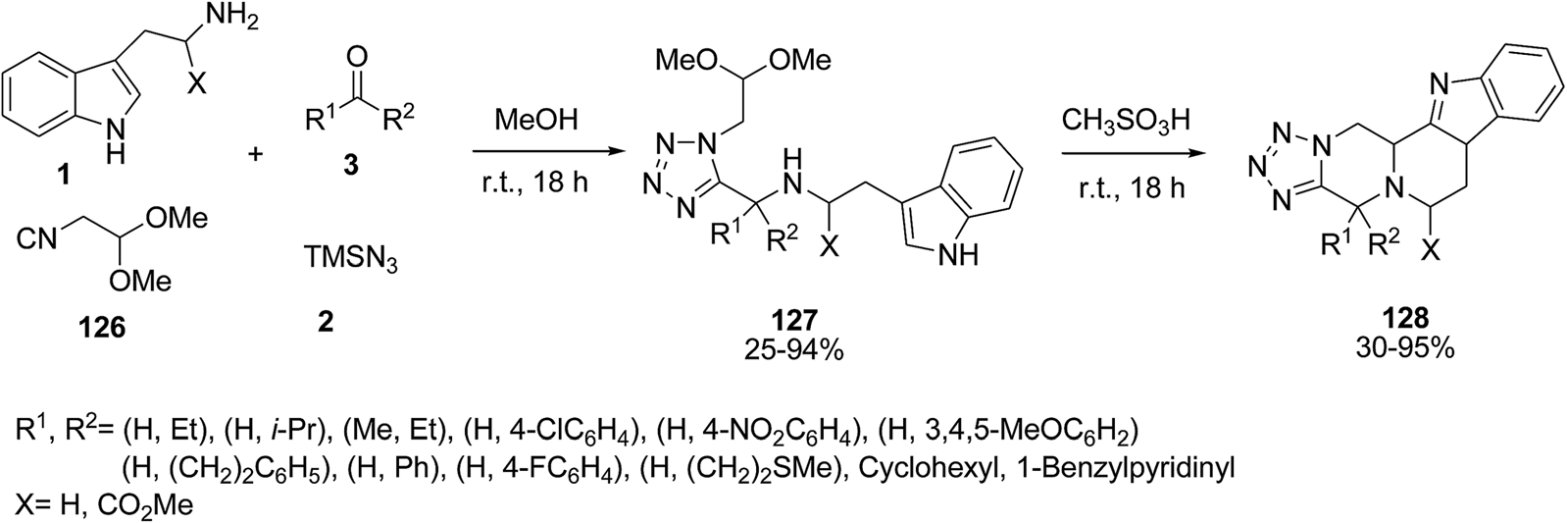


The tetracyclic tetrazole scaffold compounds 128 were constructed *via* a one-pot four-component Ugi–Pictet–Spengler reaction^119^ of indole 1, carbonyl compounds 3, isocyanoacetaldehyde (dimethylacetal) 126 and trimethylsilyl azide (TMSN_3_) 2. The reaction was carried out using methanesulfonic acid without any solvent at room temperature. First, the Ugi reaction was performed to obtain compounds 127, which was then followed immediately by the Pictet–Spengler reaction for the preparation of final products 128 (Scheme 56).^120^

**Scheme 57:**
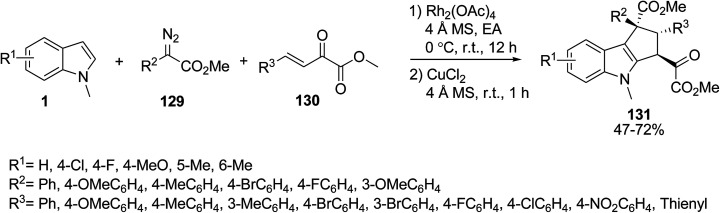


Jiang *et al.* described an aerobic dehydrogenative coupling interaction between indole derivatives 1, diazoacetates 129 and conjugated α-keto esters 130 in the presence of sequential Rh_2_(OAc)_4_ and CuCl_2_ as catalysts for the synthesis of polyfunctional cyclopenta[*b*]indoles 131 (Scheme 57).^121^

**Scheme 58:**
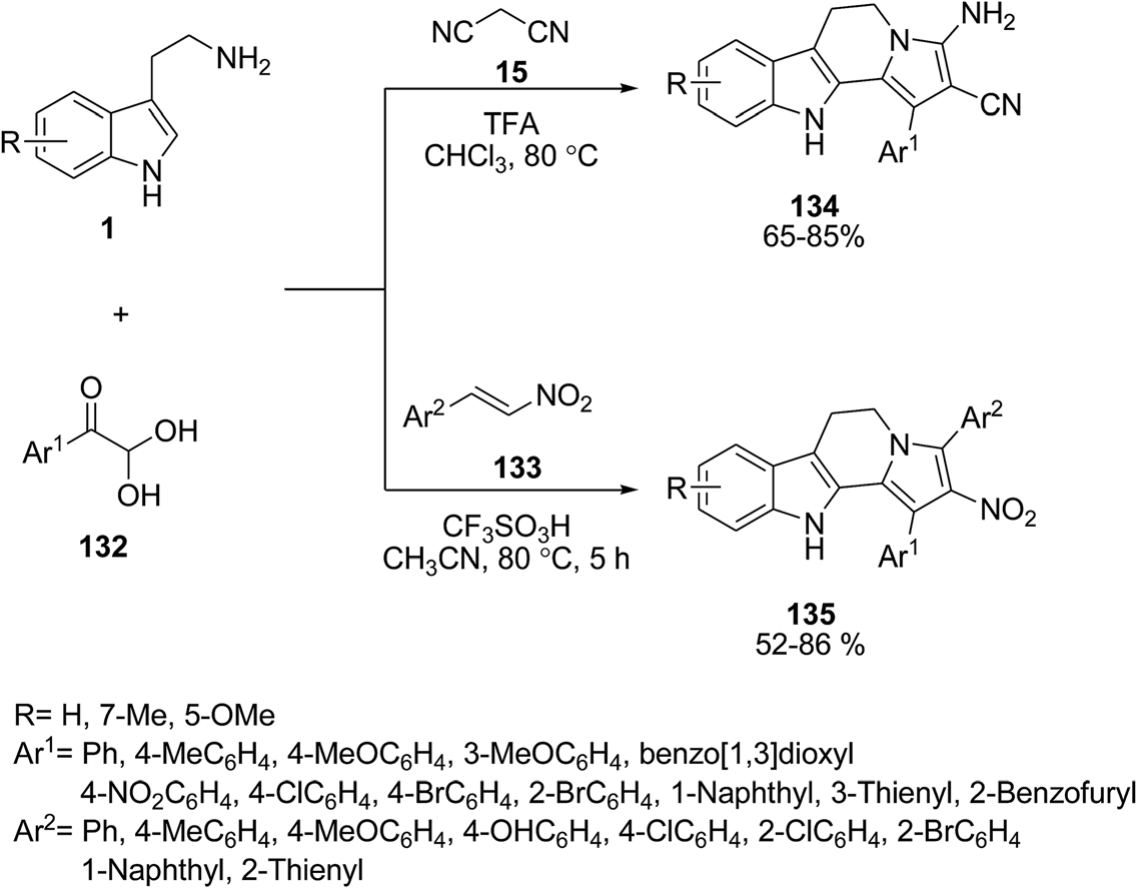


A acid-catalyzed multicomponent tandem cyclization protocol was applied by Cai *et al.* for the preparation of polyfunctional dihydroindolizino[8,7-*b*]indoles 134 and 135. The process involves the reaction of indole derivatives 1, arylglyoxal monohydrates 132 and *trans*-β-nitrostyrenes 133 or malononitrile 15 under mild, metal-free conditions (Scheme 58).^122^

**Scheme 59:**
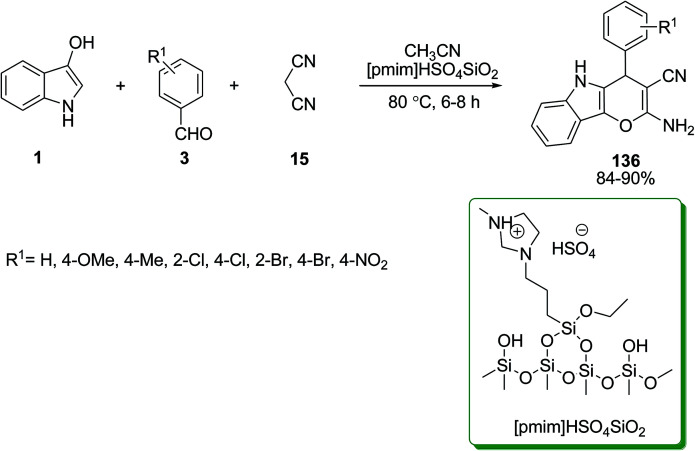


The silica supported ionic liquid of [pmim]HSO_4_SiO_2_ (silica supported 1-methyl-3-(triethoxysilylpropyl)imidazolium hydrogen sulfate) was used as an efficient catalyst for the synthesis of 2-amino-4,5-dihydro-4-arylpyrano[3,2-*b*]indole-3-carbonitrile derivatives 136. The reaction was carried out *via* the three-component condensation of indoles 1, aromatic aldehydes 3 and malononitrile 15 (Scheme 59).^123^ Other catalysts such as KHPO_4_ under ultrasonic irradiation^124^ and triphenylphosphine (PPh_3_)^125^ were also used in this reaction as demonstrated in Table 4.

**Table 4:** Comparison of different conditions for the synthesis of product 136

Entry	Solvent	Catalyst	Temperature (°C)	Time (min)	Yield (%)
1	CH_3_CN	[pmim]HSO_4_SiO_2_	80	6–8 h	84–90 (ref. 123)
2	EtOH	KHPO_4_	60	30	85–92 (ref. 124)
3	H_2_O–EtOH	PPh_3_	60	30–50	85–92 (ref. 125)

**Scheme 60:**
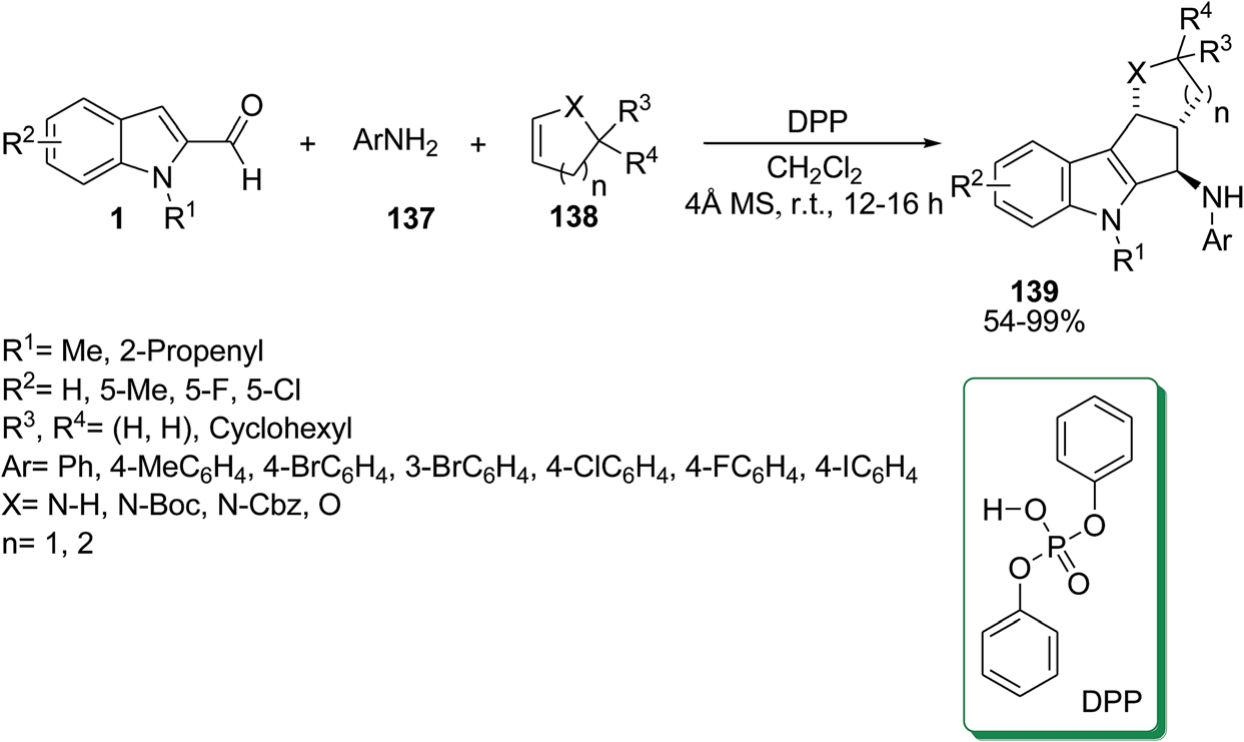


**Scheme 61:**
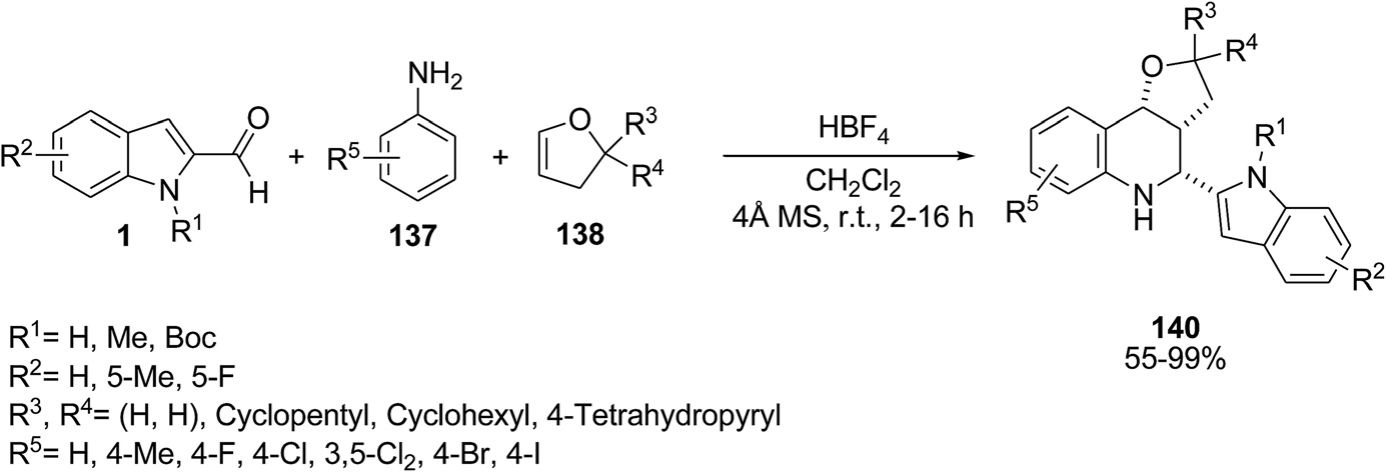


Galvan *et al.* accomplished the reaction of indole-2-aldehydes 1, imines 137 and alkenes 138*via* stereoselective [3 + 2] carbocyclization to achieve cyclopenta[*b*]indoles 139 or tetrahydroquinolines 140. It was found that the model coupling reaction could be changed to give related products 139 or 140 in the presence of different acid catalysts (Schemes 60 and 61).^126^

**Scheme 62:**
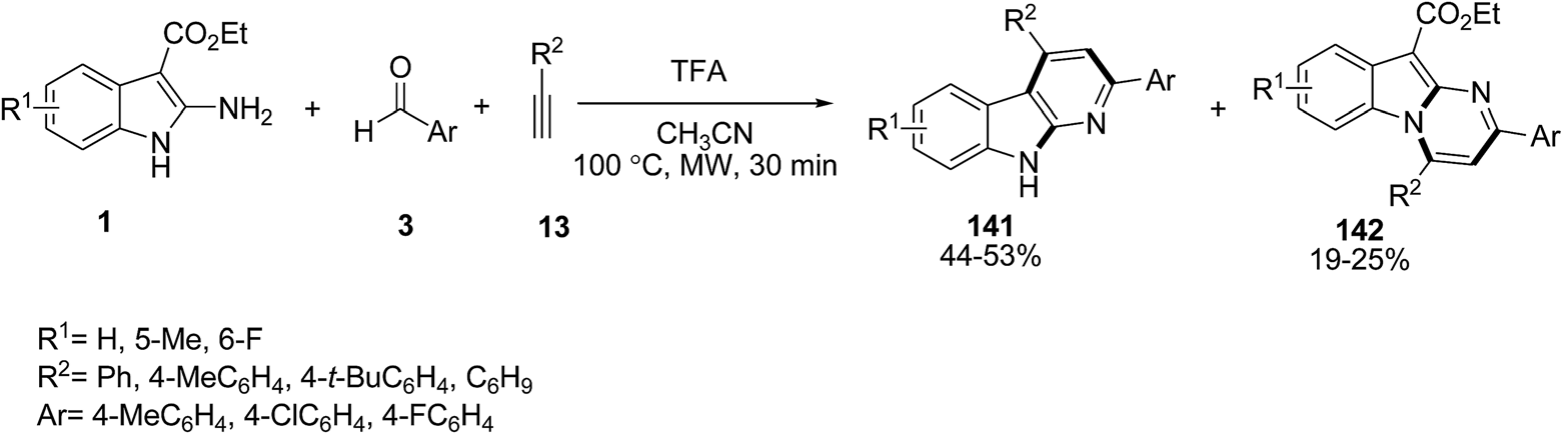


Kundu and co-workers established a new synthetic protocol for the preparation of pyrido- and pyrimido-indoles 141 and 142 employing ethyl 2-amino-1*H*-indole-3-carboxylates 1, aromatic aldehydes 3, and terminal alkynes 13 in the presence of a Brønsted acid such as trifluoroacetic acid (TFA) (Scheme 62).^127^ When the reaction catalyzed by Yb(OTf)_3_ as a Lewis acid, pyrimidoindoles 142 were prepared as the single products in 58–75% yield.

**Scheme 63:**
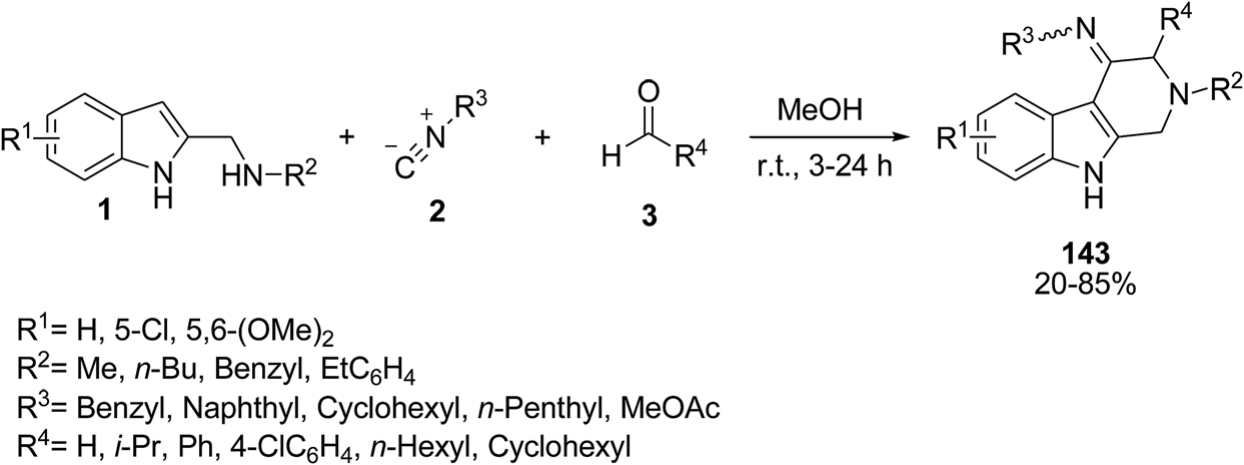


Tron’s group have prepared heteroarylogous 1*H*-indole-3-carboxamidines 143 utilizing a three-component interrupted Ugi reaction of *N*-alkyl-*N*-(1*H*-indol-2-ylmethyl)amines 1,^128^ isocyanides 2 and carbonyl compounds 3 (Scheme 63).^129^

**Scheme 64:**
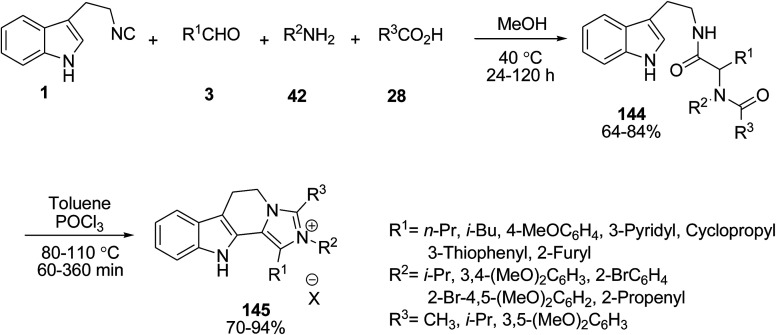


Silvani *et al.* reported an impressive method to prepare dihydroimidazo[1′,5′:1,2]pyrido[3,4-*b*]indol-2-ium salts 145 utilizing a Ugi/Bischler-Napieralski/heterocyclization MCR. The Ugi reaction was carried out following a general procedure, consisting of the sequential addition of aldehydes 3, amines 42, acids 28 and, finally, indole 1 for the synthesis of compounds 144. Treatment of intermediate compounds 144 in MeOH under the Bischler–Napieralski and heterocyclization gave the intended products 145 (Scheme 64).^130^

**Scheme 65:**
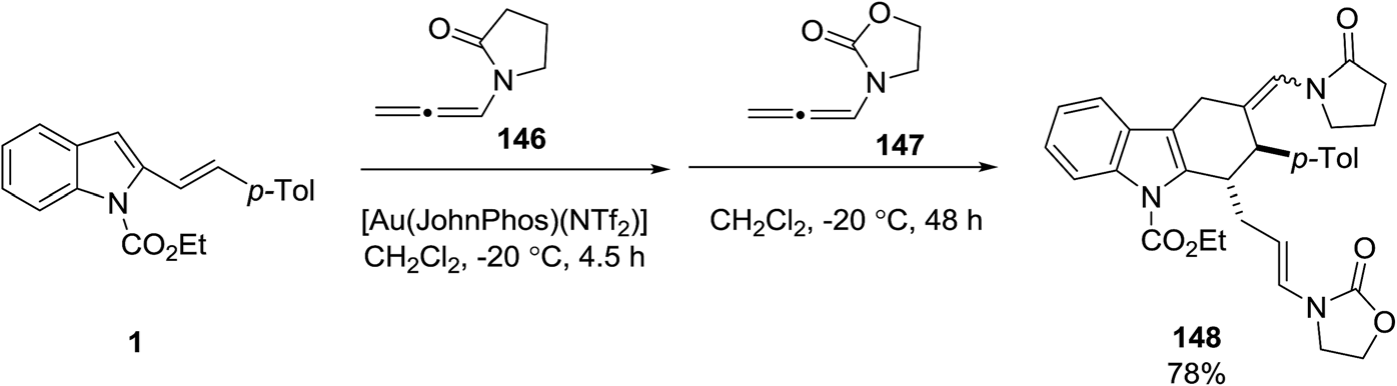


A gold-catalyzed multicomponent reaction of vinyl indoles 1 with two *N*-allenamides 146 and 147 was investigated by Pirovano *et al.* to prepare tetrahydrocarbazole 148 in 78% yield (Scheme 65).^131^

**Scheme 66:**
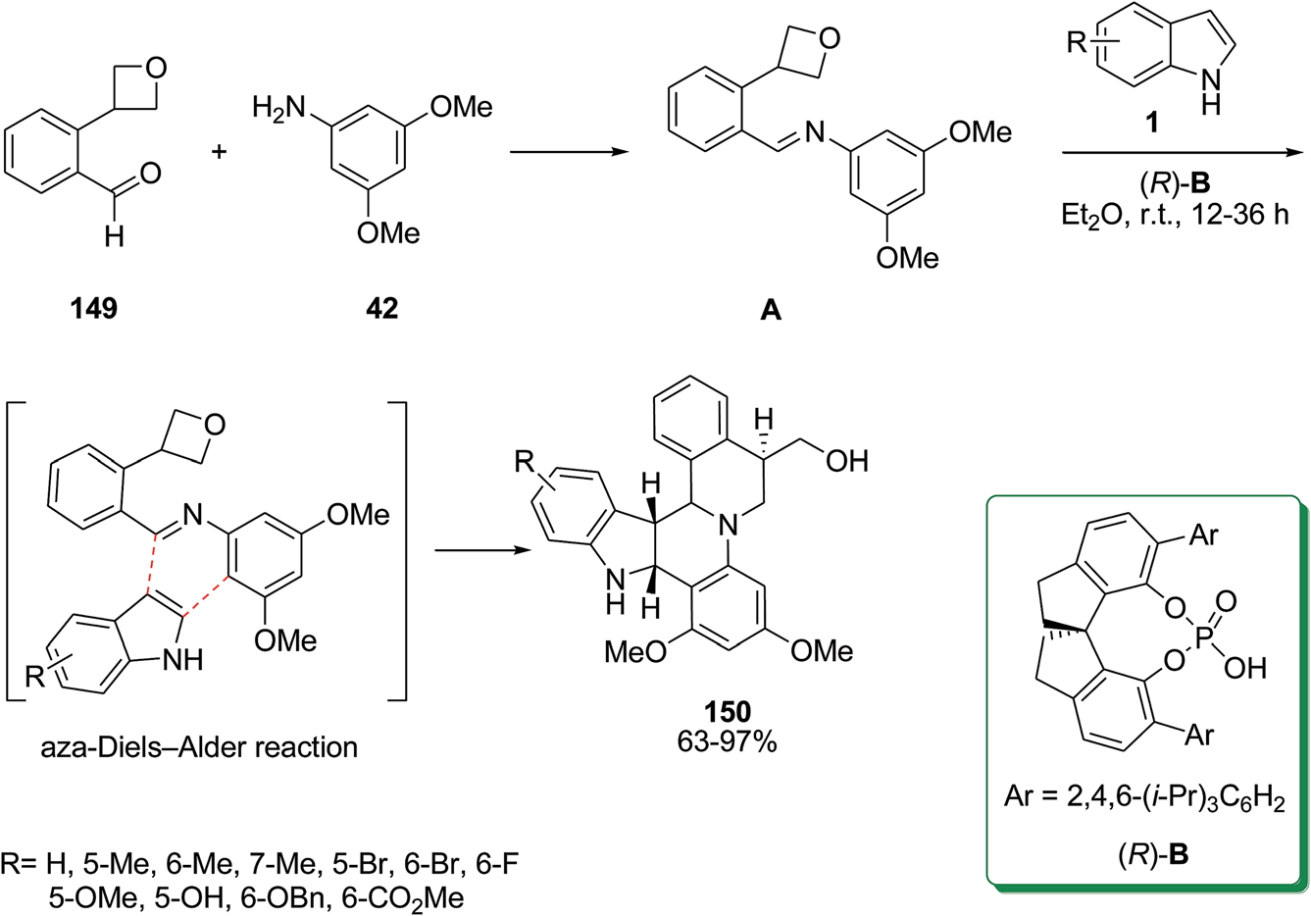


Chiral phosphoric acid was used as the catalyst in the asymmetric aza-Diels–Alder reaction^132^ of indole derivatives 1 with 2-azadiene generated *in situ* from oxetane 149^133^ and 3,5-dimethoxyaniline 42 to obtain a series of indole-alkaloid-type polycycles 150 (Scheme 66).^134^ The authors hypothesized that the introduction of a hydrogen-bond acceptor on the aldehyde moiety may help to orient the transition state and lower the activation barrier for the desired process. Thus, they employed substrates with a simple ether group in proximity to the aldehyde moiety, and encouragingly, the desired aza-Diels–Alder reaction proceeded cleanly to form the desired products with good yields and diastereoselectivities. The biological activity evaluation of the products showed that the cytotoxicities of products in human lung carcinoma and human cervical carcinoma cells exhibited inhibitory effects against cell proliferation with IC_50_ values in the range of 15.0–27.5 μM.

**Scheme 67:**
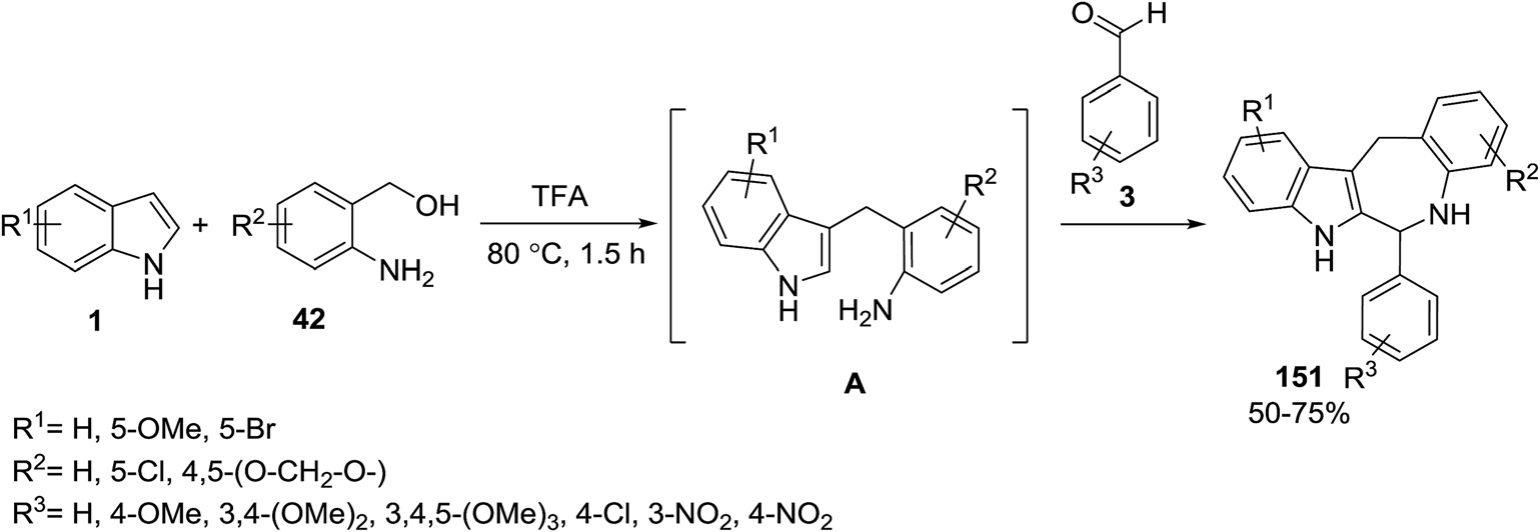


The annulation of indoles 1, 2-amino benzyl alcohols 42 and benzaldehydes 3 in a two step three-component tandem reaction was used to form benzazepinoindoles 151. In the first step, indoles were C-3 alkylated and the intermediates A were obtained. Then in the second step, intermediates A underwent a 7-*endo*-trig cyclization (the modified Pictet–Spengler cyclization reaction) to obtain the intended products 151 (Scheme 67).^135^

**Scheme 68:**
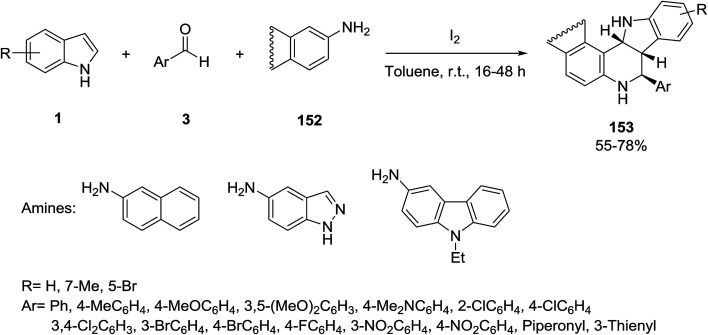


A stereoselective Povarov reaction^136,137^ catalyzed by iodine was developed by Wang and co-workers for the preparation of *exo*-tetrahydroindolo[3,2-*c*]quinoline derivatives 153. The procedure involved a reaction between indoles 1, aldehydes 3 and amines 152 in toluene (Scheme 68).^138^ The results showed that only reactive amines could be included in this reaction to give the desired products 153 with high stereoselectivity.

**Scheme 69:**
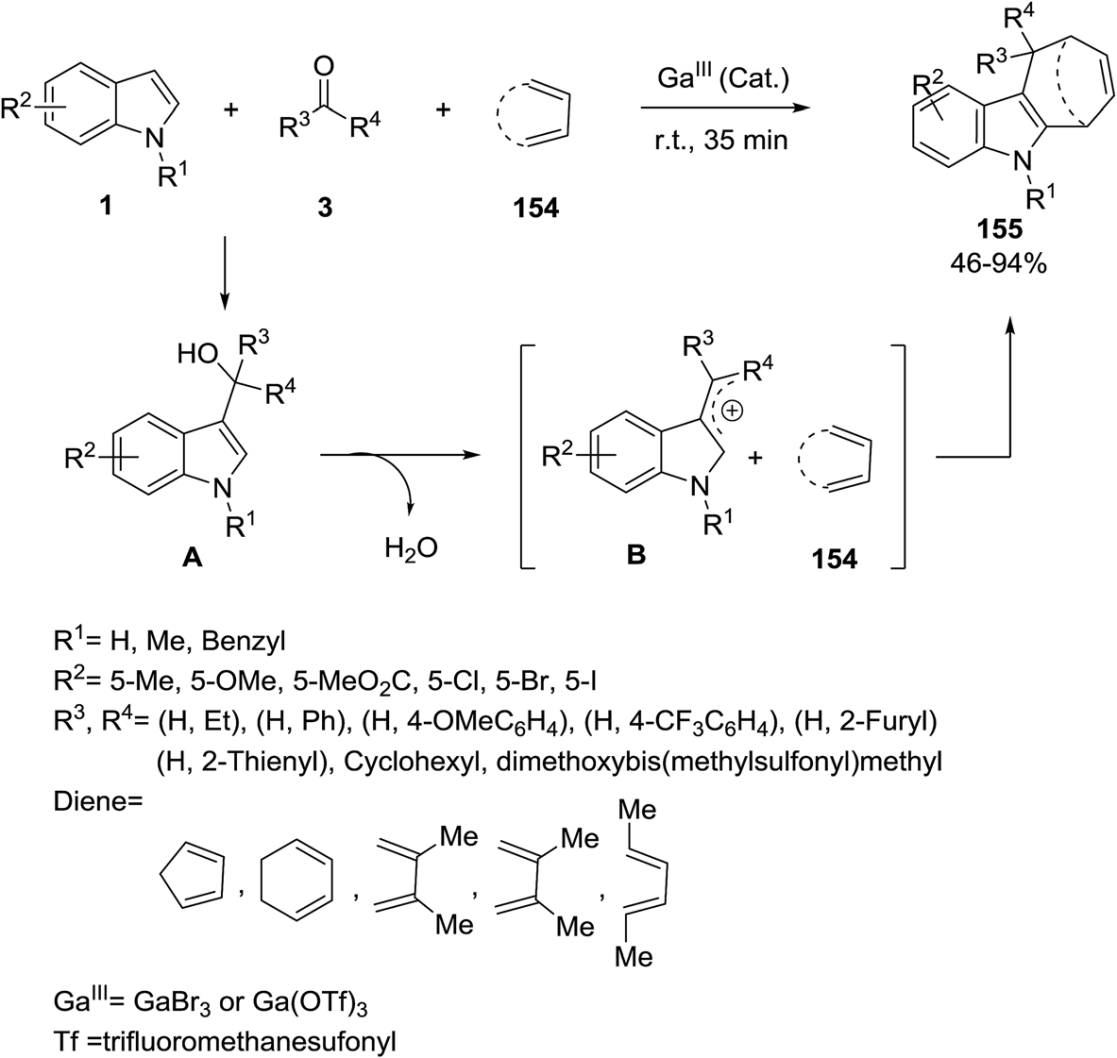


Gallium(iii) (GaBr_3_) catalyzed a three component [4 + 3] cycloaddition reaction of indoles 1, aldehyde/ketone/ketal derivatives 3 and dienes 154 to furnish cyclohepta[*b*]indoles 155. The authors have proposed the mechanism of formation of the product *via* nucleophilic addition of the C3 of indole to electrophile 3 to give the alcohol A, which in the presence of a Lewis acid generated the indolyl cation B. The treatment of B with diene component 154 afforded the corresponding products (Scheme 69).^139^

**Scheme 70:**
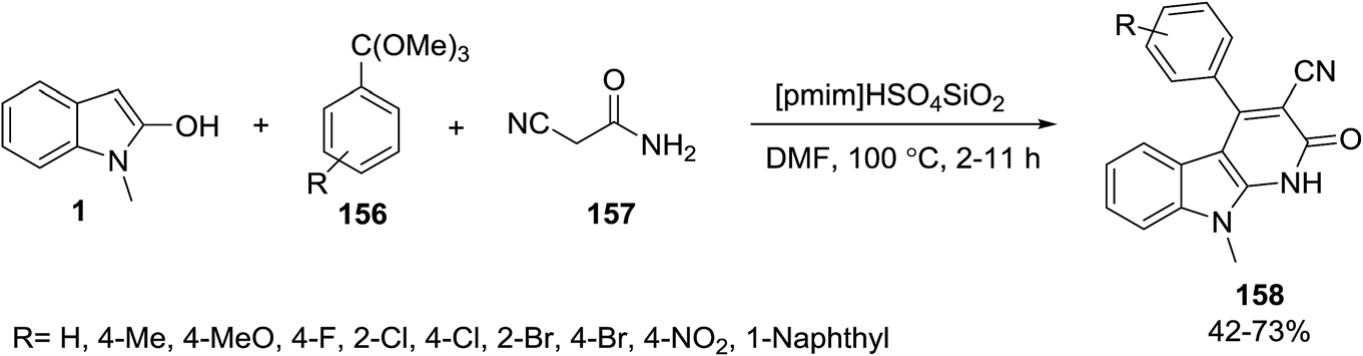


Damavandi and Sandaroos have synthesized 2,9-dihydro-2-oxo-4-aryl-1*H*-pyrido[2,3-*b*]indole-3-carbonitriles 158 by the one-pot multicomponent cyclocondensation reaction of 1-methyl-1*H*-indol-2-ol 1, substituted (triethoxymethyl) arenes 156 and cyanoacetamide 157 in the presence of silica supported ionic liquid [pmim]HSO_4_SiO_2_ as a catalyst (Scheme 70).^140^

**Scheme 71:**
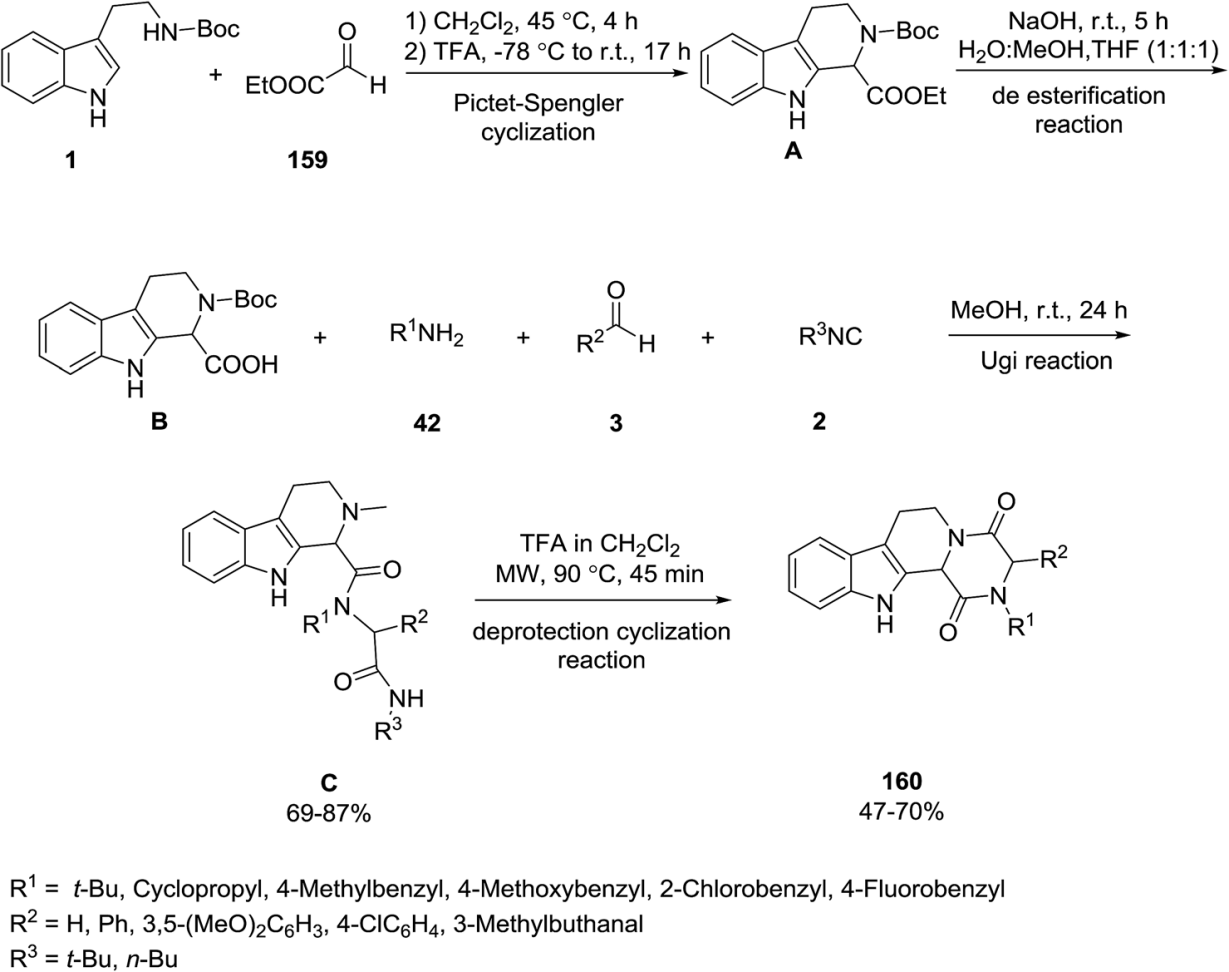


A new series of diketopiperazine-fused tetrahydro-β-carboline ring systems 160 were obtained *via* the Pictet–Spengler/Ugi-4CR/deprotection-cyclization reactions. According to the proposed mechanism, the Pictet–Spengler cyclization of indole 1 and ethyl glyoxalate 159 followed by a deesterification reaction produced 2,3,4,9-tetrahydro-1*H*-pyrido[3,4-*b*]indole-1-carboxylic acid B. The reaction of acid B, amines 42, isocyanides 2 and aldehyde 3*via* the Ugi reaction gave the intermediate products C. The deprotection cyclization reaction of this intermediate generated the target products 160 (Scheme 71).^141^

**Scheme 72:**
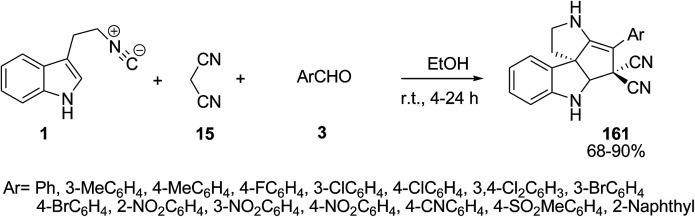


A stereoselective catalyst-free three-component reaction of 2-isocyanoethylindole 1, malononitrile 15 and aldehyde 3 was developed for the construction of polycyclic spiroindolines 161 in high yields (up to 90%) with excellent levels of diastereoselectivity (Scheme 72).^142^ The presence of an electron-withdrawing group on the aldehyde led to a decrease in yields of the products.

**Scheme 73:**
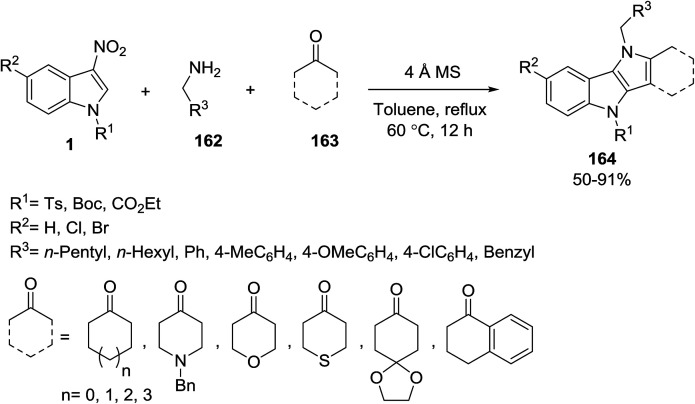


John and co-workers developed a multicomponent reaction involving *N*-protected 3-nitroindole 1 a primary amine 162 and an enolizable ketone 163 for the preparation of a series of functionalized pyrrolo[3,2-*b*]indoles 164 (Scheme 73).^143^ It was found that the yield of product 164 decreased to 45% with 1-phenylethylamine. With cyclohexylamine, the product 164 was obtained in moderate yield (53%) and with adamantylamine the MCR failed, proving that the reactivity decreases with an increase in the steric bulk of the amine component.

**Scheme 74:**
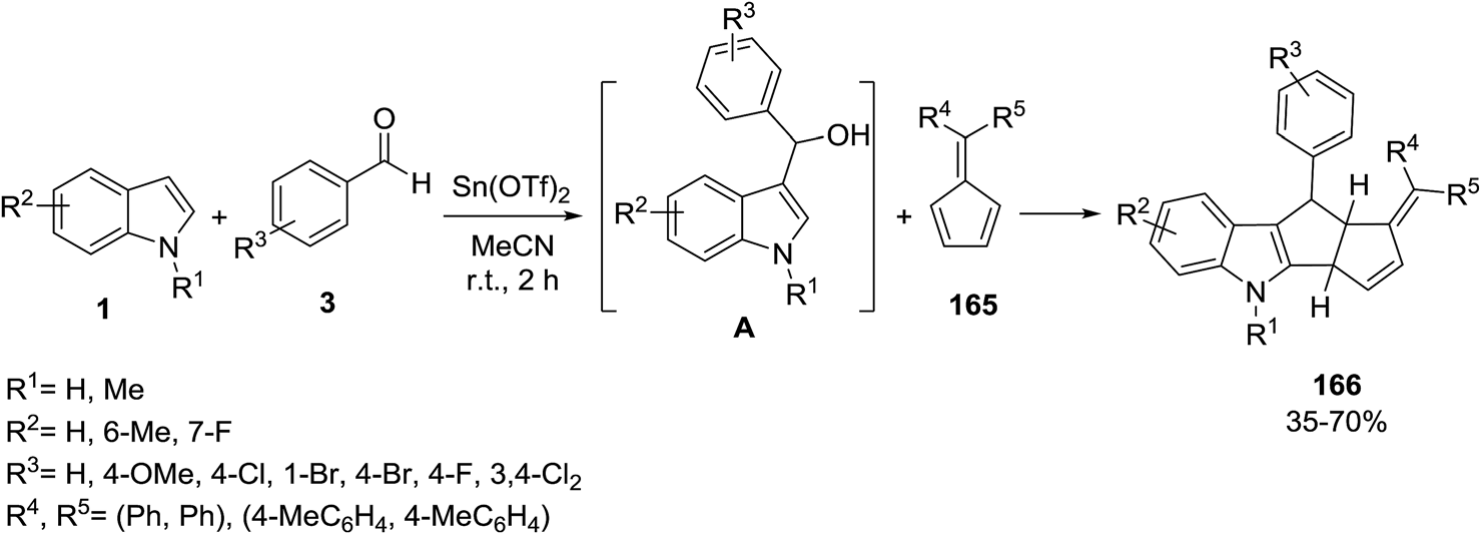


The Lewis acid catalyzed three-component [3 + 2] cycloaddition reaction of pentafulvene 165 with *in situ* generated indolylmethanol A has been developed for the construction of pentaleno[1,2-*b*]indoles 166 (Scheme 74).^144^ It was found that aromatic aldehydes bearing electron-withdrawing groups (R^3^ = Cl, Br, F) were tolerated well under the reaction conditions and afforded the cycloaddition products in moderate to good yields. However, aldehydes with electron-rich substituents (R^3^ = 2,4-OMe, Me) were unable to take part in the cycloaddition reaction.

**Scheme 75:**
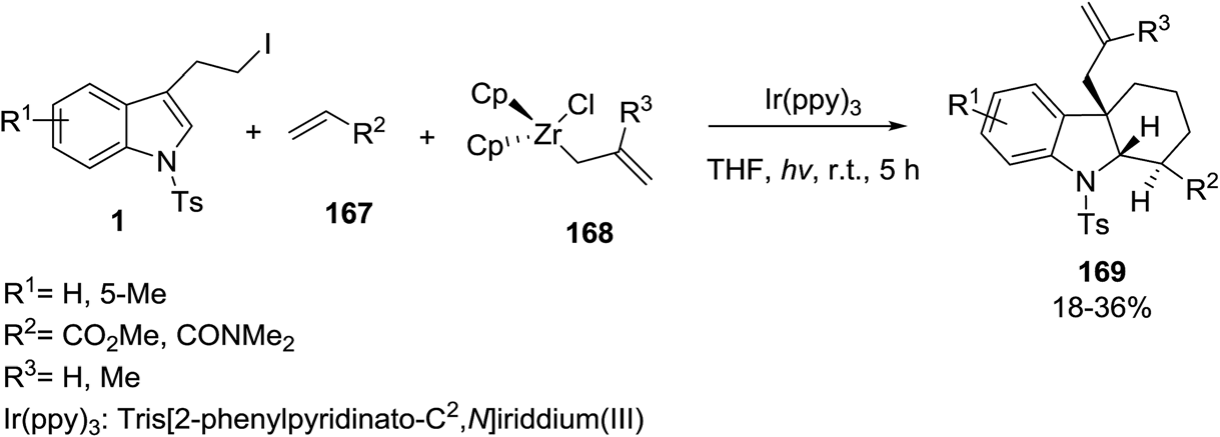


Alpers *et al.* developed a photoinduced three-component radical [4 + 2]-cyclization–allylation reaction between 3-(2-iodoethyl)indoles 1, acceptor-substituted alkenes 167, and allyl zirconocenes of the structure Cp ZrCl(σ-allyl) 168 for the synthesis of hexahydrocarbazoles 169 (Scheme 75).^145^

**Scheme 76:**
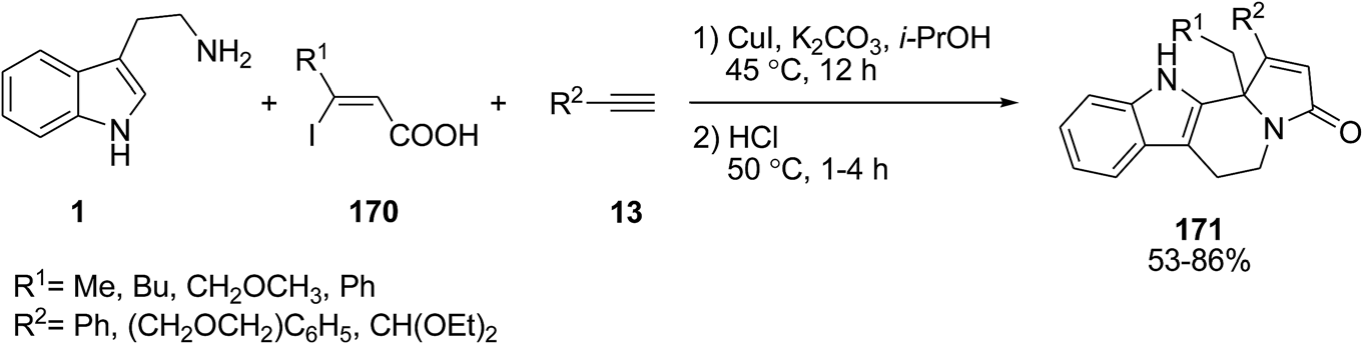


A copper(i)-catalyzed cascade multicomponent reaction strategy was performed for the construction of 5-hydroxy-1*H*-pyrrol-2(5*H*)-ones 171 bearing an indole moiety. The reaction was carried out using substituted indole 1, (*Z*)-3-iodoacrylic acids 170 and terminal alkynes 13 (Scheme 76).^146^

#### 2.3.2. The cycloaddition reactions of indoles at the C–N sigma bond

**Scheme 77:**
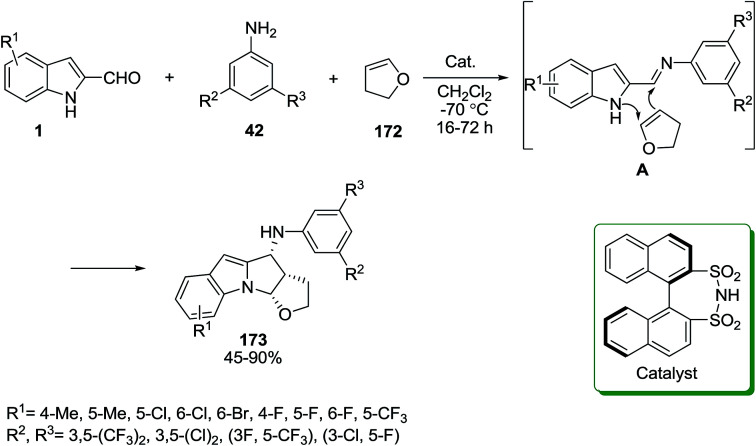


An enantioselective multicomponent coupling reaction for the synthesis of pyrrolo[1,2-*a*]indoles 173 was reported using a chiral disulfonimide as catalyst. The [3 + 2] cyclization reaction between the imine A (generated *in situ* from indole-2-aldehyde derivatives 1 and anilines 42) and 2,3-dihydrofuran 172 led to products 173 (Scheme 77).^147^ It was observed that the yields of products synthesized from substituted anilines at the 3- and 5-positions with halogen atoms groups were lower than those synthesized from 3,5-bis(trifluoromethyl)aniline.

**Scheme 78:**
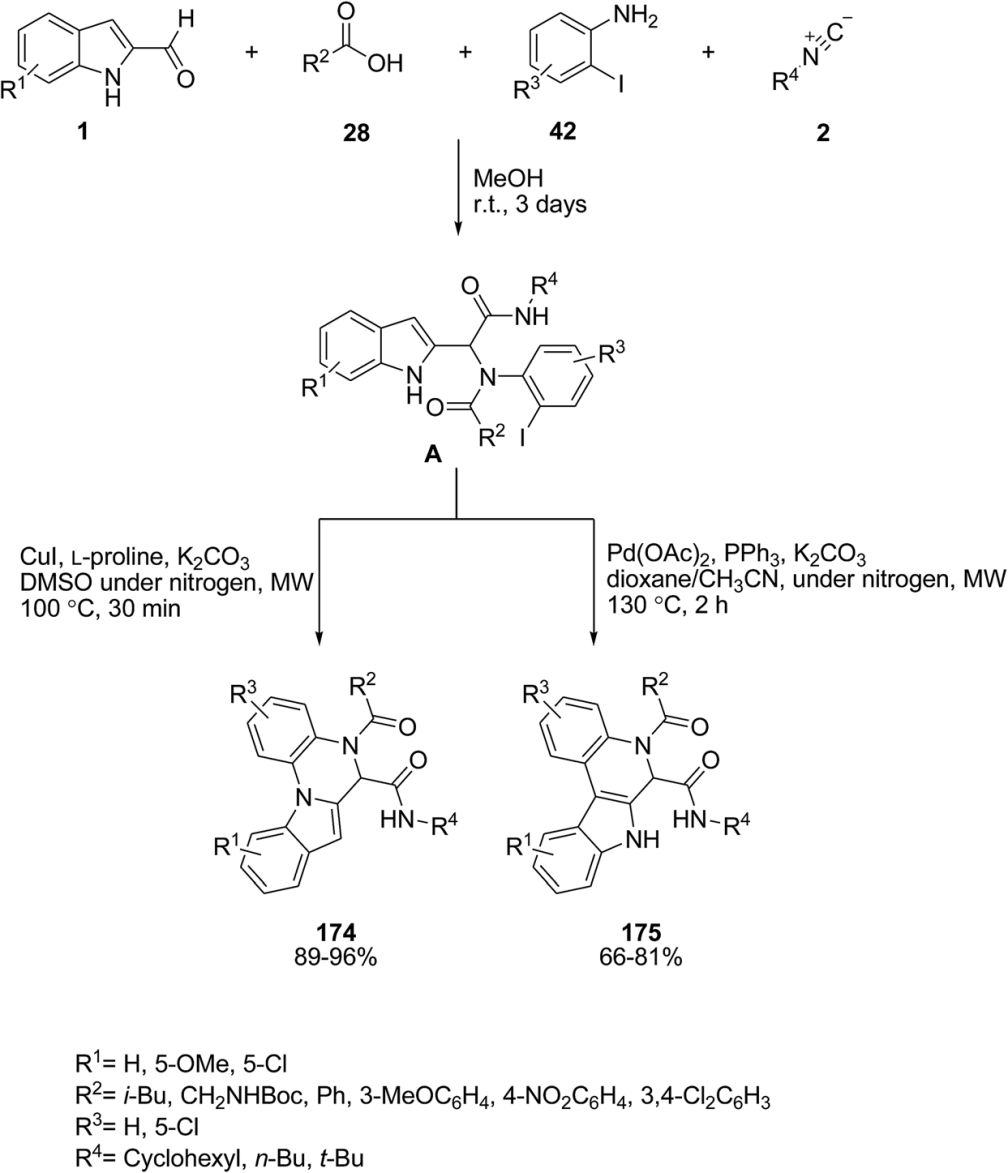


The Ugi four-component condensation of indole-2-aldehyde derivatives 1, acids 28, anilines 42 and isocyanides 2 in the presence of orthogonal copper and palladium catalysts under microwave heating was accomplished for the synthesis of two important indole-fused heterocycles 174 and 175. First, the Ugi adduct A was obtained *via* the intramolecular cyclization conditions and served as a precursor in subsequent selective post-transformations. 5,6-Dihydroindolo[1,2-*a*]quinoxalines 174 were prepared by a copper catalyzed N–H arylation pathway, while 6,7-dihydroindolo[2,3-*c*]quinolones 175 were formed by palladium catalyzed C–H arylations without the protection of the indole N1 moiety (Scheme 78).^148^ In another approach to synthesize these compounds, no catalyst has been employed in this reaction and the products were obtained in good yields.^149^

### 2.4. Miscellaneous reactions of indoles

**Scheme 79:**
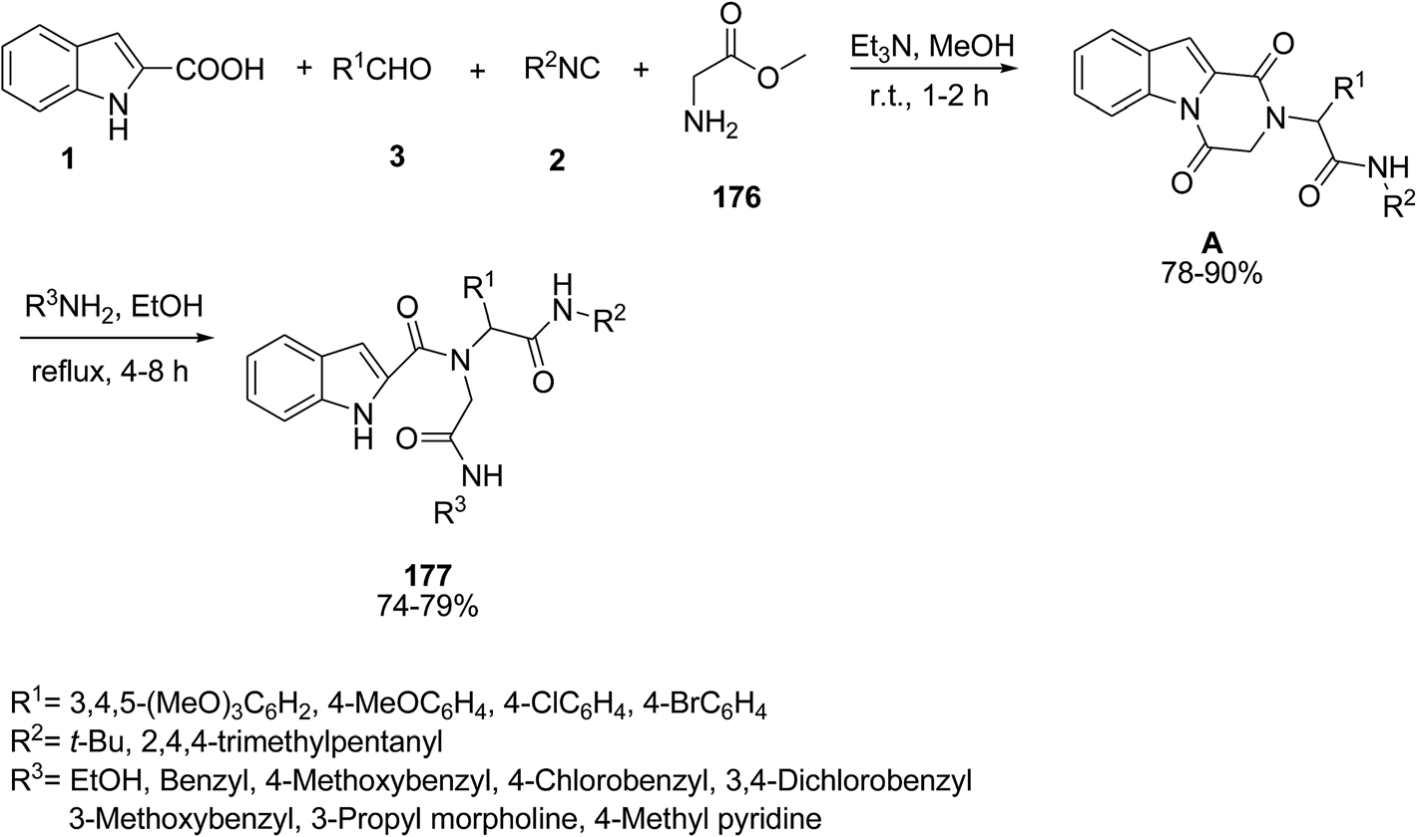


Pandey *et al.* have published their results on the synthesis of diverse indole-2-carboxamides 177*via* a two-step multicomponent reactions. Initially, Et_3_N was added to amino ester hydrochloride 176 and after 10 minutes, 1*H*-indole-2-carboxylic acid 1, aldehyde 3, and isocyanide 2 were successively added to the mixture to produce corresponding indole-fused diketopiperazines A.^150^ Then, compound A in the presence of amines formed the functionalized carboxamides 177 (Scheme 79).^151^ The biological activities of products were evaluated and showed that most products have anti-leishmanial activity against intracellular amastigotes form of *Leishmania donovani*.

**Scheme 80:**
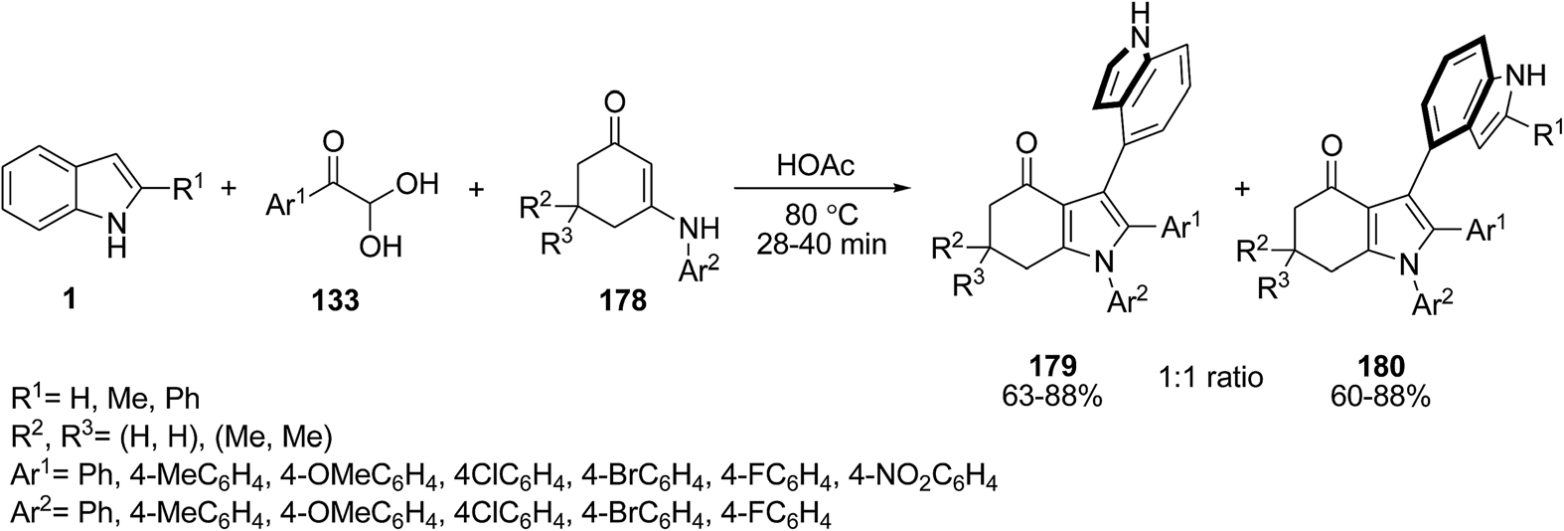


Fu and co-workers designed a strategy for the preparation of 3,2′- 179 and 3,3′-bis-indoles 180 through the microwave-assisted regioselective reaction of indoles 1, arylglyoxal monohydrate 133 and diverse *N*-aryl enaminones 178 in HOAc (Scheme 80).^152^ The 2-unsubstituted indoles resulted in 3,2′-bis-indole frameworks 179, while indoles bearing a methyl or phenyl group at C2 led to the 3,3′-bis-indoles 180 with simultaneous formation of three sigma-bonds.

## 3. Conclusion and outlook

This review summarizes recent studies on the application of indoles in multicomponent reactions for the synthesis of a variety of heterocyclic compounds during the period of 2012 to 2017. Indole is a significant nitrogen-based heterocycle with particular importance in the synthesis of complex heterocyclic scaffolds. Indole fragments have been recently attracting much attention due to their biological and pharmaceutical activities. Diversely multisubstituted indole substances are useful building blocks in pharmaceutical and organic syntheses. Consequently, the novel methodologies for the synthesis of complex heterocyclic frameworks involving indole are expected to receive further increasing attention in the future.

## 4. Conflicts of interest

There are no conflicts to declare.

## 5. Abbreviations

CTAB: Cetyltrimethylammonium bromide
DCE: 1,2-Dichloroethylene
DIPEA: *N*,*N*-Diisopropylethylamine
DMAD: Dialkyl acetylenedicarboxylate
DMF: Dimethylformamide
DPP: Diketopyrrolopyrrole
EA: Ethyl acetate
FHS: Ferric hydrogen sulfate
MTBE: Methyl *tert*-butyl ether
mpCuO: Macroporous copper oxide
MW: Microwave
NHC: *N*-Heterocyclic carbene
NPs: Nanoparticles
PEG: Polyethylene glycol
PMDETA: Pentamethyldiethylenetriamine
PTS: Polyoxyethanyl-α-tocopheryl sebacate
SDS: Sodium dodecyl sulfate
STA: Silica-supported tungstic acid
TBAF: Tetra-*n*-butylammonium fluoride
TFA: Trifluoroacetic acid
THF: Tetrahydrofuran

## Supplementary Material
